# Global, regional and national burden of decubitus ulcers in 204 countries and territories from 1990 to 2021: a systematic analysis based on the global burden of disease study 2021

**DOI:** 10.3389/fpubh.2025.1494229

**Published:** 2025-02-26

**Authors:** Shenyue Zhang, Guoxing Wei, Liu Han, Weibing Zhong, Zhentan Lu, Zehao Niu

**Affiliations:** ^1^Department of Biomedical Sciences, Jockey Club College of Veterinary Medicine and Life Sciences, City University of Hong Kong, Kowloon, Hong Kong SAR, China; ^2^Department of Emergency, The 83 Affiliated Hospital of Xinxiang Medical University, Xinxiang, China; ^3^Department of Burns and Plastic Surgery, The 83 Affiliated Hospital of Xinxiang Medical University, Xinxiang, China; ^4^Department of Plastic Surgery, Southwest Hospital, Third Military Medical University (Army Medical University), Chongqing, China; ^5^Key Laboratory of Textile Fiber and Products (Wuhan Textile University) Ministry of Education, Hubei International Scientific and Technological Cooperation Base of Intelligent Textile Materials & Application, Wuhan Textile University, Wuhan, China

**Keywords:** decubitus ulcers, pressure ulcers, burden, trends, systematic analysis

## Abstract

**Background:**

Decubitus ulcers, also known as pressure ulcers, pose a significant public health challenge due to their substantial impact on morbidity, mortality, and healthcare expenditures.

**Methods:**

The number and age-standardized rates (ASRs) of prevalence, death, disability adjusted life-year (DALY), years of life lost (YLL), and years lived with disability (YLD) at the global, regional, and national levels were acquired from the GBD 2021 database. Trends were evaluated based on the estimated average percentage change (EAPC) of ASRs. Additionally, data were stratified by socio-demographic index (SDI) quantiles, regions, countries, territories, and age groups.

**Results:**

The total number of decubitus ulcer cases increased from 300,442 in 1990 to 645,588 in 2021. The global ASR of prevalence decreased slightly from 8.25 to 7.92 per 100,000 persons, with most cases occurring in individuals aged 60 and older. Deaths due to decubitus ulcers rose from 16,622 in 1990 to 37,033 in 2021, while the global ASR of death declined from 0.53 to 0.46 per 100,000 persons. DALY due to decubitus ulcers increased, exhibiting significant variation across regions and age groups. A higher SDI was correlated with increased ASRs of prevalence (*R* = 0.488, *p* < 0.001) and YLD (*R* = 0.495, *p* < 0.001). Conversely, a higher SDI was correlated with lower ASRs of death (*R* = −0.329, *p* < 0.001), DALY (*R* = −0.398, *p* < 0.001), and YLL (*R* = −0.445, *p* < 0.001).

**Conclusion:**

The global burden of decubitus ulcers has risen, with notable regional and age-related disparities. This study offers valuable insights for policymakers to optimize healthcare strategies and mitigate the public health impact of decubitus ulcers.

## 1 Introduction

Decubitus ulcers, also known as pressure ulcers, represent a significant global public health concern due to their considerable impact on morbidity, mortality, and healthcare costs (1). These lesions, caused by prolonged pressure on the skin, are particularly prevalent among individuals with limited mobility, such as aging individuals and those with chronic illnesses. As a type of chronic wound, decubitus ulcers are characterized by poor healing and frequent recurrence, leading to a reduced quality of life and substantial medical expenses (2, 3). Despite advancements in medical knowledge and treatment, decubitus ulcers remain a prevalent and debilitating issue, creating significant challenges for healthcare systems and socioeconomic structures (4, 5). Studies have shown a high prevalence of decubitus ulcers, with estimates indicating a prevalence of approximately 12% among hospitalized patients (6) and even higher rates in intensive care units (7). Therefore, understanding the latest spatial distribution and temporal trends of decubitus ulcers is essential for developing effective prevention and treatment programs to improve patient outcomes and reduce unnecessary medical costs.

Several studies have assessed the burden of decubitus ulcers at regional and national levels. For example, Moore et al. (8) conducted a systematic review concerning the prevalence of decubitus ulcers in Europe. Källman et al. (9) analyzed hospital-based prevalence trends in Sweden using nationwide surveys over 10 years. At the global level, Yakupu et al. (10) and Zhang et al. (11) reported the burden of decubitus ulcers from 1990 to 2019 based on the Global Burden of Disease (GBD) 2019 database. While previous studies have contributed valuable insights into the burden of decubitus ulcers, they also have some limitations. First, research focusing on regional data lacks a global perspective, limiting its ability to identify worldwide trends and compare regional disparities. Second, studies by Yakupu et al. and Zhang et al. only analyzed data up to 2019, failing to account for more recent trends and the potential impact of the coronavirus disease 2019 (COVID-19) pandemic, which may have significantly influenced the burden of decubitus ulcers.

The GBD 2021 database offers the most up-to-date epidemiological data on various diseases and injuries across 204 countries and territories from 1990 to 2021. This study aims to analyze data on the prevalence, death, disability adjusted life-year (DALY), years of life lost (YLL), years lived with disability (YLD) due to decubitus ulcers, considering factors such as location, sex, and the sociodemographic index (SDI). This study aims to inform policymakers and optimize strategies for decubitus ulcer management and prevention by providing a comprehensive and comparable analysis of the burden of decubitus ulcers.

## 2 Method

### 2.1 Study design and data resources

This observational study is based on the GBD 2021 database^1^. The database provided comprehensive information on 288 causes of death, 371 diseases and injuries, and 88 risk factors in 204 countries and territories from 1990 to 2021. In summary, the GBD 2021 study employed data sources such as national censuses, demographic surveys, disease surveillance points, causes of death registries, and comprehensive reviews of published literature to gather information on disease incidence and prevalence. Since not all countries provided data for every disorder, the study utilized DisModMR 2.1, a Bayesian meta-regression tool, as its principal method for estimating and ensuring coherence among incidence, prevalence, and mortality rates across various health conditions. Uncertainty intervals (UIs) were calculated for all metrics, measuring the statistical confidence in the estimates.

### 2.2 Case definition and categorization

In the GBD 2021 database, decubitus ulcers were classified in skin and subcutaneous conditions and were coded as L89 in the International Classification of Disease 10th (ICD-10) code. The severity of decubitus ulcers can be divided into four stages. Stage 1 is characterized by non-blanchable erythema over intact skin, indicating localized inflammation and potential tissue damage. Stage 2 involves partial-thickness skin loss, presenting as an abrasion, blister, or shallow crater. In Stage 3, full-thickness skin loss extends to the subcutaneous tissue, with visible fat but no muscle, tendon, or bone exposed. Stage 4 represents the most severe form, with extensive tissue loss exposing muscle, tendon, or bone.

### 2.3 Compilation of results

Various indicators were used in the decubitus ulcers burden evaluation, including the number and age-standardized rates (ASRs) of prevalence, death, disability adjusted life-year (DALY), years of life lost (YLL), years lived with disability (YLD) at the global, regional and national levels. The prevalence rate was defined as the number of aggregated cases per 100,000 people, while the death rate was the number of annual deaths per 100,000 people. YLL is a measure of premature mortality calculated by subtracting the age at death from the highest achievable life expectancy for each age group. YLD measures the time lived with a disability or health condition, calculated by multiplying the disability’s severity by its duration. DALY is a composite measure reflecting the total years lost due to ill health, disability, or early death. DALY is the sum of YLL and YLD. Prevalence, death, and DALY trends were assessed using Estimated Average Percentage Changes (EAPCs) in ASRs. The natural logarithm of ASR was assumed to be linear over time, with EAPCs calculated from this model. An upward trend was indicated if the 95% confidence interval (CI) of EAPC estimation was >0, a downward trend if the 95% CI was <0, and stability if the 95% CI included 0.

Data were stratified by 5 SDI quantiles, 21 regions, and 204 countries and territories. The Socio-demographic index (SDI) is an indicator used in the evaluation of the development status of each country. It ranges from 0 to 1 and was calculated based on the total fertility rate under 25, average education over 15 years old, and lag-distributed income per person. The present study used SDI values provided by GBD 2021 data to categorize countries into quintiles: low, low-middle, medium, high-middle, and high quintiles. The data were stratified by age with an interval of 5 years to evaluate the burden across different age groups.

### 2.4 Statistical analysis

Statistical analyses and visualizations were conducted using R software (V.4.3.3). Pearson’s correlation analysis was performed to identify the association between the burden of decubitus ulcers and the SDI. The map was produced by using the package ‘ggmap’ package. A *p* value <0.05 was considered statistically significant.

## 3 Results

### 3.1 The prevalence of decubitus ulcers in 2021 and 1990

The total number of patients with decubitus ulcers increased from 300,442 (270,738 to 333,579) in 1990 to 645,588 (582,432 to 712,876) in 2021 (Supplementary Table S1). However, the global ASR of prevalence slightly decreased from 8.25 (7.39 to 9.18) in 1990 to 7.92 (7.14 to 8.73) in 2021, with an EAPC of −0.02% (−0.1 to 0.06) (Table 1).

**Table 1:** Prevalence, death and disability adjusted life-year (DALY) for decubitus ulcer in 1990 and 2021 and their temporal trends from 1990 to 2021 at the global, SDI quintile and GBD region levels.

Location	Age-standardized prevalence rate			Age-standardized death rate			Age-standardized DALY rate		
	1990 (95% UI)	2021 (95% UI)	EAPC (95% CI)	1990 (95% UI)	2021 (95% UI)	EAPC (95% CI)	1990 (95% UI)	2021 (95% UI)	EAPC (95% CI)
Global	8.25	7.92	-0.02%	0.53	0.46	−0.58%	10.74	9.7	−0.58%
	(7.39 to 9.18)	(7.14 to 8.73)	(−0.1 to 0.06)	(0.45 to 0.62)	(0.36 to 0.52)	(−0.71 to −0.46)	(8.87 to 12.74)	(7.41 to 10.88)	(−0.77 to −0.39)
SDI quintile									
High SDI	14.94	14.41	−0.18%	0.59	0.26	−2.47%	10.96	6.66	−1.89%
	(13.37 to 16.68)	(13.04 to 15.83)	(−0.23 to −0.13)	(0.52 to 0.63)	(0.22 to 0.28)	(−2.73 to −2.2)	(10.05 to 11.98)	(5.87 to 7.53)	(−2.05 to −1.73)
High-middle SDI	5.47	5.64	0.28%	0.25	0.42	2.09%	4.57	7.24	1.59%
	(4.88 to 6.12)	(5.09 to 6.24)	(0.15 to 0.42)	(0.22 to 0.29)	(0.33 to 0.47)	(1.75 to 2.44)	(4.12 to 5.23)	(5.8 to 8.09)	(1.19 to 1.99)
Middle SDI	5.67	6.83	0.85%	0.53	0.53	−0.18%	10.15	9.8	−0.23%
	(5.12 to 6.27)	(6.16 to 7.52)	(0.71 to 0.99)	(0.43 to 0.67)	(0.39 to 0.62)	(−0.24 to −0.12)	(8.26 to 12.48)	(7.28 to 11.15)	(−0.33 to −0.12)
Low-middle SDI	3.17	4.08	1.16%	0.63	0.7	−0.28%	13.99	13.87	−0.46%
	(2.82 to 3.51)	(3.69 to 4.51)	(0.98 to 1.33)	(0.39 to 0.85)	(0.46 to 0.87)	(−0.58 to 0.02)	(8.67 to 18.65)	(9.21 to 17.12)	(−1 to 0.09)
Low SDI	1.49	1.59	0.44%	0.84	0.77	−0.50%	19.55	16.45	−0.88%
	(1.3 to 1.69)	(1.38 to 1.79)	(0.29 to 0.59)	(0.52 to 1.38)	(0.51 to 1.03)	(−0.64 to −0.36)	(11.9 to 30.98)	(10.55 to 22.02)	(−1.11 to −0.66)
GBD region									
Andean Latin America	7.7	6.72	−0.57%	0.61	0.46	−1.94%	12	8.19	−2.44%
	(6.95 to 8.61)	(6.02 to 7.46)	(−0.62 to −0.52)	(0.45 to 0.77)	(0.36 to 0.63)	(−2.56 to −1.32)	(8.87 to 14.57)	(6.49 to 10.69)	(−3.3 to −1.57)
Australasia	4.86	4.38	−0.42%	0.25	0.1	−2.56%	4.06	1.95	−3.35%
	(4.34 to 5.42)	(3.91 to 4.9)	(−0.53 to −0.32)	(0.21 to 0.27)	(0.08 to 0.11)	(−3.28 to −1.84)	(3.64 to 4.47)	(1.65 to 2.25)	(−3.95 to −2.74)
Caribbean	11.54	10.08	−0.48%	2.02	1.41	−1.28%	36.67	29.72	−0.83%
	(10.43 to 12.72)	(9.13 to 11.08)	(−0.5 to −0.46)	(1.8 to 2.31)	(1.19 to 1.71)	(−1.38 to −1.18)	(31.98 to 43.39)	(24.26 to 36.76)	(−0.97 to −0.7)
Central Asia	1.2	1.18	0%	0.01	0.03	3.18%	0.4	0.66	2.07%
	(1.04 to 1.38)	(1.03 to 1.36)	(−0.04 to 0.04)	(0.01 to 0.01)	(0.02 to 0.03)	(2.43 to 3.94)	(0.31 to 0.51)	(0.56 to 0.79)	(1.46 to 2.69)
Central Europe	12.25	13.38	0.40%	0.05	0.11	1.97%	2.98	4.01	1.76%
	(10.87 to 14.02)	(12.1 to 14.86)	(0.33 to 0.48)	(0.05 to 0.06)	(0.09 to 0.12)	(1.06 to 2.9)	(2.39 to 3.69)	(3.29 to 4.7)	(1.41 to 2.11)
Central Latin America	23.54	21.89	−0.25%	1.35	0.52	−2.53%	28.02	12.15	−2.59%
	(21.08 to 26.12)	(19.63 to 24.19)	(−0.29 to −0.21)	(1.27 to 1.41)	(0.43 to 0.64)	(−2.74 to −2.33)	(26.55 to 29.63)	(10.23 to 14.63)	(−2.75 to −2.43)
Central Sub-Saharan Africa	1.06	0.94	−0.38%	1.28	1.28	−0.23%	26.08	24.89	−0.46%
	(0.92 to 1.21)	(0.82 to 1.08)	(−0.41 to −0.36)	(0.43 to 1.93)	(0.52 to 2.01)	(−0.34 to −0.13)	(8.63 to 40.04)	(10.26 to 38.96)	(−0.65 to −0.28)
East Asia	4.67	5.76	1.03%	0.15	0.22	1.28%	2.79	3.88	1.03%
	(4.17 to 5.22)	(5.21 to 6.37)	(0.82 to 1.24)	(0.11 to 0.28)	(0.13 to 0.29)	(0.59 to 1.99)	(2.2 to 4.5)	(2.44 to 4.74)	(0.17 to 1.91)
Eastern Europe	6.81	6.08	−0.37%	0.02	0.12	4.79%	1.64	3.78	2.57%
	(6.07 to 7.67)	(5.43 to 6.79)	(−0.45 to −0.28)	(0.02 to 0.02)	(0.11 to 0.13)	(4.18 to 5.39)	(1.26 to 2.07)	(3.43 to 4.24)	(2.14 to 3)
Eastern Sub-Saharan Africa	0.51	0.47	−0.52%	1.84	1.66	−0.38%	41.07	34.69	−0.81%
	(0.43 to 0.61)	(0.39 to 0.56)	(−0.66 to −0.39)	(1.03 to 3.58)	(1.12 to 2.49)	(−0.45 to −0.3)	(23.89 to 77.51)	(22.73 to 53.24)	(−0.91 to −0.71)
High-income Asia Pacific	12.82	12.89	0.15%	0.26	0.17	−3.14%	5.79	4.47	−0.93%
	(11.53 to 14.17)	(11.61 to 14.17)	(0.06 to 0.25)	(0.22 to 0.29)	(0.13 to 0.19)	(−3.67 to −2.61)	(4.91 to 6.73)	(3.74 to 5.31)	(−1.22 to −0.64)
High-income North America	31.01	30.82	−0.08%	0.47	0.21	−2.35%	12.4	8.28	−1.17%
	(27.49 to 34.76)	(27.77 to 33.94)	(−0.18 to 0.01)	(0.42 to 0.5)	(0.18 to 0.24)	(−2.57 to −2.12)	(10.9 to 14.11)	(6.84 to 9.8)	(−1.26 to −1.08)
North Africa and Middle East	2.95	2.81	−0.02%	0.72	0.83	0.44%	13.35	14.57	0.30%
	(2.59 to 3.3)	(2.49 to 3.13)	(−0.18 to 0.14)	(0.51 to 1.1)	(0.65 to 0.99)	(0.39 to 0.5)	(9.82 to 19.39)	(11.61 to 17.23)	(0.24 to 0.35)
Oceania	1.06	1.09	0.09%	1.05	1.08	0.01%	22.08	22.81	0.16%
	(0.93 to 1.21)	(0.98 to 1.22)	(0.09 to 0.1)	(0.5 to 2.42)	(0.45 to 2.02)	(−0.04 to 0.06)	(9.63 to 49.5)	(8.61 to 45.24)	(0.09 to 0.24)
South Asia	2.35	3.05	1.24%	0.5	0.52	−1.20%	12.36	10.91	−1.33%
	(2.03 to 2.68)	(2.63 to 3.46)	(0.99 to 1.49)	(0.28 to 0.76)	(0.29 to 0.7)	(−1.89 to −0.5)	(7.33 to 17.78)	(6.64 to 14.34)	(−2.48 to −0.16)
Southeast Asia	1.2	1.45	0.71%	0.98	1.42	0.71%	18.82	23.85	0.75%
	(1.07 to 1.37)	(1.31 to 1.6)	(0.63 to 0.79)	(0.66 to 1.5)	(0.91 to 1.72)	(0.51 to 0.91)	(12.92 to 27.91)	(14.94 to 28.79)	(0.56 to 0.94)
Southern Latin America	3.88	4.95	1.09%	0.85	1.87	3.10%	13.69	28.05	3.18%
	(3.49 to 4.37)	(4.46 to 5.4)	(0.84 to 1.33)	(0.75 to 0.93)	(1.64 to 2.03)	(2.58 to 3.62)	(12.35 to 14.9)	(25.24 to 30.33)	(2.35 to 4.01)
Southern Sub-Saharan Africa	4.36	3.96	−0.39%	1.75	1.88	0.06%	28.94	30.56	0.02%
	(3.91 to 4.8)	(3.55 to 4.36)	(−0.53 to −0.25)	(0.96 to 2.52)	(1.25 to 2.32)	(−0.13 to 0.24)	(15.72 to 40.97)	(20.09 to 36.79)	(−0.24 to 0.29)
Tropical Latin America	10.58	19.33	2.65%	0.71	0.81	0.92%	14.43	16.84	0.84%
	(9.59 to 11.53)	(17.4 to 21.2)	(2.34 to 2.95)	(0.64 to 0.76)	(0.69 to 0.91)	(0.79 to 1.06)	(13.42 to 15.34)	(15.03 to 18.78)	(0.69 to 0.98)
Western Europe	3.1	3.42	0.19%	0.73	0.33	−2.02%	10.16	4.78	−2.99%
	(2.81 to 3.44)	(3.09 to 3.79)	(−0.15 to 0.54)	(0.65 to 0.78)	(0.27 to 0.36)	(−2.39 to −1.64)	(9.29 to 10.73)	(4.14 to 5.2)	(−3.23 to −2.74)
Western Sub-Saharan Africa	0.85	0.86	0.11%	0.03	0.03	−0.27%	0.65	0.88	1.62%
	(0.72 to 0.99)	(0.73 to 0.99)	(0.04 to 0.17)	(0.01 to 0.05)	(0.01 to 0.07)	(−0.56 to 0.02)	(0.25 to 1.08)	(0.31 to 1.61)	(1.29 to 1.94)

At the region level (Table 1 and Supplementary Table S1), the most significant number of prevalent cases was observed in South Asia (55,449, 47,550 to 63,302) with an EPAC of 1.24% (0.99 to 1.49), and the highest ASR of prevalence was observed in high-income North America (30.82, 27.77 to 33.94). From 1990 to 2021, the total prevalence of cases increased in most GBD regions. However, the ASR of prevalence rose in 10 GBD regions and declined in 11 regions. Among them, Tropical Latin America showed the highest upward trend with an EPAC of 2.65% (2.34 to 2.95), while Andean Latin America exhibited the highest downward trend with an EPAC of −0.57% (−0.62 to −0.52).

**Figure 1:**
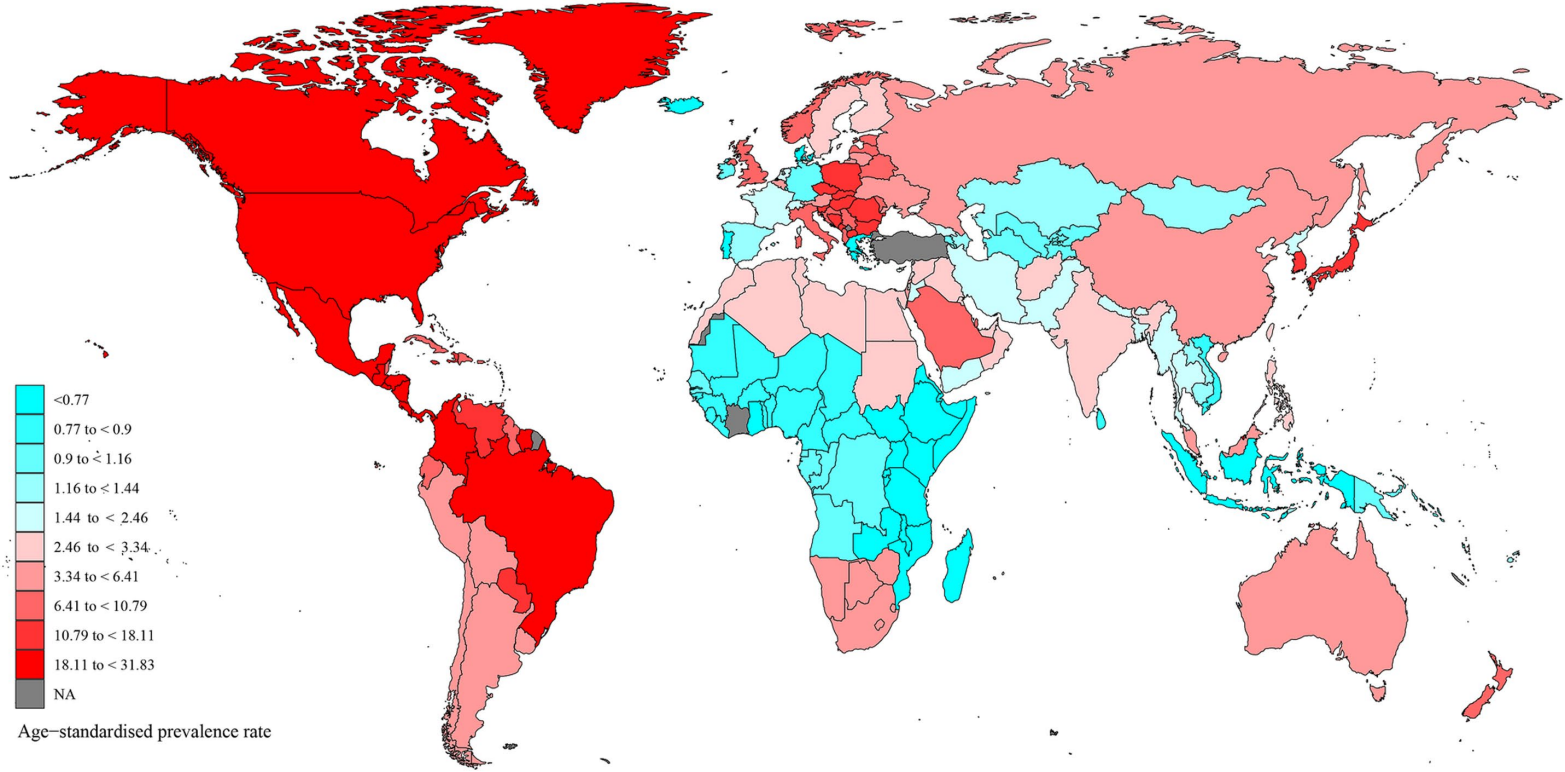
The global age-standardized prevalence rate.

At the national and territorial level in 2021 (Figure 1 and Supplementary Table S2), the United States of America (188,420, 168,469 to 207,804, EPAC = −0.03%, −0.14 to 0.09), China (102,939, 92,963 to 114,787, EPAC = 1.07% 0.85 to 1.29) and India (47,586, 40,841 to 54,289, EPAC = 1.50%, 1.21 to 1.79) were among the top three highest number of prevalence cases. In 2021, the highest ASR of prevalence was observed in the United States of America (31.83, 28.67 to 35.04), Barbados (28.58, 25.94 to 31.48), and Panama (24.7, 22.18 to 27.15). From 1990 to 2021, the ASR of prevalence increased in 96 and decreased in 106 countries (Supplementary Table S2). Among them, Brazil exhibited the highest upward trend with an EPAC of 2.96 (2.38 to 3), and Portugal exhibited the highest downward trend with an EPAC of −2.43 (−2.96 to −1.9).

### 3.2 Deaths related to decubitus ulcers

In 2021, there were 37,033 deaths (28,523 to 42,237) worldwide, which increased from 16,622 (13,738 to 19,753) in 1990 (Supplementary Table S1). However, the global ASR of death of decubitus ulcers slightly decreased from 0.53 (0.45 to 0.62) in 1990 to 0.46 (0.36 to 0.52) in 2021, with an EAPC of −0.58% (−0.71 to −0.46) (Table 1).

At the region level (Table 1 and Supplementary Table S1), South Asia had the highest number of deaths at 7005 (4,404 to 8,473), with an EAPC of 0.71% (0.51 to 0.91), and the highest ASR of death was observed in Southern Sub-Saharan Africa (1.88, 1.25 to 2.32). From 1990 to 2021, an increase in total death cases was observed in most GBD regions. However, the ASR of death increased in 10 GBD regions and decreased in 11 regions. Among them, Eastern Europe exhibited the highest upward trend with an EPAC of 4.79% (4.18 to 5.39), and the high-income Asia Pacific showed the highest downward trend with an EPAC of −3.14% (−3.67 to −2.61).

At the national and territorial level in 2021 (Supplementary Table S3), India (5,441, 2,999 to 7,232, EPAC = −1.51%, −2.33 to −0.68), China (3,132, 1,563 to 4,127, EPAC = 2.26%, 1.17 to 3.35) and Brazil (1936, 1,658 to 2,179, EPAC = 0.92%, 0.79 to 1.06) were among the top three highest number of death cases. In 2021, the highest ASR of death was observed in Barbados (12.41, 10.11 to 14.59), Grenada (8.14, 7.05 to 8.96), and Dominica (7.39, 5.63 to 9.41). From 1990 to 2021, the ASR of death increased in 117 and decreased in 85 countries. Among them, Georgia exhibited the highest upward trend with an EPAC of 14.32% (11.65 to 17.06), and Portugal exhibited the highest downward trend with an EPAC of −16.54% (−17.98 to −15.08).

### 3.3 DALYs of decubitus ulcers in 2021 and 1990

In 2021, decubitus ulcers account for 803,747 (612,264 to 903,723) DALY, 101927 (71,278 to 135,394) YLD and 701,821 (515,071 to 809,964) YLL (Supplementary Tables S1, S5, S6), which increased from 408,887 (329,847 to 490,564) DALY, 48905 (33,686 to 65,403) YLD and 359,982 (279,773 to 445,955) YLL in 1990. The global ASR of DALY decreased from 10.74 (8.87 to 12.74) in 1990 to 9.7 (7.41 to 10.88) in 2021, with an EAPC of −0.58% (−0.77 to −0.39). Similar downward trends were observed in the ASRs of YLD (EAPC = −0.04%, −0.12 to 0.04) and YLL (EAPC = −0.64%, −0.77 to −0.51).

At the regional level (Supplementary Tables S1, S5, S6), South Asia had the most significant number of DALY (162,008, 100,424 to 214,071) in 2021. The highest ASR of DALY in 2021 was observed in Eastern Sub-Saharan Africa at 34.69 (22.73 to 53.24). DALYs increased across most GBD regions from 1990 to 2021. The ASR of DALY declined in 10 regions, with the most significant decrease in Australasia (EAPC = −3.35%, −3.95 to −2.74), while Southern Latin America exhibited the most significant increase (EAPC = 3.18%, 2.35 to 4.01).

At the national and territorial level (Supplementary Tables S4, S7, S8), India (146,565, 90,927 to 191,410), China (61,934, 36,313 to 77,402), and the United States of America (50,615, 41,961 to 59,890) were still among the top 3 highest number of DALY. The highest ASR of DALY was observed in Barbados in 2021 (196.36, 160.49 to 235.17). From 1990 to 2021, 100 countries or territories experienced an increase in the ASR of DALY, and the most significant increase was found in Georgia (EAPC = 9.16%, 7.50 to 10.84). A total of 102 countries or territories experienced a decrease in the ASR of DALY, and the most significant reduction was found in France (EAPC = −5.76%, −6.31 to −5.20).

### 3.4 Burden of decubitus ulcers according to SDI

**Figure 2:**
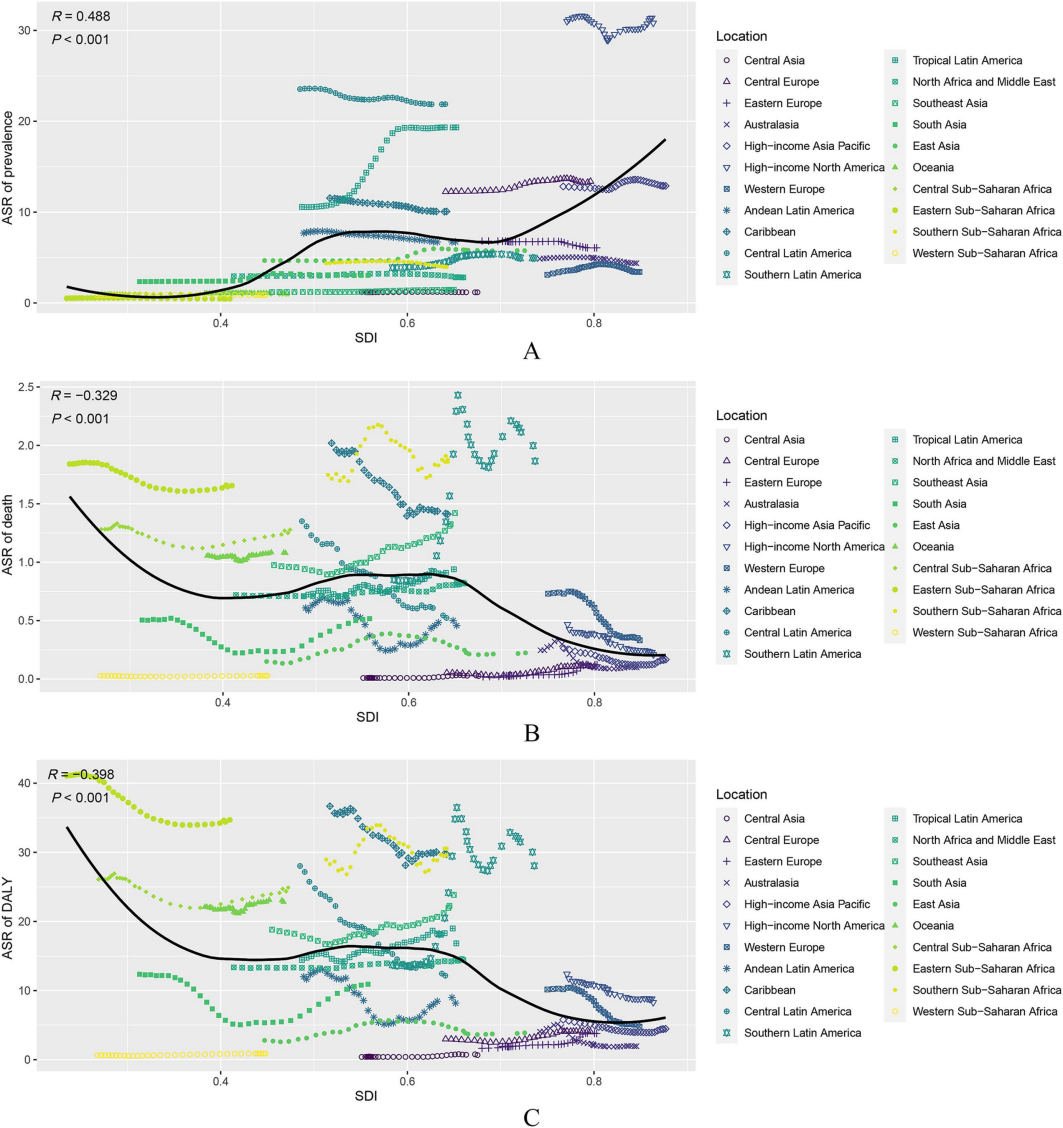
Association of age-standardized rate (ASR) of prevalence, death, and disability adjusted life-year (DALY) due to decubitus ulcers and SDI for 21 regions in the GBD study. **(A)** ASR of prevalence; **(B)** ASR of death; **(C)** ASR of DALY.

The global and regional ASRs of prevalence, death, DALY, YLD, and YLL in relation to SDI are shown in Figure 2 and Supplementary Figure 1. Higher SDI levels were associated with higher ASRs of prevalence (*R* = 0.488, *p* < 0.001, Figure 2A) and YLD (*R* = 0.495, *p* < 0.001, Supplementary Figure 1A). Conversely, higher SDI levels were associated with lower ASRs of death (*R* = -0.329, *p* < 0.001, Figure 2B), DALY (*R* = -0.398, *p* < 0.001, Figure 2C), and YLL (*R* = -0.445, *p* < 0.001, Supplementary Figure 1B). For example, the high-SDI quintile had the highest ASRs of prevalence (14.41, 13.04 to 15.83) and YLD (2.23, 1.55 to 2.96). Conversely, this quintile had the lowest ASRs of death (0.26, 0.22 to 0.28), DALY (6.66, 5.87 to 7.53), and YLL (4.43, 3.96 to 4.88). Between 1990 and 2021, the ASR of prevalence increased in all SDI quintiles except the high-SDI quintile (ESPA = -0.18%, −0.23 to −0.13). Moreover, the ASRs of death and DALY were decreased in all SDI quintiles except the high-middle SDI (ESPA = 2.09%, 1.75 to 2.44, and ESPA = 1.59%, 1.19 to 1.99, respectively). At the country level, similar trends were observed in ASRs of prevalence (*R* = 0.318, *p* < 0.001, Supplementary Figure 2A) and YLD (*R* = 0.325, *p* < 0.001, Supplementary Figure 2D). However, no significant correlations were found among the ASRs of death (*R* = -0.026, *p* = 0.711, Supplementary Figure 2B), DALY (*R* = -0.08, *p* = 0.26, Supplementary Figure 2C), and YLL (*R* = -0.092, *p* = 0.191, Supplementary Figure 2E).

### 3.5 Burden of decubitus ulcers according to age

**Figure 3:**
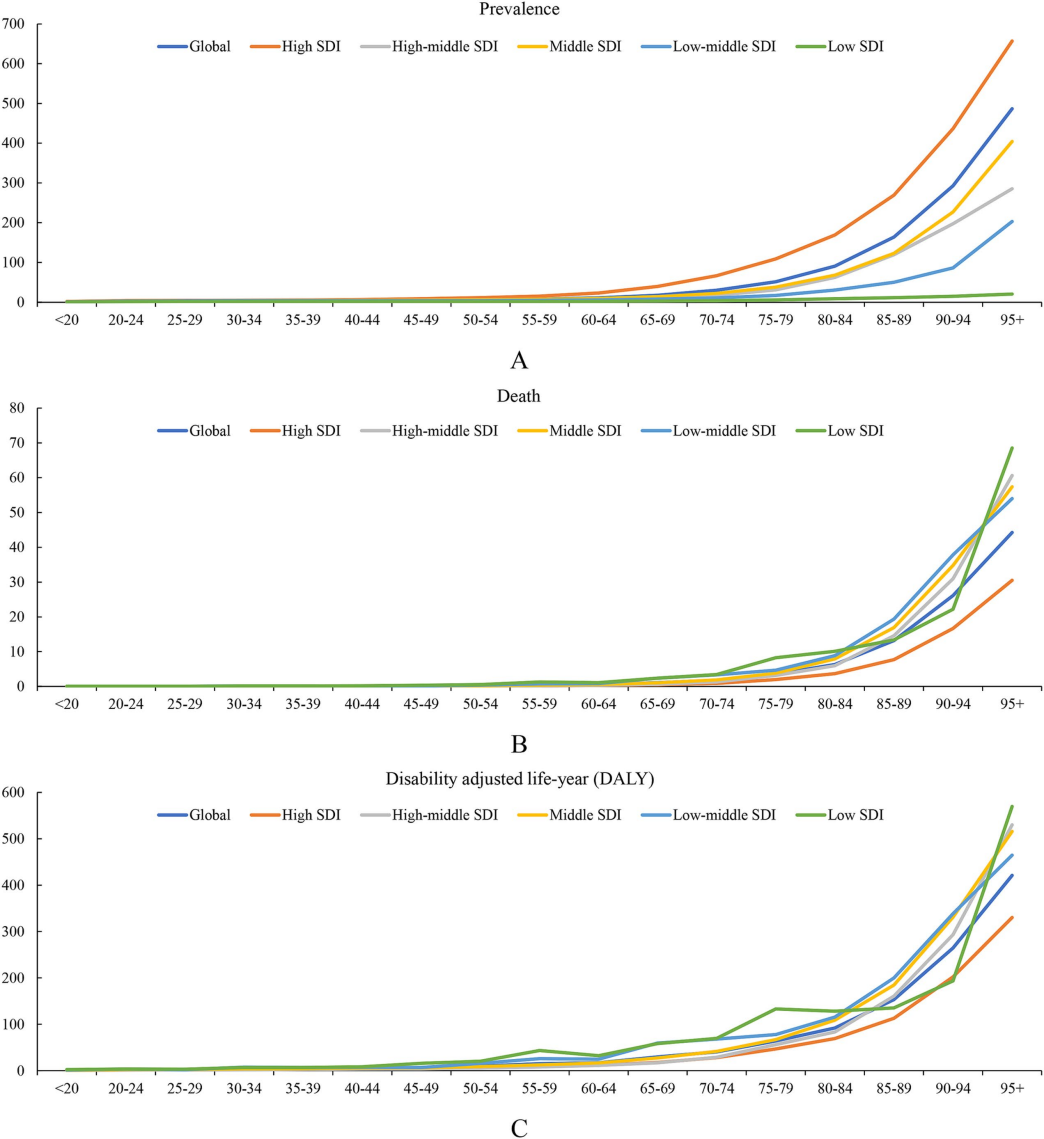
Association of age-standardized rate (ASR) of prevalence, death, and disability adjusted life-year (DALY) due to decubitus ulcers and age in the GBD study. **(A)** ASR of prevalence; **(B)** ASR of death; **(C)** ASR of DALY.

The burden of decubitus ulcers varies significantly across different age groups. Globally, the ASR of prevalence increased sharply with age (Figure 3A). Younger age groups, particularly those under 20, exhibit very low prevalence rates, with a global ASR of prevalence of 0.79 (0.56 to 1.06). However, as age increases, the ASR of prevalence rose sharply, peaking in the oldest age groups. In individuals aged 60–64, the ASR of prevalence was 11.08 (8.05 to 14.46), and it reached its highest at 486.52 (377.03 to 621.37) in the 95 years above group. This trend was consistent across all SDI levels, with higher SDI regions generally exhibiting higher ASRs of prevalence across all age groups compared to lower SDI regions.

The analysis of ASR of death indicated a significant increase with advancing age across all SDI categories, with a stark acceleration in older adults (Figure 3B). Globally, ASR of death remained relatively low in individuals under 50, but they started to rise significantly in the 50–54 age group, with an ASR of death of 0.23 (0.23, 0.16 to 0.28). This trend intensified dramatically in older age groups. For instance, individuals aged 80–84 have an ASR of death of 6.33 (6.33, 4.71 to 7.21), and those aged 95 and above face an alarming rate of 44.26 (44.26, 33.1 to 51.72). Higher SDI levels were associated with lower ASRs for death, with low-middle and low-SDI regions showing the highest ASRs across all age groups.

The ASR of DALY followed a similar upward trend with age (Figure 3C). Globally, the ASR of DALY rose significantly after the age of 60–64 (16.92, 12.51 to 20.65), peaking at 420.86 (322.25 to 485.2) in the 95+ age group. This trend reflected the increasing burden of disease and disability as people age, with the most significant increase observed in the older adult population. Higher SDI levels were associated with lower ASR of DALY than the global average. This lower trend continued across age groups, with ASR of DALY reaching 330.16 (330.16, 257.87 to 383.55) for those aged 95 and above, compared to the global average of 420.86 (420.86, 322.25 to 485.2). However, low-SDI regions experienced the highest DALYs across all age groups. For individuals under 20, the ASR of DALY was 2.25 (2.25, 1.22 to 3.33), which was significantly higher than the global average. This trend continued with age, with ASR of DALY reaching 569.48 (569.48, 355.15 to 810.33) for the 95 and above age group, far exceeding the global average of 420.86 (420.86, 322.25 to 485.2).

### 3.6 Burden of decubitus ulcers during the COVID-19 pandemic

To better understand the impact of the COVID-19 pandemic on the global burden of pressure ulcers, the data from 2019–2021 were analyzed separately. The ESPA of ASRs of prevalence, death, and DALY from 1990 to 2021, 1990 to 2019, and 2019 to 2021 is summarized in Supplementary Tables S9, S10. At the global level, the positive part was that during the pandemic period, the ASR of prevalence exhibited a decreasing trend (−0.49%, −0.93 to −0.06), which was better than the overall trend (−0.02%, −0.1 to 0.06) and pre-pandemic period (0.00%, −0.09 to 0.09). Similar trends were observed in the ASR of YLD. However, the declining trend of the ASR of death slowed down during the pandemic (−0.26%, −0.78 to 0.27), which was worse than the overall trend (−0.58%, −0.71 to −0.46) and pre-pandemic period (−0.63%, −0.76 to −0.5). Similar trends were observed in the ASRs of DALY and YLL.

At the SDI quintile level, the trends of ASRs of prevalence and YLD generally followed the global trend. However, it is worth noticing that the ASR of death exhibited an increasing trend during the pandemic in the middle SDI (0.12%, 0.09 to 0.16), although it kept a decreasing trend in the overall period (−0.18%, −0.24 to −0.12) and pre-pandemic period (−0.21%, −0.27 to −0.15). ASRs of death among low-middle SDI (0.48%, −1.2 to 2.2) and low SDI (0.19%, −0.4 to 0.78) exhibited an increasing trend with no statistical significance, even though they kept a decreasing trend before the pandemic (−0.46%, −0.75 to −0.17 and − 0.58%, −0.72 to −0.44 respectively). The trend was not optimistic in the high-SDI quintile. The decreasing trend became not statistically significant during the pandemic (−2.02%, −4.15 to 0.17). In addition, the high-middle SDI quintiles reversed the increasing trend during the pandemic (−1.03%, −2 to −0.06). For DALY, the middle and low SDI quintiles experienced a downward trend before the pandemic. However, during the pandemic, this trend reversed, and both quintiles saw an upward trend (0.94%, 0.82 to 1.05, and 0.38%, 0.05 to 0.71, respectively). A similar pattern was observed for YLL, where the middle (1.06%, 0.93 to 1.2) and low (0.39%, 0.05 to 0.73) SDI quintiles also shifted from a decreasing trend to an increasing trend during the pandemic.

## 4 Discussion

To the best of our knowledge, this is the first study to report the prevalence, death, DALY, YLL, and YLD of decubitus ulcers from 1990 to 2021 at the global, regional, and national levels. The findings underscore the significant health burden posed by decubitus ulcers, particularly in aging populations and regions with lower SDI.

From 1990 to 2021, an increased trend was observed in the number of decubitus ulcers-related prevalence, death, DALY, YLL, and YLD, indicating a growing health burden. However, their corresponding ASRs decreased globally and among most regions and nations. The results showed that while the absolute number of cases was rising due to population growth and aging (12), the relative risk per capita may be stabilizing or improving slightly. This trend also indicated advancements in healthcare practices, increased awareness, and possibly better preventive measures being implemented globally.

Previous research has linked decubitus ulcers to chronic diseases and inadequate healthcare (13, 14). That means regions with lower SDI might be affected more by decubitus ulcers. However, an interesting finding of the study was that a higher SDI was associated with higher ASRs of prevalence and YLD, with North America, particularly the United States, showing the highest rates. The reason could be explained as follows. Firstly, higher SDI regions often have more advanced healthcare systems and better diagnostic capabilities (15). This led to more accurate detection and reporting of decubitus ulcers. In contrast, lower SDI regions might underreport these cases due to limited healthcare infrastructure, lack of access to medical care, and insufficient record-keeping practices (16, 17). Secondly, higher SDI regions typically have a higher life expectancy (18), resulting in a larger older adult population who are at greater risk for chronic diseases and conditions like decubitus ulcers (19). Thirdly, smoking is a risk factor for decubitus ulcers (20). Our findings indicated that lower SDI regions exhibit a lower burden of decubitus ulcers, which may partially be attributed to lower smoking rates in these areas (11, 21). However, recent global tobacco usage trends indicate a shift in smoking prevalence, with a decline in high-SDI regions and a concerning rise among younger populations in certain low-and middle-SDI countries (21, 22). If tobacco consumption continues to increase among younger individuals in these regions, this could contribute to a future rise in the burden of decubitus ulcers, as prolonged smoking exposure may exacerbate risk factors such as poor wound healing and vascular impairment (23). Fourthly, immigration trends and the increasing movement of individuals from developing to developed countries may influence the GBD estimates. Migrants from lower SDI regions often face barriers in accessing healthcare, which can delay diagnosis and treatment, leading to an underestimation of the disease burden in these populations (24). Additionally, new immigrants frequently encounter health disparities due to language barriers, cultural differences, and reluctance to participate in health-promoting activities (25, 26). Limited access to preventive care and rehabilitation services may further increase their risk of developing decubitus ulcers. Despite higher ASRs of prevalence and YLD in high-SDI regions, these areas exhibited lower ASRs of death, DALY, and YLL. This may be attributed to better healthcare infrastructure, early interventions, and continuous care, which reduce complications and improve the management of decubitus ulcers. While the prevalence of non-fatal conditions remains high, higher quality healthcare likely mitigates life-threatening outcomes.

The marked increase in ASRs of prevalence, death, and DALY with advancing age underscored the vulnerability of older populations to decubitus ulcers. This trend could be explained as follows. Firstly, as individuals age, physiological changes such as thinning skin, reduced elasticity, and decreased mobility elevate the risk of developing decubitus ulcers (27). Additionally, the higher prevalence of chronic conditions like diabetes, cardiovascular diseases, and dementia in older populations impairs circulation and sensation (28). These factors collectively increase their susceptibility to decubitus ulcers, contributing to higher rates of prevalence, complications, and mortality. Secondly, older adults often experience decreased mobility and sensory perception, both of which are critical for preventing decubitus ulcers. Reduced mobility limits their ability to change positions regularly, while impaired sensory perception may prevent them from feeling discomfort or pain caused by prolonged pressure (29). This lack of movement and sensation exacerbates the risk of skin breakdown and ulcer development, leading to increased prevalence and severity of decubitus ulcers. Thirdly, across all SDI levels, older populations may face challenges in accessing appropriate healthcare and caregiving (30). Inadequate training for caregivers, insufficient healthcare resources, and lack of awareness may lead to higher rates of these conditions (31). Effective, age-specific healthcare strategies are essential to address these gaps, ensuring that older adults receive comprehensive care tailored to their unique needs, thereby reducing the prevalence and impact of decubitus ulcers.

During the COVID-19 period, the burden of decubitus ulcers showed notable changes globally. The ASRs of prevalence and YLD decreased compared to the overall and pre-pandemic trends. However, the ASRs of death and DALY exhibited a slower decline or even an increase in some regions and SDI quintiles, highlighting the strain on healthcare systems during the pandemic. These observed changes may be linked to COVID-19, as suggested by multiple lines of evidence. Firstly, the pandemic overwhelmed healthcare systems, diverting resources and attention away from routine care and chronic condition management (32), including the prevention and treatment of decubitus ulcers. In addition, aging individuals and people with different diseases are more vulnerable to COVID-19 (33). This led to an increase in the ASRs of death and DALY, particularly in regions with less resilient healthcare infrastructures. Secondly, lockdowns and reduced mobility during the pandemic disrupted regular healthcare services and preventive measures for decubitus ulcers. Patients, especially those in care facilities and older adult populations, may have experienced reduced monitoring and care, contributing to the worsening trends in mortality and disability (34). Thirdly, the pandemic exacerbated existing socio-economic disparities, with low and middle SDI regions experiencing increased ASRs of death and DALY. These areas often lacked the resources to cope with the additional healthcare burdens posed by COVID-19, leading to poorer outcomes for conditions like decubitus ulcers that require consistent and comprehensive care (35).

In this study, we utilized ASRs to facilitate cross-population comparisons while accounting for differences in age structures. This approach provides valuable insights but also has certain limitations that warrant discussion. On the positive side, ASRs allow for a more accurate comparison of decubitus ulcer burden across regions with varying demographic compositions. By adjusting for age distribution, ASRs help highlight regions where the burden is disproportionately high relative to the expected population structure (36). However, relying solely on ASRs may overlook key aspects of disease burden, particularly in lower SDI regions and younger populations (37). In lower SDI countries, where the overall population is younger, the absolute number of decubitus ulcer cases may still be substantial despite lower ASRs. The high incidence among younger individuals—particularly those with malnutrition, trauma-related immobility, or neurological disorders—could be underestimated (38). Furthermore, ASRs may not fully capture the socioeconomic and healthcare disparities that influence access to treatment and prevention, leading to an underestimation of the actual burden in resource-limited settings.

While this study provides insights into the global burden of decubitus ulcers up until 2021, it is essential to note that the field has seen significant advancements in recent years. Improved risk assessment tools, such as machine learning-based predictive models, have enhanced early detection and personalized intervention strategies (39). Additionally, the development of advanced wound care technologies, including hydrogel dressings (40) and negative pressure wound therapy (41), has significantly improved healing outcomes. The integration of sensors and telemedicine in wound management has also facilitated timely interventions, particularly in remote and underserved areas (42, 43). Moreover, increased awareness and updated clinical guidelines have strengthened multidisciplinary approaches, emphasizing early mobility programs, nutritional support, and pressure relief strategies (44, 45). These innovations collectively contribute to reducing the burden of decubitus ulcers, particularly in high-risk populations.

This studies also have some limitations. Firstly, the study relies on the GBD 2021 database, which might have inconsistencies or gaps in data reporting across different countries and regions. Secondly, the study does not differentiate between the various stages of decubitus ulcers (Stage 1 to Stage 4), which can vary significantly in terms of clinical management and outcomes. Thirdly, the analysis divided data into pre-COVID-19 and COVID-19 eras, which might not fully capture the long-term impacts of the pandemic on the burden of decubitus ulcers. Despite these limitations, the study’s strength lies in its comprehensive and up-to-date analysis of the global burden of decubitus ulcers. More granular, extensive data and analyses are still needed to understand and address the global burden of decubitus ulcers.

## 5 Conclusion

The study provides comprehensive insights into the epidemiology of decubitus ulcers, highlighting global increases in prevalence, mortality, and disability burden over three decades. Regional disparities in healthcare access and outcomes underscore the need for targeted interventions to reduce the burden of decubitus ulcers, particularly in resource-constrained settings. Future research should focus on addressing these disparities and improving healthcare delivery to mitigate the impact of decubitus ulcers globally.

## Data Availability

The original contributions presented in the study are included in the article/Supplementary material, further inquiries can be directed to the corresponding authors.

## Footnotes

^1^ http://ghdx.healthdata.org/gbd-results-tool
