# Supplementary material for: Global, regional and national burden of decubitus ulcers in 204 countries and territories from 1990 to 2021: a systematic analysis based on the global burden of disease study 2021
Source: Front Public Health. 2025 Feb 26;13:1494229. doi: 10.3389/fpubh.2025.1494229 (PMC11897561; doi:10.3389/fpubh.2025.1494229)
Supplement: Supplementary file 1 [file Data_Sheet_1.DOCX]

Supplementary Material

# Supplementary Figures and Tables


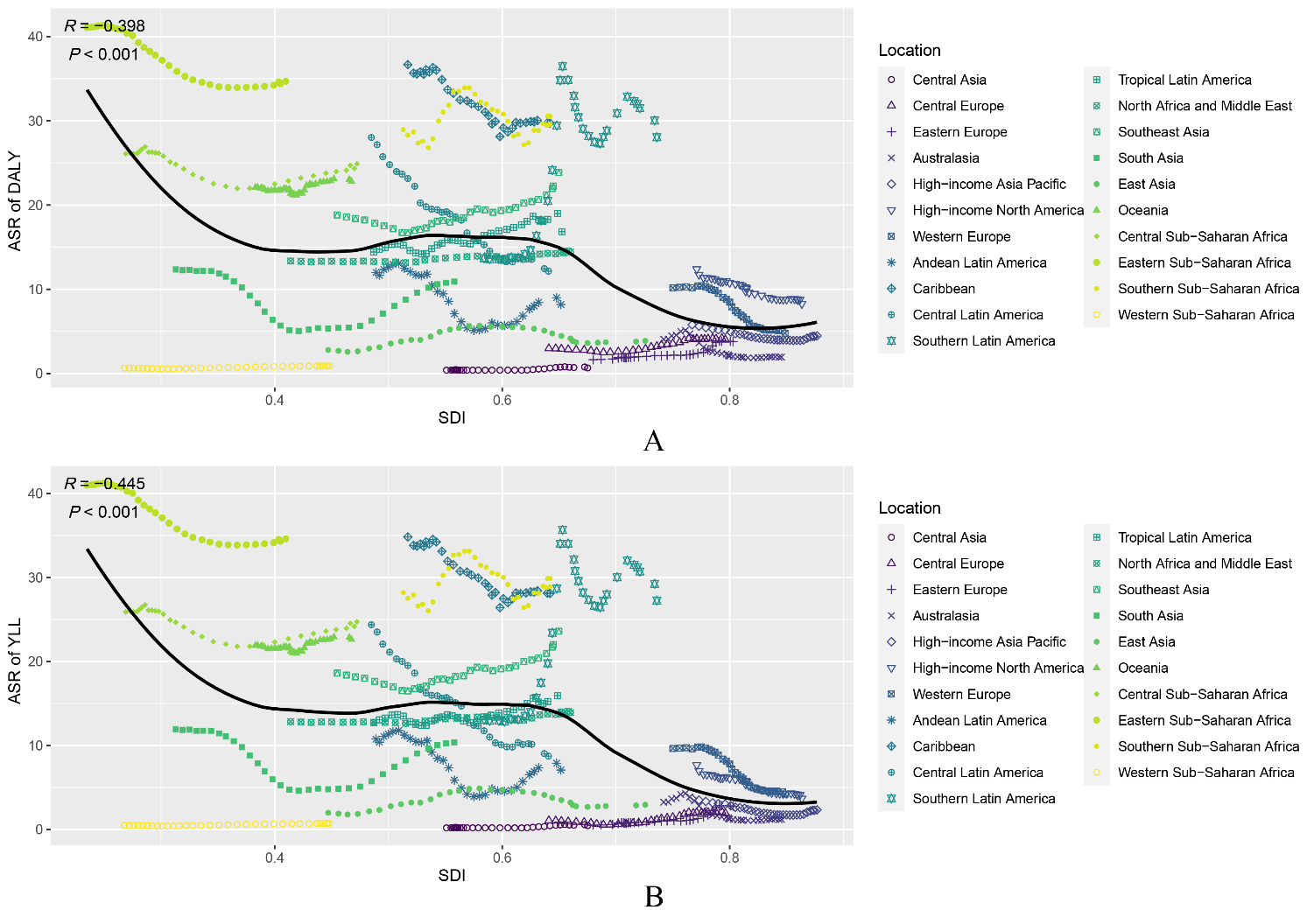
**Supplementary Figure 1**: Association of age-standardized rate (ASR) of years lived with disability (YLD) and years of life lost (YLL) due to decubitus ulcers and SDI for 21 regions in the GBD study. A. ASR of YLD; B. ASR of YLL.


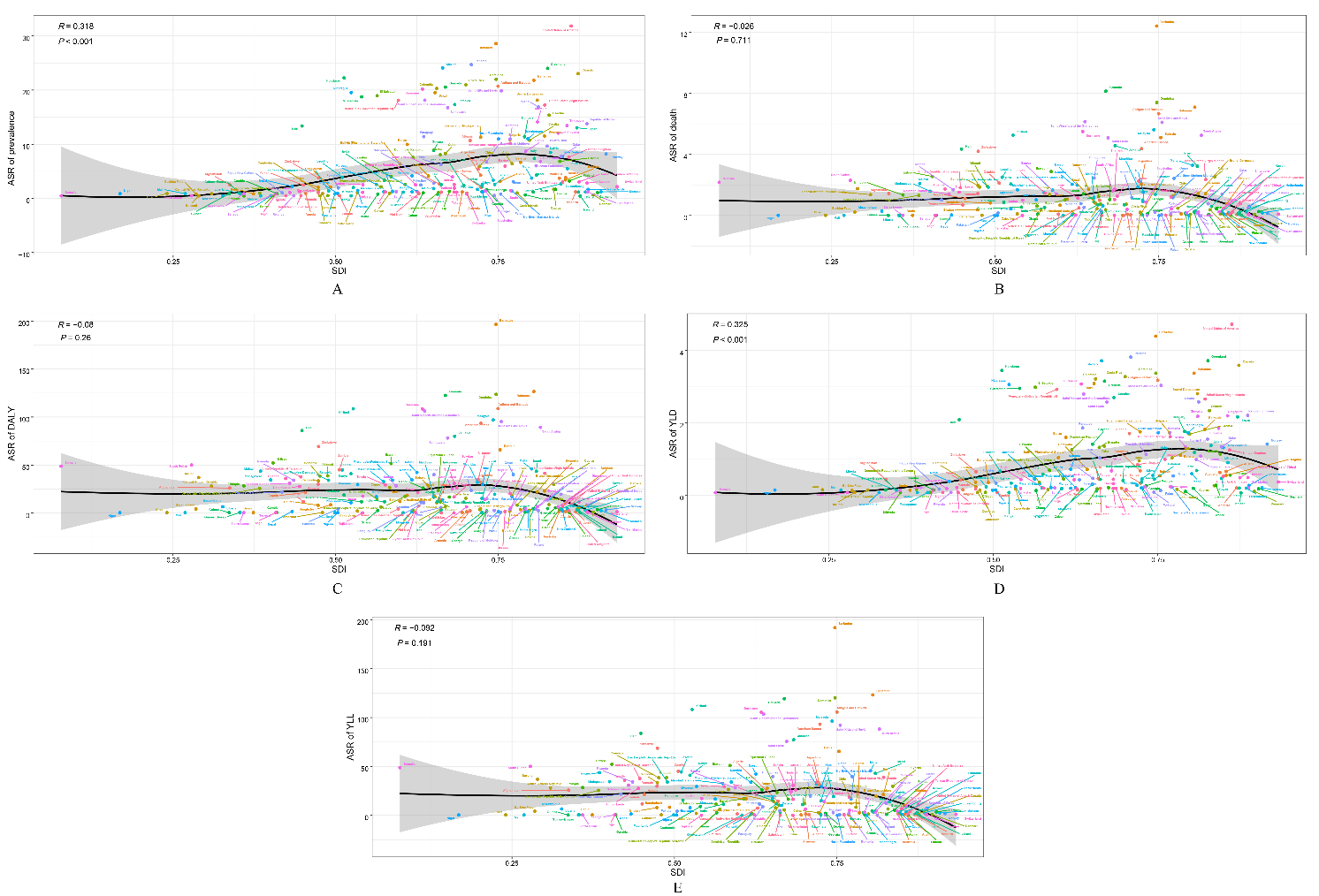
**Supplementary Figure 2**: Association of age-standardized rate of prevalence, death, Disability adjusted life-year (DALY), years lived with disability (YLD) and years of life lost (YLL) due to decubitus ulcers and SDI for 204 countries and territories.

| Table S1: Prevalence, death and disability-adjusted life years (DALY) counts for decubitus ulcers in 1990 and 2021 at the global, SDI quintile and GBD region levels. | | | | | | | | |
| --- | --- | --- | --- | --- | --- | --- | --- | --- |
| Location | Prevalence counts | |  | Death counts | |  | DALY counts | |
|  | 1990 (95% UI) | 2021 (95% UI) |  | 1990 (95% UI) | 2021 (95% UI) |  | 1990 (95% UI) | 2021 (95% UI) |
| Global | 300442 | 645588 |  | 16622 | 37033 |  | 408887 | 803747 |
|  | (270738 to 333579) | (582432 to 712876) |  | (13738 to 19753) | (28523 to 42237) |  | (329847 to 490564) | (612264 to 903723) |
| SDI quintile |  |  |  |  |  |  |  |  |
| High SDI | 158248 | 304776 |  | 6301 | 6471 |  | 118418 | 139361 |
|  | (140958 to 177683) | (273485 to 336885) |  | (5644 to 6717) | (5423 to 7071) |  | (108945 to 129112) | (123393 to 157430) |
| High-middle SDI | 51360 | 100827 |  | 1755 | 7764 |  | 39480 | 133709 |
|  | (45749 to 57841) | (90594 to 112014) |  | (1574 to 2059) | (6165 to 8671) |  | (35333 to 45320) | (107392 to 149671) |
| Middle SDI | 61883 | 163980 |  | 3717 | 11377 |  | 100750 | 237465 |
|  | (55094 to 69123) | (148650 to 180538) |  | (2968 to 4635) | (8325 to 13352) |  | (80363 to 123171) | (173991 to 269346) |
| Low-middle SDI | 23764 | 63031 |  | 3159 | 8098 |  | 96732 | 196156 |
|  | (20539 to 26812) | (55741 to 70473) |  | (1894 to 4272) | (5287 to 10113) |  | (60096 to 127791) | (129297 to 242117) |
| Low SDI | 4806 | 12308 |  | 1665 | 3271 |  | 52922 | 95958 |
|  | (4034 to 5574) | (10451 to 14213) |  | (1007 to 2655) | (2092 to 4407) |  | (30688 to 80594) | (58607 to 130148) |
| GBD region |  |  |  |  |  |  |  |  |
| Andean Latin America | 1703 | 4071 |  | 109 | 253 |  | 2687 | 4755 |
|  | (1529 to 1887) | (3651 to 4506) |  | (79 to 135) | (198 to 347) |  | (1902 to 3320) | (3766 to 6222) |
| Australasia | 1082 | 2093 |  | 51 | 66 |  | 899 | 1102 |
|  | (969 to 1216) | (1863 to 2357) |  | (45 to 57) | (53 to 76) |  | (808 to 985) | (935 to 1263) |
| Caribbean | 2983 | 5396 |  | 448 | 775 |  | 9407 | 15687 |
|  | (2708 to 3297) | (4900 to 5927) |  | (398 to 517) | (657 to 934) |  | (7968 to 11355) | (12896 to 19334) |
| Central Asia | 550 | 861 |  | 4 | 17 |  | 192 | 505 |
|  | (475 to 636) | (745 to 994) |  | (3 to 5) | (14 to 21) |  | (145 to 252) | (419 to 606) |
| Central Europe | 15770 | 28979 |  | 71 | 246 |  | 4010 | 8531 |
|  | (13978 to 18094) | (25985 to 32343) |  | (63 to 80) | (211 to 276) |  | (3220 to 4957) | (7041 to 9967) |
| Central Latin America | 21189 | 53435 |  | 937 | 1224 |  | 24680 | 29689 |
|  | (19128 to 23318) | (48212 to 58882) |  | (893 to 974) | (1011 to 1509) |  | (23229 to 26138) | (24943 to 35831) |
| Central Sub-Saharan Africa | 324 | 775 |  | 210 | 506 |  | 6085 | 13971 |
|  | (266 to 385) | (628 to 931) |  | (67 to 326) | (204 to 792) |  | (1906 to 9631) | (5086 to 22087) |
| East Asia | 43575 | 104819 |  | 580 | 3575 |  | 19107 | 68753 |
|  | (38156 to 49244) | (94630 to 116921) |  | (449 to 1057) | (1978 to 4569) |  | (14772 to 28995) | (42787 to 84182) |
| Eastern Europe | 16675 | 18512 |  | 51 | 416 |  | 4156 | 11742 |
|  | (14729 to 19004) | (16454 to 21073) |  | (45 to 57) | (383 to 449) |  | (3207 to 5241) | (10642 to 13112) |
| Eastern Sub-Saharan Africa | 632 | 1371 |  | 1185 | 2363 |  | 36772 | 69406 |
|  | (481 to 796) | (1026 to 1740) |  | (687 to 2246) | (1543 to 3622) |  | (20945 to 65925) | (42325 to 106586) |
| High-income Asia Pacific | 23287 | 52638 |  | 412 | 1160 |  | 10309 | 21934 |
|  | (20831 to 25959) | (47164 to 59022) |  | (351 to 458) | (913 to 1340) |  | (8709 to 12018) | (18768 to 25475) |
| High-income North America | 110718 | 204537 |  | 1731 | 1484 |  | 44413 | 54047 |
|  | (97593 to 124747) | (182497 to 225362) |  | (1538 to 1841) | (1247 to 1699) |  | (39150 to 50524) | (44672 to 63885) |
| North Africa and Middle East | 6493 | 14910 |  | 899 | 2836 |  | 22355 | 62172 |
|  | (5554 to 7354) | (12861 to 16858) |  | (649 to 1335) | (2235 to 3399) |  | (16396 to 31147) | (49917 to 73717) |
| Oceania | 31 | 78 |  | 23 | 63 |  | 683 | 1837 |
|  | (25 to 37) | (67 to 91) |  | (9 to 52) | (23 to 125) |  | (270 to 1555) | (621 to 3785) |
| South Asia | 20722 | 55449 |  | 2521 | 6232 |  | 87407 | 162008 |
|  | (17518 to 23867) | (47550 to 63302) |  | (1434 to 3693) | (3530 to 8376) |  | (54522 to 123268) | (100424 to 214071) |
| Southeast Asia | 3181 | 8579 |  | 1930 | 7005 |  | 49293 | 137942 |
|  | (2752 to 3667) | (7667 to 9558) |  | (1327 to 2883) | (4404 to 8473) |  | (32634 to 71881) | (85142 to 168445) |
| Southern Latin America | 1684 | 4376 |  | 343 | 1729 |  | 5935 | 25328 |
|  | (1505 to 1894) | (3944 to 4785) |  | (306 to 377) | (1517 to 1883) |  | (5372 to 6478) | (22733 to 27413) |
| Southern Sub-Saharan Africa | 1622 | 2617 |  | 363 | 779 |  | 7461 | 15462 |
|  | (1414 to 1815) | (2316 to 2928) |  | (200 to 516) | (517 to 952) |  | (3992 to 10382) | (9815 to 18909) |
| Tropical Latin America | 10185 | 47703 |  | 506 | 1964 |  | 13100 | 41871 |
|  | (9244 to 11208) | (43029 to 52515) |  | (466 to 536) | (1684 to 2210) |  | (12263 to 13924) | (37451 to 46662) |
| Western Europe | 16926 | 31702 |  | 4228 | 4284 |  | 59305 | 54445 |
|  | (15222 to 18922) | (28483 to 35338) |  | (3778 to 4498) | (3476 to 4727) |  | (54351 to 62699) | (46296 to 59471) |
| Western Sub-Saharan Africa | 1113 | 2687 |  | 19 | 56 |  | 632 | 2561 |
|  | (899 to 1335) | (2159 to 3246) |  | (4 to 35) | (11 to 113) |  | (291 to 1010) | (887 to 3987) |

| Table S2: Prevalence of decubitus ulcers in 1990 and 2021 and their temporal trends from 1990 to 2021 at the national and territorial levels. | | | | | |
| --- | --- | --- | --- | --- | --- |
| Location | Counts 1990  (95% UI) | Age-standardized prevalence rate  (95% UI) | Counts 2021 (95% UI) | Age-standardized prevalence rate  (95% UI) | EAPC (95% CI) |
| Afghanistan | 179 | 2.59 | 449 | 2.47 | -0.13% |
|  | (153 to 204) | (2.28 to 2.91) | (376 to 520) | (2.17 to 2.77) | (-0.19 to -0.07) |
| Albania | 228 | 11.38 | 419 | 10.68 | -0.21% |
|  | (204 to 256) | (10.02 to 13.01) | (365 to 485) | (9.48 to 12.26) | (-0.28 to -0.15) |
| Algeria | 443 | 2.79 | 1031 | 2.66 | -0.12% |
|  | (375 to 506) | (2.46 to 3.13) | (892 to 1171) | (2.34 to 2.97) | (-0.16 to -0.08) |
| American Samoa | 0 | 1.79 | 1 | 2.01 | 0.35% |
|  | (0 to 0) | (1.6 to 2.01) | (1 to 1) | (1.82 to 2.22) | (0.17 to 0.53) |
| Andorra | 1 | 1.32 | 2 | 1.19 | -0.35% |
|  | (1 to 1) | (1.15 to 1.53) | (2 to 2) | (1.04 to 1.38) | (-0.41 to -0.29) |
| Angola | 57 | 0.96 | 172 | 0.9 | -0.21% |
|  | (46 to 69) | (0.83 to 1.1) | (139 to 210) | (0.77 to 1.03) | (-0.26 to -0.16) |
| Antigua and Barbuda | 10 | 17.21 | 19 | 20.72 | 0.58% |
|  | (9 to 11) | (15.46 to 19.02) | (18 to 21) | (18.84 to 22.49) | (0.53 to 0.64) |
| Argentina | 1000 | 3.35 | 2463 | 4.27 | 1.19% |
|  | (888 to 1147) | (2.97 to 3.83) | (2198 to 2727) | (3.82 to 4.72) | (0.93 to 1.45) |
| Armenia | 23 | 0.94 | 39 | 0.96 | 0.19% |
|  | (20 to 28) | (0.81 to 1.09) | (34 to 45) | (0.84 to 1.1) | (0.12 to 0.25) |
| Australia | 818 | 4.4 | 1512 | 3.72 | -0.60% |
|  | (728 to 924) | (3.92 to 4.94) | (1334 to 1713) | (3.29 to 4.18) | (-0.63 to -0.58) |
| Austria | 597 | 5.52 | 612 | 3.83 | -1.58% |
|  | (541 to 670) | (5.01 to 6.11) | (547 to 687) | (3.42 to 4.35) | (-2.11 to -1.04) |
| Azerbaijan | 56 | 1.17 | 102 | 1.17 | 0.05% |
|  | (48 to 66) | (1.02 to 1.36) | (88 to 119) | (1.01 to 1.34) | (0.01 to 0.09) |
| Bahamas | 31 | 20.42 | 79 | 21.81 | 0.16% |
|  | (28 to 34) | (18.34 to 22.52) | (72 to 86) | (19.84 to 23.79) | (0.09 to 0.23) |
| Bahrain | 11 | 2.86 | 42 | 3.11 | 0.39% |
|  | (9 to 13) | (2.51 to 3.21) | (36 to 48) | (2.74 to 3.49) | (0.33 to 0.44) |
| Bangladesh | 1672 | 2.1 | 3269 | 2.03 | -0.11% |
|  | (1398 to 1958) | (1.79 to 2.41) | (2764 to 3763) | (1.74 to 2.33) | (-0.14 to -0.08) |
| Barbados | 84 | 27.64 | 142 | 28.58 | 0.02% |
|  | (75 to 93) | (25.04 to 30.32) | (128 to 157) | (25.94 to 31.48) | (-0.06 to 0.11) |
| Belarus | 825 | 6.96 | 937 | 6.66 | -0.12% |
|  | (728 to 932) | (6.17 to 7.82) | (825 to 1069) | (5.94 to 7.5) | (-0.17 to -0.08) |
| Belgium | 727 | 4.77 | 813 | 3.4 | -0.87% |
|  | (646 to 821) | (4.26 to 5.34) | (721 to 909) | (3.05 to 3.77) | (-1.56 to -0.16) |
| Belize | 12 | 10.66 | 30 | 9.96 | -0.33% |
|  | (10 to 13) | (9.56 to 11.81) | (27 to 33) | (8.93 to 11) | (-0.41 to -0.26) |
| Benin | 26 | 0.84 | 72 | 0.84 | 0.05% |
|  | (21 to 31) | (0.71 to 0.98) | (58 to 90) | (0.71 to 0.97) | (0 to 0.11) |
| Bermuda | 7 | 12.42 | 15 | 11.52 | -0.31% |
|  | (6 to 8) | (11.21 to 13.7) | (14 to 17) | (10.45 to 12.63) | (-0.34 to -0.29) |
| Bhutan | 10 | 2.1 | 16 | 2.05 | -0.06% |
|  | (8 to 12) | (1.81 to 2.41) | (14 to 19) | (1.74 to 2.36) | (-0.1 to -0.02) |
| Bolivia (Plurinational State of) | 228 | 6.62 | 565 | 6.36 | -0.13% |
|  | (201 to 255) | (5.91 to 7.39) | (501 to 634) | (5.62 to 7.12) | (-0.17 to -0.1) |
| Bosnia and Herzegovina | 382 | 10.98 | 643 | 11.31 | 0.16% |
|  | (339 to 434) | (9.69 to 12.57) | (566 to 730) | (10.11 to 12.64) | (0.09 to 0.23) |
| Botswana | 36 | 4.23 | 77 | 4.13 | 0.37% |
|  | (31 to 41) | (3.78 to 4.71) | (67 to 86) | (3.68 to 4.55) | (-0.3 to 1.03) |
| Brazil | 9906 | 10.6 | 47007 | 19.53 | 2.69% |
|  | (8997 to 10898) | (9.61 to 11.55) | (42405 to 51753) | (17.58 to 21.41) | (2.38 to 3) |
| Brunei Darussalam | 24 | 17.95 | 52 | 18.13 | 0% |
|  | (21 to 26) | (15.96 to 19.75) | (47 to 58) | (16.38 to 20.08) | (-0.02 to 0.03) |
| Bulgaria | 1097 | 11.5 | 1423 | 10.85 | -0.19% |
|  | (954 to 1285) | (10.09 to 13.21) | (1231 to 1674) | (9.62 to 12.4) | (-0.24 to -0.15) |
| Burkina Faso | 51 | 0.83 | 118 | 0.79 | -0.12% |
|  | (41 to 62) | (0.7 to 0.96) | (95 to 144) | (0.66 to 0.92) | (-0.17 to -0.06) |
| Burundi | 18 | 0.51 | 41 | 0.47 | -0.25% |
|  | (14 to 23) | (0.42 to 0.6) | (31 to 52) | (0.39 to 0.57) | (-0.31 to -0.19) |
| Cabo Verde | 3 | 0.93 | 5 | 1.05 | 0.37% |
|  | (2 to 3) | (0.8 to 1.07) | (4 to 6) | (0.92 to 1.21) | (0.32 to 0.41) |
| Cambodia | 54 | 1.19 | 153 | 1.4 | 0.56% |
|  | (46 to 62) | (1.06 to 1.36) | (135 to 173) | (1.27 to 1.57) | (0.53 to 0.58) |
| Cameroon | 57 | 0.84 | 177 | 0.87 | 0.15% |
|  | (46 to 69) | (0.71 to 0.98) | (141 to 216) | (0.74 to 1) | (0.11 to 0.2) |
| Canada | 8277 | 26.25 | 16099 | 23.03 | -0.47% |
|  | (7335 to 9379) | (23.32 to 29.71) | (14194 to 18439) | (20.54 to 26.25) | (-0.53 to -0.4) |
| Central African Republic | 15 | 0.97 | 29 | 0.86 | -0.42% |
|  | (12 to 18) | (0.83 to 1.11) | (23 to 35) | (0.74 to 0.98) | (-0.48 to -0.35) |
| Chad | 33 | 0.84 | 88 | 0.89 | 0.30% |
|  | (27 to 40) | (0.7 to 0.98) | (70 to 108) | (0.76 to 1.04) | (0.25 to 0.35) |
| Chile | 488 | 4.88 | 1581 | 6.38 | 1.10% |
|  | (437 to 544) | (4.37 to 5.45) | (1427 to 1735) | (5.79 to 6.99) | (0.83 to 1.37) |
| China | 42840 | 4.74 | 102939 | 5.89 | 1.07% |
|  | (37480 to 48388) | (4.23 to 5.29) | (92963 to 114787) | (5.33 to 6.5) | (0.85 to 1.29) |
| Colombia | 4860 | 26.19 | 11436 | 20.29 | -0.80% |
|  | (4410 to 5338) | (23.32 to 29.06) | (10320 to 12688) | (18.32 to 22.43) | (-0.86 to -0.75) |
| Comoros | 2 | 0.52 | 3 | 0.48 | -0.24% |
|  | (1 to 2) | (0.44 to 0.62) | (2 to 4) | (0.4 to 0.58) | (-0.29 to -0.19) |
| Congo | 15 | 1.03 | 34 | 0.93 | -0.36% |
|  | (12 to 17) | (0.9 to 1.18) | (27 to 41) | (0.81 to 1.07) | (-0.39 to -0.33) |
| Cook Islands | 0 | 0.94 | 0 | 0.99 | 0.28% |
|  | (0 to 0) | (0.82 to 1.08) | (0 to 0) | (0.88 to 1.13) | (0.24 to 0.32) |
| Costa Rica | 450 | 22.94 | 1158 | 20.98 | -0.34% |
|  | (406 to 497) | (20.55 to 25.3) | (1033 to 1288) | (18.78 to 23.23) | (-0.38 to -0.3) |
| Croatia | 573 | 10.91 | 929 | 10.75 | 0% |
|  | (507 to 666) | (9.63 to 12.61) | (813 to 1076) | (9.55 to 12.2) | (-0.05 to 0.04) |
| Cuba | 950 | 9.35 | 1493 | 8.27 | -0.40% |
|  | (851 to 1064) | (8.41 to 10.44) | (1336 to 1667) | (7.4 to 9.19) | (-0.48 to -0.33) |
| Cyprus | 12 | 1.86 | 34 | 1.66 | -0.27% |
|  | (11 to 14) | (1.63 to 2.12) | (29 to 38) | (1.48 to 1.89) | (-0.53 to -0.01) |
| Czechia | 1738 | 14.02 | 3232 | 15.37 | 0.61% |
|  | (1531 to 1987) | (12.47 to 15.75) | (2826 to 3679) | (13.59 to 17.33) | (0.43 to 0.79) |
| Democratic People's Republic of Korea | 308 | 2.18 | 599 | 2.12 | -0.10% |
|  | (266 to 350) | (1.93 to 2.47) | (533 to 680) | (1.91 to 2.4) | (-0.18 to -0.03) |
| Democratic Republic of the Congo | 227 | 1.09 | 518 | 0.96 | -0.43% |
|  | (185 to 270) | (0.95 to 1.24) | (422 to 624) | (0.83 to 1.09) | (-0.44 to -0.41) |
| Denmark | 58 | 0.72 | 74 | 0.64 | -0.64% |
|  | (50 to 68) | (0.62 to 0.85) | (64 to 86) | (0.55 to 0.74) | (-1.68 to 0.41) |
| Djibouti | 1 | 0.52 | 5 | 0.5 | -0.19% |
|  | (1 to 2) | (0.44 to 0.63) | (4 to 6) | (0.41 to 0.59) | (-0.24 to -0.14) |
| Dominica | 12 | 19.62 | 17 | 22 | 0.35% |
|  | (10 to 13) | (17.68 to 21.73) | (15 to 18) | (19.87 to 24.11) | (0.32 to 0.38) |
| Dominican Republic | 352 | 8.18 | 776 | 7.55 | -0.25% |
|  | (314 to 394) | (7.35 to 9.12) | (697 to 858) | (6.77 to 8.35) | (-0.34 to -0.16) |
| Ecuador | 376 | 6.14 | 1294 | 8.05 | 1.22% |
|  | (331 to 421) | (5.5 to 6.92) | (1171 to 1424) | (7.27 to 8.87) | (1.04 to 1.4) |
| Egypt | 1001 | 2.72 | 2068 | 2.57 | -0.17% |
|  | (851 to 1146) | (2.37 to 3.06) | (1764 to 2366) | (2.26 to 2.89) | (-0.23 to -0.11) |
| El Salvador | 663 | 19.1 | 1284 | 18.95 | 0.04% |
|  | (593 to 733) | (17.09 to 21.13) | (1155 to 1418) | (17.01 to 20.94) | (0 to 0.08) |
| Equatorial Guinea | 2 | 1 | 9 | 1.04 | 0.19% |
|  | (2 to 3) | (0.86 to 1.14) | (7 to 11) | (0.9 to 1.18) | (0.14 to 0.25) |
| Eritrea | 11 | 0.51 | 22 | 0.48 | -0.19% |
|  | (8 to 13) | (0.42 to 0.6) | (17 to 28) | (0.4 to 0.58) | (-0.25 to -0.12) |
| Estonia | 130 | 7.09 | 166 | 6.84 | -0.12% |
|  | (115 to 147) | (6.3 to 7.94) | (146 to 191) | (6.07 to 7.7) | (-0.16 to -0.07) |
| Eswatini | 20 | 4.05 | 33 | 4 | -0.03% |
|  | (17 to 23) | (3.62 to 4.49) | (28 to 37) | (3.57 to 4.43) | (-0.09 to 0.02) |
| Ethiopia | 172 | 0.52 | 355 | 0.46 | -0.42% |
|  | (131 to 216) | (0.43 to 0.62) | (265 to 454) | (0.37 to 0.55) | (-0.52 to -0.32) |
| Fiji | 4 | 1.18 | 8 | 1.29 | 0.21% |
|  | (3 to 5) | (1.05 to 1.34) | (7 to 9) | (1.15 to 1.43) | (0.18 to 0.23) |
| Finland | 224 | 3.37 | 348 | 3.21 | -0.34% |
|  | (197 to 259) | (2.99 to 3.85) | (303 to 405) | (2.82 to 3.67) | (-1.39 to 0.71) |
| France | 2430 | 2.74 | 2603 | 1.66 | -1.96% |
|  | (2148 to 2786) | (2.44 to 3.08) | (2295 to 2922) | (1.48 to 1.87) | (-2.09 to -1.84) |
| Gabon | 7 | 1.09 | 13 | 1 | -0.31% |
|  | (6 to 8) | (0.96 to 1.24) | (10 to 15) | (0.87 to 1.14) | (-0.34 to -0.29) |
| Gambia | 5 | 0.85 | 14 | 0.87 | 0.13% |
|  | (4 to 6) | (0.71 to 0.99) | (11 to 17) | (0.74 to 1.01) | (0.09 to 0.18) |
| Georgia | 84 | 1.49 | 93 | 1.57 | 0.31% |
|  | (73 to 97) | (1.3 to 1.71) | (82 to 105) | (1.4 to 1.78) | (0.23 to 0.38) |
| Germany | 1313 | 1.04 | 2235 | 1.07 | 1.12% |
|  | (1140 to 1536) | (0.91 to 1.21) | (1967 to 2534) | (0.95 to 1.21) | (0.44 to 1.8) |
| Ghana | 83 | 0.83 | 200 | 0.77 | -0.28% |
|  | (67 to 100) | (0.7 to 0.98) | (161 to 241) | (0.64 to 0.9) | (-0.33 to -0.23) |
| Greece | 74 | 0.53 | 155 | 0.63 | 1.22% |
|  | (63 to 88) | (0.45 to 0.62) | (135 to 180) | (0.54 to 0.74) | (0.95 to 1.48) |
| Greenland | 8 | 25.91 | 14 | 24.02 | -0.23% |
|  | (7 to 9) | (22.93 to 29.7) | (12 to 16) | (21.21 to 27.48) | (-0.3 to -0.16) |
| Grenada | 14 | 17.57 | 20 | 20.55 | 0.53% |
|  | (13 to 16) | (15.75 to 19.44) | (18 to 22) | (18.67 to 22.44) | (0.44 to 0.61) |
| Guam | 1 | 1.06 | 3 | 1.16 | 0.23% |
|  | (1 to 1) | (0.93 to 1.21) | (2 to 3) | (1.04 to 1.31) | (0.16 to 0.31) |
| Guatemala | 913 | 25.9 | 2144 | 18.75 | -1.35% |
|  | (821 to 1008) | (22.95 to 28.88) | (1915 to 2371) | (16.71 to 20.92) | (-1.49 to -1.22) |
| Guinea | 35 | 0.85 | 75 | 0.88 | 0.16% |
|  | (29 to 42) | (0.72 to 0.98) | (60 to 90) | (0.75 to 1.01) | (0.13 to 0.2) |
| Guinea-Bissau | 5 | 0.83 | 11 | 0.85 | 0.10% |
|  | (4 to 6) | (0.7 to 0.97) | (9 to 13) | (0.72 to 0.98) | (0.06 to 0.15) |
| Guyana | 39 | 9.22 | 52 | 8.87 | -0.22% |
|  | (35 to 44) | (8.28 to 10.28) | (47 to 57) | (7.95 to 9.82) | (-0.29 to -0.15) |
| Haiti | 415 | 14.44 | 874 | 13.37 | -0.23% |
|  | (373 to 457) | (12.95 to 16.03) | (793 to 955) | (12.04 to 14.85) | (-0.27 to -0.19) |
| Honduras | 504 | 22.13 | 1385 | 22.25 | 0% |
|  | (452 to 556) | (19.82 to 24.63) | (1256 to 1527) | (20.01 to 24.66) | (-0.04 to 0.04) |
| Hungary | 1497 | 11.69 | 2206 | 11.37 | -0.05% |
|  | (1308 to 1734) | (10.31 to 13.5) | (1938 to 2513) | (10.1 to 12.84) | (-0.12 to 0.01) |
| Iceland | 1 | 0.47 | 2 | 0.42 | 0.21% |
|  | (1 to 2) | (0.4 to 0.57) | (2 to 3) | (0.35 to 0.51) | (-0.24 to 0.66) |
| India | 17012 | 2.42 | 47586 | 3.33 | 1.50% |
|  | (14412 to 19585) | (2.1 to 2.76) | (40841 to 54289) | (2.89 to 3.78) | (1.21 to 1.79) |
| Indonesia | 935 | 0.81 | 1719 | 0.72 | -0.42% |
|  | (771 to 1122) | (0.7 to 0.95) | (1436 to 2035) | (0.63 to 0.84) | (-0.53 to -0.31) |
| Iran (Islamic Republic of) | 883 | 2.48 | 1992 | 2.36 | -0.08% |
|  | (745 to 1009) | (2.18 to 2.79) | (1727 to 2263) | (2.09 to 2.63) | (-0.4 to 0.25) |
| Iraq | 316 | 2.7 | 818 | 2.52 | -0.20% |
|  | (269 to 359) | (2.37 to 3.03) | (692 to 935) | (2.2 to 2.84) | (-0.27 to -0.14) |
| Ireland | 82 | 2.03 | 87 | 1.16 | -2.33% |
|  | (72 to 93) | (1.81 to 2.3) | (76 to 100) | (1.01 to 1.34) | (-2.5 to -2.15) |
| Israel | 160 | 3.4 | 568 | 4.3 | 0.69% |
|  | (142 to 181) | (3.05 to 3.82) | (509 to 626) | (3.87 to 4.74) | (0.55 to 0.83) |
| Italy | 4289 | 5.53 | 11107 | 7.36 | 1.20% |
|  | (3820 to 4825) | (4.94 to 6.17) | (9985 to 12362) | (6.65 to 8.11) | (0.63 to 1.77) |
| Jamaica | 313 | 16.38 | 575 | 17.32 | 0.11% |
|  | (284 to 346) | (14.87 to 18.13) | (526 to 623) | (15.83 to 18.82) | (0.04 to 0.18) |
| Japan | 18437 | 12.67 | 40927 | 13.03 | 0.27% |
|  | (16393 to 20647) | (11.37 to 14) | (36294 to 46350) | (11.68 to 14.33) | (0.16 to 0.38) |
| Jordan | 27 | 1.86 | 157 | 2.17 | 0.69% |
|  | (23 to 32) | (1.66 to 2.1) | (137 to 177) | (1.95 to 2.41) | (0.58 to 0.79) |
| Kazakhstan | 140 | 1.19 | 179 | 1.22 | 0.11% |
|  | (121 to 162) | (1.04 to 1.37) | (155 to 209) | (1.06 to 1.39) | (0.08 to 0.14) |
| Kenya | 80 | 0.53 | 176 | 0.47 | -1.84% |
|  | (61 to 101) | (0.44 to 0.64) | (134 to 222) | (0.39 to 0.57) | (-2.43 to -1.24) |
| Kiribati | 1 | 1.72 | 1 | 2 | 0.53% |
|  | (1 to 1) | (1.54 to 1.96) | (1 to 1) | (1.81 to 2.22) | (0.45 to 0.61) |
| Kuwait | 37 | 2.93 | 122 | 2.67 | -0.29% |
|  | (31 to 42) | (2.56 to 3.29) | (103 to 142) | (2.33 to 3.01) | (-0.38 to -0.21) |
| Kyrgyzstan | 34 | 1.17 | 51 | 1.13 | -0.08% |
|  | (29 to 40) | (1.02 to 1.35) | (44 to 60) | (0.99 to 1.32) | (-0.12 to -0.03) |
| Lao People's Democratic Republic | 25 | 1.28 | 59 | 1.38 | 0.18% |
|  | (22 to 29) | (1.14 to 1.43) | (52 to 67) | (1.23 to 1.54) | (0.15 to 0.2) |
| Latvia | 270 | 8.42 | 315 | 8.47 | 0.31% |
|  | (242 to 306) | (7.53 to 9.37) | (279 to 354) | (7.56 to 9.37) | (-0.11 to 0.73) |
| Lebanon | 65 | 2.71 | 156 | 2.55 | -0.20% |
|  | (56 to 73) | (2.37 to 3.05) | (138 to 175) | (2.25 to 2.86) | (-0.23 to -0.16) |
| Lesotho | 43 | 3.87 | 55 | 3.88 | 0.04% |
|  | (37 to 49) | (3.44 to 4.29) | (48 to 62) | (3.46 to 4.34) | (-0.04 to 0.11) |
| Liberia | 14 | 0.86 | 31 | 0.85 | 0.06% |
|  | (11 to 17) | (0.72 to 1) | (25 to 38) | (0.72 to 0.99) | (0.01 to 0.12) |
| Libya | 78 | 2.86 | 169 | 2.58 | -0.29% |
|  | (66 to 89) | (2.5 to 3.23) | (145 to 194) | (2.25 to 2.9) | (-0.34 to -0.24) |
| Lithuania | 243 | 5.75 | 289 | 5.49 | -0.16% |
|  | (216 to 278) | (5.11 to 6.51) | (255 to 331) | (4.89 to 6.23) | (-0.39 to 0.07) |
| Luxembourg | 7 | 1.41 | 12 | 1.16 | -0.64% |
|  | (7 to 8) | (1.25 to 1.61) | (11 to 14) | (1.01 to 1.32) | (-1.25 to -0.03) |
| Madagascar | 40 | 0.51 | 92 | 0.47 | -0.24% |
|  | (31 to 50) | (0.43 to 0.61) | (68 to 119) | (0.39 to 0.57) | (-0.3 to -0.19) |
| Malawi | 32 | 0.5 | 62 | 0.46 | -0.23% |
|  | (24 to 40) | (0.41 to 0.6) | (47 to 78) | (0.39 to 0.56) | (-0.29 to -0.16) |
| Malaysia | 185 | 1.98 | 867 | 3.34 | 2.27% |
|  | (165 to 206) | (1.78 to 2.21) | (783 to 946) | (3.03 to 3.66) | (2.08 to 2.47) |
| Maldives | 2 | 1.69 | 6 | 1.8 | 0.12% |
|  | (1 to 2) | (1.51 to 1.9) | (6 to 7) | (1.62 to 1.97) | (0.02 to 0.23) |
| Mali | 47 | 0.84 | 124 | 0.87 | 0.16% |
|  | (38 to 57) | (0.7 to 0.97) | (98 to 154) | (0.73 to 1.01) | (0.11 to 0.21) |
| Malta | 15 | 3.63 | 40 | 3.73 | 0.01% |
|  | (13 to 16) | (3.23 to 4.07) | (36 to 44) | (3.37 to 4.12) | (-0.26 to 0.27) |
| Marshall Islands | 0 | 1.14 | 0 | 1.22 | 0.17% |
|  | (0 to 0) | (1.01 to 1.3) | (0 to 0) | (1.1 to 1.36) | (0.13 to 0.2) |
| Mauritania | 12 | 0.85 | 26 | 0.84 | 0.01% |
|  | (10 to 14) | (0.71 to 0.99) | (21 to 31) | (0.71 to 0.97) | (-0.04 to 0.05) |
| Mauritius | 8 | 1.09 | 27 | 1.64 | 1.58% |
|  | (7 to 9) | (0.96 to 1.26) | (24 to 30) | (1.48 to 1.81) | (1.5 to 1.65) |
| Mexico | 10806 | 24.16 | 28700 | 24.09 | -0.06% |
|  | (9751 to 11920) | (21.48 to 26.91) | (25783 to 31759) | (21.62 to 26.72) | (-0.14 to 0.01) |
| Micronesia (Federated States of) | 1 | 1.13 | 1 | 1.27 | 0.38% |
|  | (0 to 1) | (0.99 to 1.28) | (1 to 1) | (1.14 to 1.43) | (0.35 to 0.41) |
| Monaco | 1 | 1.31 | 1 | 1.21 | -0.28% |
|  | (1 to 1) | (1.15 to 1.52) | (1 to 1) | (1.06 to 1.4) | (-0.34 to -0.22) |
| Mongolia | 13 | 1.19 | 23 | 1.19 | 0.02% |
|  | (11 to 15) | (1.04 to 1.39) | (20 to 27) | (1.04 to 1.37) | (0 to 0.05) |
| Montenegro | 69 | 11.72 | 93 | 10.99 | -0.23% |
|  | (60 to 79) | (10.33 to 13.65) | (81 to 107) | (9.62 to 12.52) | (-0.28 to -0.18) |
| Morocco | 489 | 2.7 | 911 | 2.57 | -0.14% |
|  | (420 to 557) | (2.37 to 3.04) | (794 to 1034) | (2.27 to 2.89) | (-0.18 to -0.09) |
| Mozambique | 44 | 0.49 | 92 | 0.47 | -0.16% |
|  | (34 to 56) | (0.41 to 0.59) | (68 to 119) | (0.38 to 0.56) | (-0.23 to -0.09) |
| Myanmar | 283 | 1.31 | 633 | 1.46 | 0.36% |
|  | (247 to 324) | (1.18 to 1.49) | (567 to 709) | (1.32 to 1.63) | (0.34 to 0.38) |
| Namibia | 40 | 4.34 | 75 | 4.15 | -0.16% |
|  | (35 to 45) | (3.9 to 4.8) | (66 to 84) | (3.72 to 4.58) | (-0.2 to -0.12) |
| Nauru | 0 | 1.13 | 0 | 1.26 | 0.37% |
|  | (0 to 0) | (1 to 1.29) | (0 to 0) | (1.13 to 1.41) | (0.35 to 0.39) |
| Nepal | 289 | 1.97 | 643 | 2.21 | 0.92% |
|  | (243 to 338) | (1.69 to 2.28) | (547 to 739) | (1.9 to 2.5) | (0.58 to 1.26) |
| Netherlands | 542 | 2.66 | 579 | 1.64 | -1.88% |
|  | (484 to 610) | (2.39 to 2.99) | (510 to 652) | (1.46 to 1.85) | (-1.98 to -1.78) |
| New Zealand | 265 | 7.14 | 580 | 7.78 | 0.11% |
|  | (237 to 296) | (6.36 to 7.99) | (521 to 655) | (7.01 to 8.75) | (-0.23 to 0.45) |
| Nicaragua | 381 | 19.16 | 990 | 19.54 | 0.04% |
|  | (340 to 425) | (17.13 to 21.31) | (895 to 1092) | (17.48 to 21.54) | (0 to 0.09) |
| Niger | 40 | 0.84 | 121 | 0.86 | 0.14% |
|  | (32 to 50) | (0.71 to 0.97) | (95 to 149) | (0.73 to 0.99) | (0.08 to 0.2) |
| Nigeria | 550 | 0.87 | 1270 | 0.87 | 0.14% |
|  | (447 to 659) | (0.73 to 1.01) | (1022 to 1535) | (0.74 to 1) | (0.06 to 0.23) |
| Niue | 0 | 1.18 | 0 | 1.17 | -0.01% |
|  | (0 to 0) | (1.04 to 1.33) | (0 to 0) | (1.05 to 1.32) | (-0.03 to 0.01) |
| North Macedonia | 196 | 11.53 | 290 | 10.96 | -0.15% |
|  | (173 to 223) | (10.11 to 13.4) | (252 to 340) | (9.68 to 12.58) | (-0.2 to -0.09) |
| Northern Mariana Islands | 0 | 1.47 | 1 | 1.34 | -0.42% |
|  | (0 to 0) | (1.3 to 1.66) | (0 to 1) | (1.2 to 1.5) | (-0.52 to -0.31) |
| Norway | 482 | 8.51 | 646 | 8.2 | 1.13% |
|  | (434 to 538) | (7.65 to 9.43) | (579 to 721) | (7.3 to 9.14) | (0.23 to 2.05) |
| Oman | 39 | 3.01 | 117 | 3.09 | 0.15% |
|  | (33 to 45) | (2.64 to 3.38) | (99 to 137) | (2.76 to 3.45) | (0.11 to 0.2) |
| Pakistan | 1739 | 2.08 | 3935 | 1.92 | -0.27% |
|  | (1447 to 2004) | (1.77 to 2.39) | (3290 to 4560) | (1.64 to 2.21) | (-0.34 to -0.19) |
| Palau | 0 | 0.88 | 0 | 0.78 | -0.41% |
|  | (0 to 0) | (0.76 to 1.03) | (0 to 0) | (0.67 to 0.92) | (-0.5 to -0.31) |
| Palestine | 32 | 2.55 | 93 | 2.46 | -0.12% |
|  | (27 to 37) | (2.22 to 2.87) | (79 to 107) | (2.16 to 2.76) | (-0.18 to -0.06) |
| Panama | 377 | 23.44 | 1129 | 24.7 | 0.23% |
|  | (341 to 413) | (21.1 to 25.99) | (1020 to 1240) | (22.18 to 27.15) | (0.21 to 0.26) |
| Papua New Guinea | 18 | 0.98 | 52 | 1.01 | 0.10% |
|  | (15 to 22) | (0.86 to 1.12) | (44 to 61) | (0.9 to 1.14) | (0.07 to 0.14) |
| Paraguay | 278 | 10.56 | 696 | 11.4 | 0.33% |
|  | (247 to 311) | (9.41 to 11.86) | (626 to 776) | (10.24 to 12.69) | (0.29 to 0.37) |
| Peru | 1099 | 8.55 | 2211 | 6.24 | -1.38% |
|  | (989 to 1212) | (7.68 to 9.53) | (1968 to 2469) | (5.55 to 6.98) | (-1.49 to -1.27) |
| Philippines | 729 | 2.51 | 2138 | 2.8 | 0.51% |
|  | (642 to 818) | (2.26 to 2.79) | (1927 to 2344) | (2.55 to 3.07) | (0.34 to 0.68) |
| Poland | 5351 | 13.61 | 12117 | 16.89 | 0.86% |
|  | (4747 to 6122) | (12.05 to 15.5) | (11067 to 13420) | (15.53 to 18.55) | (0.79 to 0.93) |
| Portugal | 103 | 0.8 | 131 | 0.52 | -2.43% |
|  | (89 to 121) | (0.7 to 0.93) | (113 to 154) | (0.44 to 0.6) | (-2.96 to -1.9) |
| Puerto Rico | 459 | 13.17 | 723 | 9.63 | -1.22% |
|  | (410 to 513) | (11.81 to 14.6) | (649 to 801) | (8.7 to 10.62) | (-1.28 to -1.16) |
| Qatar | 30 | 8.03 | 235 | 7.74 | -0.44% |
|  | (25 to 34) | (7.13 to 9.04) | (199 to 272) | (6.83 to 8.7) | (-0.74 to -0.14) |
| Republic of Korea | 4448 | 14.67 | 10651 | 13.77 | -0.30% |
|  | (3971 to 4940) | (13.26 to 16.12) | (9463 to 11896) | (12.38 to 15.28) | (-0.4 to -0.2) |
| Republic of Moldova | 279 | 6.99 | 360 | 6.74 | -0.10% |
|  | (247 to 319) | (6.24 to 7.95) | (318 to 411) | (5.96 to 7.6) | (-0.14 to -0.06) |
| Romania | 2705 | 11.79 | 4011 | 10.96 | -0.11% |
|  | (2393 to 3140) | (10.4 to 13.62) | (3482 to 4602) | (9.78 to 12.4) | (-0.27 to 0.04) |
| Russian Federation | 10778 | 6.84 | 12498 | 6.02 | -0.42% |
|  | (9514 to 12220) | (6.1 to 7.67) | (11046 to 14165) | (5.37 to 6.73) | (-0.5 to -0.34) |
| Rwanda | 23 | 0.51 | 46 | 0.47 | -0.21% |
|  | (18 to 30) | (0.42 to 0.61) | (35 to 58) | (0.39 to 0.57) | (-0.26 to -0.17) |
| Saint Kitts and Nevis | 7 | 19.26 | 11 | 19.88 | 0.02% |
|  | (6 to 8) | (17.19 to 21.24) | (10 to 12) | (17.88 to 21.76) | (-0.06 to 0.11) |
| Saint Lucia | 14 | 16.87 | 38 | 16.75 | -0.20% |
|  | (13 to 16) | (15.08 to 18.64) | (34 to 41) | (15.22 to 18.29) | (-0.29 to -0.11) |
| Saint Vincent and the Grenadines | 10 | 13.73 | 24 | 18.12 | 0.74% |
|  | (9 to 11) | (12.25 to 15.43) | (21 to 26) | (16.36 to 19.85) | (0.65 to 0.82) |
| Samoa | 1 | 1.19 | 2 | 1.22 | 0.16% |
|  | (1 to 1) | (1.06 to 1.35) | (1 to 2) | (1.09 to 1.37) | (0.14 to 0.19) |
| San Marino | 0 | 1.28 | 1 | 1.17 | -0.25% |
|  | (0 to 1) | (1.11 to 1.48) | (1 to 1) | (1.01 to 1.36) | (-0.34 to -0.15) |
| Sao Tome and Principe | 1 | 0.83 | 1 | 0.88 | 0.26% |
|  | (1 to 1) | (0.7 to 0.97) | (1 to 2) | (0.75 to 1.02) | (0.22 to 0.3) |
| Saudi Arabia | 425 | 5.99 | 1340 | 6.8 | 0.38% |
|  | (374 to 471) | (5.38 to 6.62) | (1176 to 1484) | (6.2 to 7.49) | (0.26 to 0.51) |
| Senegal | 42 | 0.87 | 98 | 0.91 | 0.21% |
|  | (34 to 51) | (0.73 to 1.01) | (80 to 118) | (0.78 to 1.05) | (0.17 to 0.25) |
| Serbia | 686 | 7.81 | 1392 | 8.71 | 0.39% |
|  | (606 to 782) | (6.88 to 8.9) | (1223 to 1570) | (7.67 to 9.78) | (0.36 to 0.43) |
| Seychelles | 1 | 1.17 | 1 | 1.4 | 0.72% |
|  | (1 to 1) | (1.04 to 1.32) | (1 to 2) | (1.26 to 1.57) | (0.65 to 0.79) |
| Sierra Leone | 24 | 0.84 | 51 | 0.85 | 0.09% |
|  | (20 to 29) | (0.71 to 0.97) | (41 to 61) | (0.72 to 0.99) | (0.04 to 0.14) |
| Singapore | 379 | 16.64 | 1008 | 13.43 | -0.46% |
|  | (340 to 416) | (15.05 to 18.22) | (903 to 1118) | (12.07 to 14.82) | (-0.6 to -0.31) |
| Slovakia | 726 | 13.1 | 1226 | 14.12 | 0.16% |
|  | (635 to 836) | (11.52 to 15.09) | (1075 to 1388) | (12.47 to 15.88) | (-0.02 to 0.34) |
| Slovenia | 270 | 11.76 | 577 | 12.04 | 0.11% |
|  | (236 to 312) | (10.33 to 13.5) | (497 to 659) | (10.63 to 13.72) | (-0.13 to 0.35) |
| Solomon Islands | 1 | 0.98 | 4 | 1.08 | 0.32% |
|  | (1 to 2) | (0.86 to 1.12) | (3 to 4) | (0.97 to 1.22) | (0.31 to 0.33) |
| Somalia | 24 | 0.49 | 60 | 0.46 | -0.25% |
|  | (18 to 31) | (0.41 to 0.59) | (44 to 78) | (0.38 to 0.55) | (-0.31 to -0.19) |
| South Africa | 1211 | 4.43 | 1968 | 3.96 | -0.49% |
|  | (1060 to 1355) | (3.98 to 4.87) | (1748 to 2190) | (3.54 to 4.36) | (-0.63 to -0.35) |
| South Sudan | 20 | 0.52 | 30 | 0.47 | -0.31% |
|  | (16 to 26) | (0.44 to 0.63) | (23 to 39) | (0.39 to 0.57) | (-0.37 to -0.25) |
| Spain | 614 | 1.16 | 1517 | 1.43 | 0.86% |
|  | (545 to 702) | (1.03 to 1.32) | (1352 to 1681) | (1.29 to 1.59) | (0.66 to 1.06) |
| Sri Lanka | 97 | 0.83 | 198 | 0.8 | -0.10% |
|  | (80 to 115) | (0.71 to 0.98) | (171 to 232) | (0.7 to 0.93) | (-0.18 to -0.01) |
| Sudan | 335 | 2.67 | 755 | 2.51 | -0.20% |
|  | (283 to 382) | (2.32 to 3.01) | (638 to 868) | (2.19 to 2.83) | (-0.27 to -0.14) |
| Suriname | 46 | 18.1 | 119 | 20.19 | 0.32% |
|  | (41 to 50) | (16.35 to 19.97) | (108 to 129) | (18.39 to 22.04) | (0.29 to 0.35) |
| Sweden | 393 | 2.86 | 501 | 2.81 | -0.06% |
|  | (349 to 446) | (2.54 to 3.23) | (443 to 570) | (2.49 to 3.2) | (-0.66 to 0.54) |
| Switzerland | 238 | 2.37 | 360 | 2.14 | -0.43% |
|  | (210 to 274) | (2.1 to 2.72) | (324 to 404) | (1.9 to 2.41) | (-1.23 to 0.37) |
| Syrian Arab Republic | 209 | 2.71 | 335 | 2.48 | -0.25% |
|  | (177 to 239) | (2.38 to 3.06) | (287 to 384) | (2.17 to 2.79) | (-0.35 to -0.15) |
| Taiwan (Province of China) | 427 | 3.39 | 1281 | 3.05 | -0.78% |
|  | (383 to 475) | (3.05 to 3.77) | (1156 to 1423) | (2.75 to 3.39) | (-1.08 to -0.49) |
| Tajikistan | 34 | 1.17 | 62 | 1.11 | -0.15% |
|  | (29 to 39) | (1.01 to 1.35) | (53 to 73) | (0.97 to 1.28) | (-0.19 to -0.11) |
| Thailand | 452 | 1.3 | 1942 | 1.88 | 1.45% |
|  | (396 to 520) | (1.15 to 1.48) | (1756 to 2158) | (1.71 to 2.07) | (1.31 to 1.59) |
| Timor-Leste | 4 | 1.14 | 10 | 1.27 | 0.41% |
|  | (3 to 4) | (1.01 to 1.3) | (9 to 12) | (1.15 to 1.44) | (0.37 to 0.44) |
| Togo | 19 | 0.84 | 48 | 0.84 | 0.04% |
|  | (15 to 23) | (0.71 to 0.97) | (39 to 58) | (0.71 to 0.97) | (-0.01 to 0.09) |
| Tokelau | 0 | 1.09 | 0 | 1.14 | 0.17% |
|  | (0 to 0) | (0.96 to 1.24) | (0 to 0) | (1.02 to 1.28) | (0.16 to 0.18) |
| Tonga | 1 | 1.06 | 1 | 1.16 | 0.31% |
|  | (0 to 1) | (0.93 to 1.22) | (1 to 1) | (1.04 to 1.3) | (0.27 to 0.34) |
| Trinidad and Tobago | 95 | 11.75 | 177 | 10.13 | -0.56% |
|  | (86 to 106) | (10.53 to 13.12) | (159 to 196) | (9.12 to 11.24) | (-0.6 to -0.52) |
| Tunisia | 165 | 2.7 | 323 | 2.54 | -0.19% |
|  | (140 to 187) | (2.37 to 3.05) | (282 to 365) | (2.22 to 2.86) | (-0.24 to -0.14) |
| Turkmenistan | 22 | 1.17 | 43 | 1.14 | -0.03% |
|  | (19 to 26) | (1.02 to 1.35) | (37 to 50) | (1 to 1.32) | (-0.06 to 0) |
| Tuvalu | 0 | 1.13 | 0 | 1.17 | 0.06% |
|  | (0 to 0) | (1 to 1.29) | (0 to 0) | (1.05 to 1.31) | (0.04 to 0.07) |
| Uganda | 54 | 0.51 | 131 | 0.48 | -0.18% |
|  | (41 to 69) | (0.42 to 0.61) | (97 to 168) | (0.4 to 0.57) | (-0.24 to -0.13) |
| Ukraine | 4149 | 6.7 | 3949 | 5.95 | -0.37% |
|  | (3631 to 4756) | (5.92 to 7.62) | (3484 to 4510) | (5.3 to 6.68) | (-0.47 to -0.28) |
| United Arab Emirates | 43 | 3.17 | 311 | 3.01 | -0.15% |
|  | (36 to 51) | (2.8 to 3.56) | (251 to 373) | (2.63 to 3.39) | (-0.23 to -0.07) |
| United Kingdom | 4549 | 5.53 | 9244 | 7.6 | 0.10% |
|  | (4076 to 5093) | (4.95 to 6.12) | (8230 to 10438) | (6.78 to 8.53) | (-0.2 to 0.4) |
| United Republic of Tanzania | 85 | 0.51 | 195 | 0.48 | -0.19% |
|  | (65 to 108) | (0.43 to 0.61) | (147 to 247) | (0.4 to 0.58) | (-0.24 to -0.14) |
| United States of America | 102430 | 31.44 | 188420 | 31.83 | -0.03% |
|  | (90224 to 115132) | (27.84 to 35.19) | (168469 to 207804) | (28.67 to 35.04) | (-0.14 to 0.09) |
| United States Virgin Islands | 11 | 14.63 | 30 | 17.25 | 0.53% |
|  | (10 to 12) | (13.19 to 16.17) | (27 to 33) | (15.69 to 18.88) | (0.46 to 0.6) |
| Uruguay | 196 | 5.15 | 333 | 5.38 | 0.08% |
|  | (175 to 220) | (4.6 to 5.73) | (297 to 367) | (4.81 to 5.93) | (-0.03 to 0.2) |
| Uzbekistan | 143 | 1.16 | 268 | 1.15 | 0.01% |
|  | (123 to 167) | (1.02 to 1.34) | (229 to 314) | (1 to 1.31) | (-0.04 to 0.05) |
| Vanuatu | 1 | 0.96 | 2 | 1.07 | 0.40% |
|  | (1 to 1) | (0.84 to 1.11) | (2 to 2) | (0.96 to 1.22) | (0.38 to 0.43) |
| Venezuela (Bolivarian Republic of) | 2233 | 19.13 | 5209 | 18.1 | -0.08% |
|  | (1992 to 2480) | (17.12 to 21.48) | (4600 to 5866) | (16.09 to 20.44) | (-0.17 to 0.01) |
| Viet Nam | 403 | 0.94 | 813 | 0.87 | -0.26% |
|  | (341 to 476) | (0.81 to 1.1) | (699 to 966) | (0.76 to 1.02) | (-0.3 to -0.21) |
| Yemen | 198 | 2.67 | 553 | 2.45 | -0.28% |
|  | (167 to 230) | (2.33 to 3.02) | (466 to 638) | (2.15 to 2.73) | (-0.35 to -0.21) |
| Zambia | 25 | 0.51 | 61 | 0.48 | -0.19% |
|  | (19 to 32) | (0.42 to 0.61) | (46 to 78) | (0.4 to 0.57) | (-0.24 to -0.14) |
| Zimbabwe | 271 | 4.03 | 408 | 3.76 | -0.23% |
|  | (232 to 308) | (3.6 to 4.48) | (352 to 464) | (3.35 to 4.18) | (-0.29 to -0.17) |

| Table S3: Death of decubitus ulcers in 1990 and 2021 and their temporal trends from 1990 to 2021 at the national and territorial levels. | | | | | |
| --- | --- | --- | --- | --- | --- |
| Location | Counts 1990  (95% UI) | Age-standardized death rate (95% UI) | Counts 2021 (95% UI) | Age-standardized death rate (95% UI) | EAPC (95% CI) |
| Afghanistan | 58 | 1.08 | 102 | 1.44 | 0.82% |
|  | (35 to 94) | (0.63 to 1.87) | (62 to 145) | (0.89 to 2.13) | (0.69 to 0.95) |
| Albania | 0 | 0.03 | 2 | 0.04 | 0.15% |
|  | (0 to 1) | (0.01 to 0.05) | (1 to 3) | (0.02 to 0.08) | (-0.48 to 0.77) |
| Algeria | 46 | 0.62 | 226 | 1 | 1.36% |
|  | (31 to 68) | (0.45 to 0.88) | (164 to 300) | (0.73 to 1.32) | (1.04 to 1.68) |
| American Samoa | 1 | 3.55 | 2 | 4.37 | 0.76% |
|  | (0 to 1) | (1.76 to 5.36) | (1 to 3) | (2.47 to 7.17) | (0.42 to 1.11) |
| Andorra | 0 | 0.1 | 0 | 0.07 | -1.12% |
|  | (0 to 0) | (0.07 to 0.14) | (0 to 0) | (0.05 to 0.09) | (-1.23 to -1) |
| Angola | 36 | 1.2 | 93 | 1.11 | -1.54% |
|  | (14 to 57) | (0.47 to 1.94) | (35 to 167) | (0.49 to 1.96) | (-2.13 to -0.94) |
| Antigua and Barbuda | 2 | 3.82 | 6 | 6.67 | 2.62% |
|  | (2 to 3) | (3.34 to 4.35) | (5 to 6) | (6 to 7.25) | (2.3 to 2.95) |
| Argentina | 243 | 0.85 | 1235 | 2.09 | 3.53% |
|  | (216 to 269) | (0.75 to 0.94) | (1075 to 1356) | (1.82 to 2.3) | (2.99 to 4.08) |
| Armenia | 1 | 0.02 | 4 | 0.08 | 4.15% |
|  | (0 to 1) | (0.01 to 0.03) | (3 to 5) | (0.06 to 0.1) | (3.26 to 5.05) |
| Australia | 43 | 0.25 | 57 | 0.1 | -2.33% |
|  | (37 to 47) | (0.21 to 0.27) | (46 to 66) | (0.08 to 0.12) | (-3.1 to -1.54) |
| Austria | 19 | 0.15 | 20 | 0.08 | -2.36% |
|  | (17 to 20) | (0.14 to 0.17) | (16 to 24) | (0.06 to 0.1) | (-2.69 to -2.03) |
| Azerbaijan | 1 | 0.01 | 1 | 0.01 | 0.37% |
|  | (0 to 2) | (0 to 0.03) | (0 to 3) | (0 to 0.03) | (-0.3 to 1.05) |
| Bahamas | 9 | 6.34 | 24 | 7.09 | 0.65% |
|  | (8 to 9) | (5.77 to 6.93) | (20 to 28) | (5.9 to 8.49) | (0.45 to 0.86) |
| Bahrain | 4 | 5.5 | 15 | 5.11 | -1.52% |
|  | (2 to 7) | (2.59 to 9.64) | (7 to 25) | (2.29 to 8.3) | (-1.91 to -1.13) |
| Bangladesh | 150 | 0.58 | 591 | 0.82 | 1.14% |
|  | (69 to 278) | (0.24 to 1.13) | (271 to 1008) | (0.35 to 1.46) | (0.88 to 1.4) |
| Barbados | 52 | 17.13 | 63 | 12.41 | -0.80% |
|  | (46 to 57) | (15.25 to 18.93) | (51 to 74) | (10.11 to 14.59) | (-0.94 to -0.65) |
| Belarus | 8 | 0.06 | 17 | 0.11 | 2.02% |
|  | (6 to 10) | (0.05 to 0.08) | (14 to 20) | (0.09 to 0.13) | (1.68 to 2.36) |
| Belgium | 359 | 2.3 | 242 | 0.75 | -2.43% |
|  | (309 to 393) | (1.99 to 2.53) | (191 to 281) | (0.6 to 0.87) | (-2.98 to -1.88) |
| Belize | 1 | 1.08 | 3 | 1.06 | 0.37% |
|  | (1 to 1) | (0.82 to 1.3) | (2 to 3) | (0.92 to 1.21) | (-0.02 to 0.76) |
| Benin | 0 | 0.01 | 1 | 0.01 | 0.09% |
|  | (0 to 0) | (0 to 0.02) | (0 to 2) | (0 to 0.02) | (-0.37 to 0.55) |
| Bermuda | 1 | 1.4 | 2 | 1.43 | 0.37% |
|  | (1 to 1) | (1.2 to 1.6) | (2 to 3) | (1.19 to 1.73) | (0.04 to 0.69) |
| Bhutan | 1 | 0.37 | 2 | 0.37 | 0.02% |
|  | (0 to 1) | (0.18 to 0.63) | (1 to 4) | (0.17 to 0.63) | (-0.02 to 0.06) |
| Bolivia (Plurinational State of) | 14 | 0.56 | 47 | 0.67 | 0.52% |
|  | (9 to 20) | (0.4 to 0.83) | (29 to 68) | (0.42 to 0.96) | (0.46 to 0.57) |
| Bosnia and Herzegovina | 5 | 0.13 | 8 | 0.13 | -0.70% |
|  | (3 to 7) | (0.08 to 0.19) | (4 to 12) | (0.07 to 0.18) | (-0.85 to -0.55) |
| Botswana | 5 | 1.44 | 17 | 1.71 | 1.21% |
|  | (3 to 8) | (0.9 to 2.36) | (11 to 24) | (1.17 to 2.55) | (0.93 to 1.49) |
| Brazil | 498 | 0.72 | 1936 | 0.82 | 0.92% |
|  | (461 to 525) | (0.65 to 0.76) | (1658 to 2179) | (0.7 to 0.92) | (0.79 to 1.06) |
| Brunei Darussalam | 1 | 1.47 | 2 | 1.43 | 0.16% |
|  | (1 to 1) | (1.09 to 1.87) | (2 to 3) | (1.01 to 1.93) | (-0.1 to 0.43) |
| Bulgaria | 1 | 0.01 | 4 | 0.03 | 5.16% |
|  | (0 to 1) | (0.01 to 0.01) | (3 to 5) | (0.02 to 0.03) | (4.05 to 6.29) |
| Burkina Faso | 6 | 0.21 | 16 | 0.22 | -0.02% |
|  | (1 to 16) | (0.02 to 0.51) | (1 to 39) | (0.02 to 0.55) | (-0.93 to 0.89) |
| Burundi | 46 | 2.17 | 71 | 1.75 | -0.65% |
|  | (24 to 88) | (1.12 to 4.17) | (35 to 130) | (0.87 to 3.23) | (-0.81 to -0.5) |
| Cabo Verde | 0 | 0.02 | 0 | 0.1 | 5.30% |
|  | (0 to 0) | (0.01 to 0.03) | (0 to 1) | (0.04 to 0.18) | (4.96 to 5.65) |
| Cambodia | 62 | 1.87 | 164 | 1.96 | 0.06% |
|  | (33 to 101) | (0.99 to 3.2) | (101 to 228) | (1.26 to 2.67) | (0.02 to 0.11) |
| Cameroon | 0 | 0.01 | 2 | 0.01 | 0.44% |
|  | (0 to 1) | (0 to 0.03) | (0 to 5) | (0 to 0.03) | (-0.02 to 0.9) |
| Canada | 78 | 0.25 | 73 | 0.09 | -3.50% |
|  | (68 to 85) | (0.21 to 0.27) | (60 to 83) | (0.07 to 0.1) | (-3.78 to -3.21) |
| Central African Republic | 12 | 1.46 | 21 | 1.36 | -0.04% |
|  | (5 to 21) | (0.62 to 2.41) | (8 to 37) | (0.53 to 2.3) | (-0.13 to 0.04) |
| Chad | 0 | 0.01 | 1 | 0.01 | 0.78% |
|  | (0 to 0) | (0 to 0.02) | (0 to 2) | (0 to 0.02) | (0.38 to 1.18) |
| Chile | 36 | 0.44 | 338 | 1.27 | 3.79% |
|  | (32 to 39) | (0.38 to 0.48) | (284 to 383) | (1.07 to 1.44) | (3.12 to 4.47) |
| China | 243 | 0.08 | 3132 | 0.21 | 2.26% |
|  | (125 to 703) | (0.04 to 0.21) | (1563 to 4127) | (0.11 to 0.27) | (1.17 to 3.35) |
| Colombia | 277 | 1.75 | 257 | 0.44 | -3.12% |
|  | (256 to 296) | (1.61 to 1.87) | (199 to 322) | (0.35 to 0.56) | (-3.74 to -2.49) |
| Comoros | 3 | 1.93 | 8 | 1.86 | -0.17% |
|  | (2 to 6) | (0.96 to 3.49) | (4 to 14) | (0.96 to 3.2) | (-0.27 to -0.06) |
| Congo | 12 | 1.56 | 26 | 1.38 | -0.18% |
|  | (4 to 21) | (0.61 to 2.61) | (9 to 47) | (0.52 to 2.38) | (-0.34 to -0.02) |
| Cook Islands | 0 | 0.43 | 0 | 0.48 | 0.27% |
|  | (0 to 0) | (0.27 to 0.64) | (0 to 0) | (0.24 to 0.73) | (0.05 to 0.5) |
| Costa Rica | 16 | 0.97 | 42 | 0.74 | -0.66% |
|  | (14 to 17) | (0.84 to 1.08) | (35 to 48) | (0.61 to 0.84) | (-0.86 to -0.45) |
| Croatia | 1 | 0.03 | 7 | 0.08 | 3.98% |
|  | (1 to 2) | (0.02 to 0.03) | (6 to 9) | (0.06 to 0.09) | (3.07 to 4.9) |
| Cuba | 70 | 0.77 | 82 | 0.38 | -2.44% |
|  | (62 to 77) | (0.68 to 0.85) | (68 to 95) | (0.31 to 0.43) | (-2.67 to -2.2) |
| Cyprus | 19 | 4.3 | 37 | 2.53 | -1.65% |
|  | (12 to 26) | (2.73 to 5.74) | (29 to 48) | (1.97 to 3.22) | (-1.87 to -1.44) |
| Czechia | 16 | 0.12 | 86 | 0.37 | 3.70% |
|  | (14 to 18) | (0.11 to 0.14) | (71 to 101) | (0.31 to 0.44) | (2.44 to 4.96) |
| Democratic People's Republic of Korea | 41 | 0.42 | 90 | 0.37 | -0.07% |
|  | (27 to 59) | (0.28 to 0.61) | (64 to 123) | (0.26 to 0.5) | (-0.22 to 0.07) |
| Democratic Republic of the Congo | 141 | 1.26 | 350 | 1.31 | -0.01% |
|  | (36 to 238) | (0.35 to 2.05) | (127 to 594) | (0.48 to 2.23) | (-0.06 to 0.04) |
| Denmark | 15 | 0.17 | 11 | 0.08 | -1.98% |
|  | (13 to 16) | (0.15 to 0.18) | (9 to 13) | (0.06 to 0.09) | (-2.63 to -1.32) |
| Djibouti | 2 | 1.69 | 9 | 1.91 | 0.56% |
|  | (1 to 4) | (0.81 to 3.46) | (5 to 14) | (1.12 to 2.92) | (0.46 to 0.67) |
| Dominica | 4 | 6.87 | 5 | 7.39 | 0.12% |
|  | (3 to 5) | (5.19 to 9.42) | (4 to 7) | (5.63 to 9.41) | (0 to 0.25) |
| Dominican Republic | 4 | 0.13 | 9 | 0.09 | -1.25% |
|  | (1 to 7) | (0.05 to 0.25) | (4 to 18) | (0.04 to 0.19) | (-1.51 to -0.99) |
| Ecuador | 5 | 0.1 | 105 | 0.75 | 3.77% |
|  | (4 to 5) | (0.09 to 0.11) | (83 to 132) | (0.6 to 0.93) | (1.99 to 5.57) |
| Egypt | 189 | 1.19 | 390 | 1.03 | 0.25% |
|  | (120 to 340) | (0.74 to 2.32) | (306 to 476) | (0.81 to 1.23) | (0.07 to 0.44) |
| El Salvador | 19 | 0.66 | 43 | 0.61 | -1.40% |
|  | (12 to 26) | (0.41 to 0.9) | (27 to 62) | (0.38 to 0.89) | (-1.69 to -1.11) |
| Equatorial Guinea | 2 | 1.3 | 5 | 1.25 | 0.01% |
|  | (1 to 3) | (0.54 to 2.04) | (1 to 9) | (0.44 to 2.48) | (-0.08 to 0.1) |
| Eritrea | 21 | 2.3 | 53 | 2.56 | 0.44% |
|  | (12 to 37) | (1.24 to 4.48) | (32 to 87) | (1.54 to 4.17) | (0.38 to 0.5) |
| Estonia | 1 | 0.04 | 4 | 0.15 | 2.96% |
|  | (1 to 1) | (0.03 to 0.05) | (4 to 5) | (0.12 to 0.17) | (2.14 to 3.77) |
| Eswatini | 4 | 2.11 | 11 | 2.87 | 2.13% |
|  | (2 to 7) | (1.17 to 3.65) | (7 to 17) | (1.72 to 4.39) | (1.66 to 2.6) |
| Ethiopia | 357 | 2.07 | 528 | 1.34 | -1.56% |
|  | (204 to 601) | (1.19 to 3.48) | (317 to 776) | (0.84 to 1.98) | (-1.7 to -1.43) |
| Fiji | 2 | 0.77 | 4 | 1.04 | 0.80% |
|  | (1 to 3) | (0.45 to 1.26) | (2 to 7) | (0.62 to 1.45) | (0.43 to 1.17) |
| Finland | 4 | 0.06 | 3 | 0.02 | -3.52% |
|  | (3 to 4) | (0.05 to 0.06) | (2 to 3) | (0.01 to 0.02) | (-4.09 to -2.94) |
| France | 1771 | 1.99 | 943 | 0.43 | -4.23% |
|  | (1543 to 1924) | (1.74 to 2.14) | (758 to 1071) | (0.35 to 0.48) | (-4.84 to -3.62) |
| Gabon | 7 | 1.44 | 11 | 1.42 | 0.33% |
|  | (3 to 11) | (0.55 to 2.3) | (3 to 20) | (0.47 to 2.6) | (0.13 to 0.54) |
| Gambia | 0 | 0.01 | 0 | 0.01 | 0.70% |
|  | (0 to 0) | (0 to 0.02) | (0 to 0) | (0 to 0.02) | (0.16 to 1.23) |
| Georgia | 0 | 0 | 6 | 0.1 | 14.32% |
|  | (0 to 0) | (0 to 0) | (5 to 8) | (0.08 to 0.12) | (11.65 to 17.06) |
| Germany | 283 | 0.21 | 636 | 0.25 | 0.40% |
|  | (249 to 316) | (0.19 to 0.24) | (514 to 724) | (0.21 to 0.29) | (-0.14 to 0.94) |
| Ghana | 7 | 0.17 | 17 | 0.09 | -2.69% |
|  | (1 to 15) | (0.02 to 0.33) | (1 to 69) | (0.01 to 0.47) | (-3.21 to -2.17) |
| Greece | 10 | 0.08 | 29 | 0.08 | -1.65% |
|  | (9 to 11) | (0.07 to 0.08) | (24 to 33) | (0.07 to 0.09) | (-2.01 to -1.29) |
| Greenland | 0 | 0.14 | 0 | 0.07 | -4.10% |
|  | (0 to 0) | (0.03 to 0.24) | (0 to 0) | (0.01 to 0.13) | (-4.91 to -3.29) |
| Grenada | 6 | 6.65 | 7 | 8.14 | 0.90% |
|  | (5 to 6) | (5.75 to 7.42) | (6 to 7) | (7.05 to 8.96) | (0.74 to 1.05) |
| Guam | 0 | 0.42 | 1 | 0.23 | -2.14% |
|  | (0 to 0) | (0.23 to 0.76) | (0 to 1) | (0.08 to 0.35) | (-2.82 to -1.46) |
| Guatemala | 38 | 1.57 | 48 | 0.55 | -2.59% |
|  | (33 to 42) | (1.36 to 1.72) | (40 to 56) | (0.47 to 0.64) | (-3.13 to -2.06) |
| Guinea | 0 | 0.01 | 1 | 0.01 | -0.14% |
|  | (0 to 1) | (0 to 0.02) | (0 to 1) | (0 to 0.02) | (-0.6 to 0.31) |
| Guinea-Bissau | 0 | 0.01 | 0 | 0.01 | -0.20% |
|  | (0 to 0) | (0 to 0.03) | (0 to 0) | (0 to 0.02) | (-0.72 to 0.33) |
| Guyana | 1 | 0.37 | 4 | 0.9 | 3.03% |
|  | (1 to 1) | (0.33 to 0.4) | (3 to 5) | (0.71 to 1.11) | (2.12 to 3.95) |
| Haiti | 105 | 4.48 | 230 | 4.35 | -0.07% |
|  | (67 to 164) | (2.78 to 7.48) | (139 to 368) | (2.63 to 7.06) | (-0.14 to -0.01) |
| Honduras | 11 | 0.65 | 43 | 0.86 | 0.92% |
|  | (7 to 18) | (0.39 to 1.08) | (30 to 57) | (0.62 to 1.14) | (0.76 to 1.09) |
| Hungary | 5 | 0.04 | 32 | 0.15 | 4% |
|  | (5 to 6) | (0.04 to 0.04) | (27 to 37) | (0.13 to 0.17) | (2.6 to 5.42) |
| Iceland | 0 | 0.05 | 0 | 0.02 | -2.77% |
|  | (0 to 0) | (0.04 to 0.05) | (0 to 0) | (0.01 to 0.02) | (-3.36 to -2.17) |
| India | 2263 | 0.53 | 5441 | 0.52 | -1.51% |
|  | (1266 to 3252) | (0.28 to 0.76) | (2999 to 7232) | (0.28 to 0.69) | (-2.33 to -0.68) |
| Indonesia | 253 | 0.32 | 1083 | 0.7 | 1.76% |
|  | (140 to 470) | (0.18 to 0.61) | (534 to 1468) | (0.37 to 0.97) | (1.57 to 1.95) |
| Iran (Islamic Republic of) | 118 | 0.74 | 441 | 0.7 | -0.30% |
|  | (80 to 162) | (0.51 to 1.06) | (335 to 508) | (0.54 to 0.8) | (-0.43 to -0.17) |
| Iraq | 13 | 0.18 | 29 | 0.2 | -0.71% |
|  | (7 to 19) | (0.1 to 0.27) | (18 to 42) | (0.13 to 0.28) | (-1.03 to -0.39) |
| Ireland | 30 | 0.8 | 19 | 0.22 | -3.07% |
|  | (28 to 32) | (0.73 to 0.86) | (15 to 22) | (0.17 to 0.25) | (-3.73 to -2.4) |
| Israel | 122 | 2.92 | 470 | 3.23 | 1% |
|  | (108 to 134) | (2.56 to 3.21) | (377 to 530) | (2.62 to 3.62) | (0.56 to 1.44) |
| Italy | 196 | 0.24 | 609 | 0.28 | 1.09% |
|  | (170 to 210) | (0.2 to 0.26) | (480 to 680) | (0.23 to 0.31) | (0.65 to 1.52) |
| Jamaica | 78 | 4.18 | 161 | 4.57 | 0.09% |
|  | (67 to 86) | (3.6 to 4.64) | (126 to 195) | (3.62 to 5.57) | (-0.11 to 0.29) |
| Japan | 228 | 0.17 | 663 | 0.11 | -4.12% |
|  | (194 to 245) | (0.14 to 0.19) | (517 to 751) | (0.09 to 0.12) | (-5.05 to -3.18) |
| Jordan | 9 | 1 | 45 | 1.01 | 0.20% |
|  | (7 to 12) | (0.79 to 1.32) | (36 to 57) | (0.79 to 1.26) | (0.05 to 0.35) |
| Kazakhstan | 2 | 0.02 | 4 | 0.03 | 1.62% |
|  | (2 to 3) | (0.01 to 0.03) | (3 to 6) | (0.03 to 0.04) | (1.26 to 1.99) |
| Kenya | 118 | 1.64 | 369 | 2.06 | 0.74% |
|  | (53 to 305) | (0.73 to 4.23) | (223 to 631) | (1.29 to 3.57) | (0.65 to 0.83) |
| Kiribati | 1 | 4.66 | 3 | 5.26 | 0.52% |
|  | (1 to 3) | (1.9 to 10.14) | (1 to 5) | (2.65 to 9.36) | (0.45 to 0.59) |
| Kuwait | 0 | 0.13 | 6 | 0.27 | 1.32% |
|  | (0 to 1) | (0.1 to 0.15) | (4 to 7) | (0.2 to 0.34) | (0.01 to 2.66) |
| Kyrgyzstan | 0 | 0 | 0 | 0.01 | 8.47% |
|  | (0 to 0) | (0 to 0) | (0 to 0) | (0 to 0.01) | (6.39 to 10.59) |
| Lao People's Democratic Republic | 29 | 2.02 | 63 | 1.86 | -0.25% |
|  | (14 to 51) | (1 to 3.76) | (35 to 94) | (1.1 to 2.74) | (-0.28 to -0.23) |
| Latvia | 4 | 0.13 | 24 | 0.59 | 4.01% |
|  | (4 to 5) | (0.11 to 0.15) | (21 to 28) | (0.51 to 0.68) | (3.33 to 4.7) |
| Lebanon | 29 | 1.84 | 94 | 1.38 | -0.48% |
|  | (21 to 39) | (1.29 to 2.49) | (67 to 126) | (0.98 to 1.82) | (-0.69 to -0.27) |
| Lesotho | 8 | 1.21 | 19 | 2.51 | 3.28% |
|  | (4 to 14) | (0.63 to 2.1) | (12 to 27) | (1.55 to 3.56) | (2.89 to 3.67) |
| Liberia | 0 | 0.01 | 0 | 0.01 | 0.42% |
|  | (0 to 0) | (0 to 0.02) | (0 to 1) | (0 to 0.03) | (-0.1 to 0.95) |
| Libya | 11 | 0.68 | 37 | 0.91 | 1.27% |
|  | (6 to 23) | (0.37 to 1.37) | (23 to 52) | (0.56 to 1.29) | (0.99 to 1.54) |
| Lithuania | 1 | 0.03 | 10 | 0.16 | 4.16% |
|  | (1 to 2) | (0.03 to 0.04) | (8 to 11) | (0.14 to 0.19) | (3.37 to 4.96) |
| Luxembourg | 2 | 0.41 | 3 | 0.23 | -1.51% |
|  | (2 to 2) | (0.37 to 0.43) | (3 to 3) | (0.19 to 0.26) | (-1.9 to -1.12) |
| Madagascar | 76 | 1.72 | 145 | 1.59 | -0.28% |
|  | (35 to 183) | (0.73 to 4.28) | (72 to 301) | (0.8 to 3.26) | (-0.35 to -0.2) |
| Malawi | 66 | 2.06 | 130 | 2.09 | 0.18% |
|  | (38 to 105) | (1.15 to 3.38) | (80 to 193) | (1.33 to 3.09) | (-0.01 to 0.37) |
| Malaysia | 215 | 2.62 | 1274 | 5.62 | 2.09% |
|  | (163 to 282) | (1.95 to 3.52) | (895 to 1640) | (3.96 to 7.23) | (1.62 to 2.56) |
| Maldives | 1 | 2.23 | 3 | 1.16 | -2.76% |
|  | (1 to 2) | (1.2 to 3.52) | (2 to 4) | (0.74 to 1.62) | (-3.03 to -2.48) |
| Mali | 0 | 0.01 | 1 | 0.01 | 0.08% |
|  | (0 to 1) | (0 to 0.02) | (0 to 2) | (0 to 0.02) | (-0.42 to 0.58) |
| Malta | 11 | 3.04 | 39 | 3.28 | 0.42% |
|  | (10 to 12) | (2.71 to 3.28) | (31 to 44) | (2.67 to 3.72) | (-0.03 to 0.88) |
| Marshall Islands | 0 | 1.31 | 0 | 1.51 | 0.82% |
|  | (0 to 0) | (0.64 to 2.41) | (0 to 1) | (0.81 to 2.28) | (0.7 to 0.94) |
| Mauritania | 0 | 0.01 | 0 | 0.01 | 0.40% |
|  | (0 to 0) | (0 to 0.02) | (0 to 1) | (0 to 0.02) | (-0.14 to 0.93) |
| Mauritius | 3 | 0.52 | 31 | 1.87 | 4.11% |
|  | (3 to 3) | (0.48 to 0.55) | (27 to 33) | (1.65 to 2.02) | (3.65 to 4.57) |
| Mexico | 544 | 1.69 | 695 | 0.63 | -2.67% |
|  | (523 to 559) | (1.6 to 1.74) | (556 to 903) | (0.51 to 0.82) | (-2.96 to -2.37) |
| Micronesia (Federated States of) | 1 | 1.42 | 1 | 1.66 | 0.59% |
|  | (0 to 1) | (0.75 to 2.37) | (0 to 1) | (0.83 to 2.51) | (0.56 to 0.62) |
| Monaco | 0 | 0.17 | 0 | 0.18 | -0.42% |
|  | (0 to 0) | (0.11 to 0.22) | (0 to 0) | (0.12 to 0.24) | (-0.6 to -0.23) |
| Mongolia | 0 | 0.02 | 1 | 0.05 | 0.68% |
|  | (0 to 2) | (0 to 0.16) | (0 to 3) | (0.01 to 0.15) | (-0.06 to 1.42) |
| Montenegro | 0 | 0 | 0 | 0 | 0.87% |
|  | (0 to 0) | (0 to 0) | (0 to 0) | (0 to 0) | (0.42 to 1.32) |
| Morocco | 68 | 0.57 | 269 | 1.02 | 1.75% |
|  | (43 to 111) | (0.35 to 0.94) | (194 to 369) | (0.74 to 1.41) | (1.57 to 1.93) |
| Mozambique | 12 | 0.24 | 29 | 0.28 | 0.10% |
|  | (6 to 24) | (0.11 to 0.48) | (14 to 49) | (0.15 to 0.47) | (-0.5 to 0.69) |
| Myanmar | 297 | 1.75 | 628 | 1.69 | -0.22% |
|  | (150 to 491) | (0.96 to 3.02) | (396 to 857) | (1.08 to 2.29) | (-0.28 to -0.16) |
| Namibia | 6 | 1.5 | 19 | 2.04 | 1.54% |
|  | (4 to 9) | (0.99 to 2.34) | (13 to 27) | (1.38 to 2.92) | (1.18 to 1.9) |
| Nauru | 0 | 1.47 | 0 | 1.99 | 1.04% |
|  | (0 to 0) | (0.79 to 2.32) | (0 to 0) | (1.07 to 3.09) | (0.97 to 1.12) |
| Nepal | 2 | 0.02 | 9 | 0.04 | 1.02% |
|  | (1 to 2) | (0.02 to 0.03) | (6 to 13) | (0.03 to 0.05) | (0.61 to 1.43) |
| Netherlands | 350 | 1.75 | 249 | 0.6 | -2.66% |
|  | (295 to 382) | (1.47 to 1.9) | (198 to 286) | (0.48 to 0.69) | (-3.21 to -2.12) |
| New Zealand | 9 | 0.25 | 9 | 0.09 | -3.58% |
|  | (8 to 10) | (0.21 to 0.28) | (7 to 11) | (0.07 to 0.11) | (-4.51 to -2.63) |
| Nicaragua | 7 | 0.53 | 18 | 0.45 | -1.02% |
|  | (5 to 10) | (0.35 to 0.72) | (12 to 25) | (0.3 to 0.62) | (-1.19 to -0.85) |
| Niger | 0 | 0.01 | 1 | 0.01 | 0.29% |
|  | (0 to 0) | (0 to 0.02) | (0 to 2) | (0 to 0.02) | (-0.17 to 0.75) |
| Nigeria | 3 | 0.01 | 10 | 0.01 | 0.43% |
|  | (1 to 6) | (0 to 0.02) | (2 to 18) | (0 to 0.02) | (0.06 to 0.8) |
| Niue | 0 | 1.31 | 0 | 1.54 | 0.70% |
|  | (0 to 0) | (0.74 to 2.06) | (0 to 0) | (0.86 to 2.21) | (0.62 to 0.77) |
| North Macedonia | 0 | 0 | 0 | 0 | 4.21% |
|  | (0 to 0) | (0 to 0) | (0 to 0) | (0 to 0) | (3.73 to 4.69) |
| Northern Mariana Islands | 0 | 1.89 | 0 | 1.17 | -3.07% |
|  | (0 to 0) | (1.18 to 2.75) | (0 to 0) | (0.55 to 1.9) | (-3.71 to -2.43) |
| Norway | 25 | 0.32 | 21 | 0.16 | -0.74% |
|  | (22 to 27) | (0.28 to 0.34) | (17 to 24) | (0.13 to 0.18) | (-1.09 to -0.4) |
| Oman | 10 | 1.92 | 30 | 2.43 | 0.50% |
|  | (7 to 15) | (1.26 to 2.96) | (19 to 41) | (1.59 to 3.38) | (-0.01 to 1.01) |
| Pakistan | 105 | 0.18 | 189 | 0.16 | -0.06% |
|  | (45 to 193) | (0.07 to 0.35) | (64 to 354) | (0.05 to 0.31) | (-0.2 to 0.07) |
| Palau | 0 | 0.05 | 0 | 0.04 | -1.89% |
|  | (0 to 0) | (0.01 to 0.13) | (0 to 0) | (0.01 to 0.11) | (-2.61 to -1.17) |
| Palestine | 5 | 0.76 | 12 | 0.77 | -0.39% |
|  | (3 to 8) | (0.48 to 1.19) | (8 to 15) | (0.53 to 0.97) | (-0.6 to -0.17) |
| Panama | 13 | 0.97 | 55 | 1.17 | 0.94% |
|  | (12 to 15) | (0.83 to 1.09) | (42 to 66) | (0.89 to 1.41) | (0.79 to 1.1) |
| Papua New Guinea | 13 | 0.97 | 40 | 1.01 | 0.04% |
|  | (4 to 36) | (0.3 to 2.76) | (12 to 94) | (0.33 to 2.33) | (-0.03 to 0.12) |
| Paraguay | 8 | 0.4 | 28 | 0.53 | 0.35% |
|  | (4 to 16) | (0.21 to 0.78) | (17 to 42) | (0.32 to 0.79) | (0.05 to 0.64) |
| Peru | 91 | 0.83 | 102 | 0.3 | -3.88% |
|  | (62 to 116) | (0.58 to 1.06) | (51 to 175) | (0.15 to 0.52) | (-4.51 to -3.25) |
| Philippines | 488 | 2.46 | 1433 | 2.45 | -0.14% |
|  | (351 to 681) | (1.93 to 3.25) | (1110 to 1748) | (1.88 to 3) | (-0.25 to -0.02) |
| Poland | 15 | 0.04 | 0 | 0 | -16.54% |
|  | (14 to 16) | (0.03 to 0.04) | (0 to 0) | (0 to 0) | (-17.98 to -15.08) |
| Portugal | 51 | 0.44 | 74 | 0.22 | -1.73% |
|  | (47 to 55) | (0.4 to 0.47) | (60 to 85) | (0.18 to 0.25) | (-2.86 to -0.59) |
| Puerto Rico | 65 | 2.08 | 74 | 0.77 | -2.77% |
|  | (60 to 69) | (1.9 to 2.21) | (60 to 88) | (0.62 to 0.91) | (-3.16 to -2.38) |
| Qatar | 1 | 1.31 | 4 | 1.4 | -1.21% |
|  | (0 to 1) | (0.73 to 2.15) | (2 to 7) | (0.65 to 2.16) | (-1.64 to -0.77) |
| Republic of Korea | 160 | 0.95 | 434 | 0.49 | -2.23% |
|  | (118 to 196) | (0.7 to 1.2) | (319 to 550) | (0.36 to 0.62) | (-2.43 to -2.04) |
| Republic of Moldova | 0 | 0 | 1 | 0.01 | 4.10% |
|  | (0 to 0) | (0 to 0) | (1 to 1) | (0.01 to 0.01) | (3.31 to 4.89) |
| Romania | 6 | 0.02 | 43 | 0.11 | 4.14% |
|  | (5 to 7) | (0.02 to 0.03) | (37 to 49) | (0.09 to 0.12) | (3.17 to 5.13) |
| Russian Federation | 25 | 0.02 | 346 | 0.15 | 5.56% |
|  | (24 to 27) | (0.01 to 0.02) | (315 to 375) | (0.14 to 0.16) | (4.73 to 6.41) |
| Rwanda | 53 | 2.2 | 83 | 1.69 | -0.83% |
|  | (30 to 92) | (1.19 to 3.94) | (37 to 152) | (0.78 to 2.98) | (-1.09 to -0.58) |
| Saint Kitts and Nevis | 2 | 7.39 | 3 | 6.1 | -0.27% |
|  | (2 to 3) | (6.58 to 8.15) | (2 to 3) | (5.26 to 6.82) | (-0.5 to -0.04) |
| Saint Lucia | 3 | 4.73 | 11 | 5.08 | 0.37% |
|  | (3 to 3) | (4.35 to 5.15) | (9 to 13) | (4.22 to 5.97) | (-0.12 to 0.87) |
| Saint Vincent and the Grenadines | 1 | 1.68 | 7 | 6.14 | 4.59% |
|  | (1 to 1) | (1.55 to 1.8) | (7 to 9) | (5.38 to 7.04) | (3.7 to 5.48) |
| Samoa | 1 | 1.23 | 2 | 1.41 | 0.62% |
|  | (0 to 1) | (0.7 to 1.85) | (1 to 3) | (0.81 to 2.06) | (0.49 to 0.74) |
| San Marino | 0 | 0.09 | 0 | 0.04 | -2.17% |
|  | (0 to 0) | (0.06 to 0.13) | (0 to 0) | (0.02 to 0.06) | (-2.41 to -1.93) |
| Sao Tome and Principe | 0 | 0 | 0 | 0 | 2.20% |
|  | (0 to 0) | (0 to 0) | (0 to 0) | (0 to 0) | (1.95 to 2.46) |
| Saudi Arabia | 206 | 4.6 | 587 | 5.25 | 0.74% |
|  | (135 to 321) | (2.99 to 7.27) | (465 to 724) | (4.33 to 6.52) | (0.59 to 0.88) |
| Senegal | 0 | 0.01 | 1 | 0.01 | 0.28% |
|  | (0 to 1) | (0 to 0.02) | (0 to 2) | (0 to 0.02) | (-0.21 to 0.77) |
| Serbia | 10 | 0.12 | 28 | 0.17 | 0.80% |
|  | (6 to 17) | (0.07 to 0.2) | (12 to 43) | (0.07 to 0.25) | (0.42 to 1.19) |
| Seychelles | 0 | 0.74 | 1 | 0.88 | 0.67% |
|  | (0 to 1) | (0.46 to 1.05) | (0 to 1) | (0.52 to 1.15) | (0.48 to 0.86) |
| Sierra Leone | 0 | 0.01 | 0 | 0.01 | -0.42% |
|  | (0 to 0) | (0 to 0.02) | (0 to 1) | (0 to 0.02) | (-0.88 to 0.03) |
| Singapore | 23 | 1.46 | 60 | 0.74 | -3.23% |
|  | (21 to 25) | (1.32 to 1.57) | (48 to 69) | (0.59 to 0.85) | (-3.75 to -2.72) |
| Slovakia | 7 | 0.13 | 20 | 0.22 | 1.32% |
|  | (5 to 11) | (0.09 to 0.18) | (12 to 28) | (0.13 to 0.3) | (0.77 to 1.87) |
| Slovenia | 2 | 0.09 | 12 | 0.24 | 3.36% |
|  | (2 to 2) | (0.08 to 0.1) | (11 to 14) | (0.21 to 0.28) | (2.54 to 4.18) |
| Solomon Islands | 1 | 1.15 | 4 | 1.38 | 0.58% |
|  | (0 to 2) | (0.4 to 2.28) | (1 to 7) | (0.52 to 2.64) | (0.55 to 0.61) |
| Somalia | 48 | 2.3 | 113 | 2.17 | 0.05% |
|  | (20 to 107) | (0.95 to 5.39) | (59 to 216) | (1.12 to 4.57) | (-0.02 to 0.12) |
| South Africa | 263 | 1.58 | 540 | 1.61 | -0.36% |
|  | (150 to 375) | (0.91 to 2.3) | (382 to 684) | (1.13 to 2.01) | (-0.52 to -0.2) |
| South Sudan | 45 | 1.99 | 77 | 2.29 | 0.25% |
|  | (18 to 97) | (0.85 to 4.45) | (35 to 147) | (1.07 to 4.44) | (0.15 to 0.34) |
| Spain | 351 | 0.69 | 652 | 0.44 | 0.39% |
|  | (312 to 379) | (0.6 to 0.74) | (502 to 750) | (0.34 to 0.5) | (-0.32 to 1.1) |
| Sri Lanka | 24 | 0.31 | 89 | 0.4 | 0.06% |
|  | (15 to 35) | (0.2 to 0.45) | (49 to 136) | (0.22 to 0.6) | (-0.43 to 0.55) |
| Sudan | 43 | 0.58 | 148 | 1 | 1.66% |
|  | (25 to 77) | (0.31 to 1.07) | (79 to 278) | (0.54 to 1.89) | (1.47 to 1.85) |
| Suriname | 13 | 5.69 | 32 | 5.5 | -0.28% |
|  | (10 to 16) | (4.42 to 7.05) | (23 to 42) | (3.89 to 7.42) | (-0.49 to -0.08) |
| Sweden | 72 | 0.42 | 27 | 0.09 | -1.89% |
|  | (64 to 78) | (0.37 to 0.46) | (21 to 31) | (0.07 to 0.1) | (-3.35 to -0.41) |
| Switzerland | 17 | 0.15 | 22 | 0.08 | -1.32% |
|  | (15 to 19) | (0.13 to 0.16) | (17 to 27) | (0.06 to 0.1) | (-1.82 to -0.81) |
| Syrian Arab Republic | 1 | 0.03 | 2 | 0.03 | 0.11% |
|  | (1 to 2) | (0.02 to 0.04) | (1 to 3) | (0.02 to 0.03) | (-0.2 to 0.41) |
| Taiwan (Province of China) | 296 | 2.73 | 353 | 0.78 | -2.34% |
|  | (274 to 314) | (2.5 to 2.91) | (297 to 403) | (0.66 to 0.88) | (-3.21 to -1.47) |
| Tajikistan | 0 | 0 | 0 | 0 | -2.83% |
|  | (0 to 0) | (0 to 0) | (0 to 0) | (0 to 0) | (-3.32 to -2.34) |
| Thailand | 418 | 1.49 | 1846 | 1.72 | -0.11% |
|  | (217 to 725) | (0.77 to 2.49) | (923 to 2630) | (0.85 to 2.44) | (-0.66 to 0.44) |
| Timor-Leste | 3 | 1.42 | 10 | 1.52 | 0.06% |
|  | (1 to 5) | (0.68 to 2.7) | (5 to 16) | (0.83 to 2.4) | (-0.07 to 0.18) |
| Togo | 0 | 0.01 | 0 | 0.01 | 0.20% |
|  | (0 to 0) | (0 to 0.02) | (0 to 1) | (0 to 0.02) | (-0.2 to 0.59) |
| Tokelau | 0 | 1.22 | 0 | 1.27 | 0.10% |
|  | (0 to 0) | (0.64 to 2.09) | (0 to 0) | (0.67 to 1.9) | (0.06 to 0.15) |
| Tonga | 0 | 0.9 | 1 | 1.13 | 0.78% |
|  | (0 to 1) | (0.43 to 1.72) | (0 to 1) | (0.59 to 1.8) | (0.67 to 0.89) |
| Trinidad and Tobago | 16 | 2.63 | 22 | 1.21 | -2.47% |
|  | (14 to 17) | (2.37 to 2.95) | (17 to 26) | (0.96 to 1.47) | (-2.65 to -2.29) |
| Tunisia | 19 | 0.52 | 115 | 1.05 | 1.94% |
|  | (13 to 28) | (0.36 to 0.79) | (58 to 218) | (0.54 to 1.99) | (1.64 to 2.25) |
| Turkmenistan | 0 | 0 | 0 | 0 | -0.08% |
|  | (0 to 0) | (0 to 0) | (0 to 0) | (0 to 0) | (-0.36 to 0.21) |
| Tuvalu | 0 | 1.36 | 0 | 1.46 | 0.28% |
|  | (0 to 0) | (0.72 to 2.27) | (0 to 0) | (0.79 to 2.15) | (0.24 to 0.33) |
| Uganda | 91 | 1.65 | 220 | 1.73 | 0.08% |
|  | (36 to 237) | (0.64 to 4.36) | (111 to 404) | (0.94 to 3.1) | (-0.05 to 0.22) |
| Ukraine | 11 | 0.02 | 15 | 0.02 | 2.97% |
|  | (8 to 13) | (0.01 to 0.02) | (11 to 19) | (0.02 to 0.03) | (2.06 to 3.89) |
| United Arab Emirates | 1 | 0.64 | 4 | 0.47 | -0.32% |
|  | (1 to 2) | (0.31 to 1.08) | (2 to 7) | (0.23 to 0.75) | (-0.9 to 0.26) |
| United Kingdom | 516 | 0.55 | 172 | 0.11 | -4.65% |
|  | (468 to 542) | (0.49 to 0.58) | (145 to 186) | (0.09 to 0.12) | (-5.34 to -3.96) |
| United Republic of Tanzania | 188 | 2.06 | 401 | 1.82 | -0.39% |
|  | (85 to 414) | (0.97 to 4.6) | (217 to 820) | (0.99 to 3.54) | (-0.46 to -0.32) |
| United States of America | 1654 | 0.49 | 1411 | 0.22 | -2.24% |
|  | (1466 to 1762) | (0.43 to 0.52) | (1182 to 1626) | (0.19 to 0.26) | (-2.47 to -2.01) |
| United States Virgin Islands | 2 | 2.84 | 4 | 2.35 | -0.36% |
|  | (1 to 2) | (2.22 to 3.59) | (3 to 5) | (1.68 to 3.27) | (-0.55 to -0.17) |
| Uruguay | 65 | 1.7 | 156 | 2.26 | 1.35% |
|  | (57 to 71) | (1.5 to 1.87) | (134 to 172) | (1.97 to 2.46) | (0.93 to 1.78) |
| Uzbekistan | 0 | 0 | 0 | 0 | 1.55% |
|  | (0 to 0) | (0 to 0) | (0 to 0) | (0 to 0) | (1.28 to 1.82) |
| Vanuatu | 1 | 1.18 | 2 | 1.43 | 0.54% |
|  | (0 to 1) | (0.45 to 2.69) | (1 to 4) | (0.48 to 2.75) | (0.48 to 0.59) |
| Venezuela (Bolivarian Republic of) | 11 | 0.14 | 23 | 0.08 | -1.54% |
|  | (10 to 12) | (0.12 to 0.15) | (18 to 29) | (0.06 to 0.11) | (-1.9 to -1.19) |
| Viet Nam | 134 | 0.41 | 370 | 0.5 | -0.24% |
|  | (25 to 358) | (0.08 to 1.14) | (95 to 655) | (0.13 to 0.88) | (-0.81 to 0.34) |
| Yemen | 20 | 0.55 | 68 | 0.68 | 0.58% |
|  | (11 to 40) | (0.28 to 1.26) | (41 to 109) | (0.41 to 1.14) | (0.5 to 0.66) |
| Zambia | 59 | 2.28 | 126 | 2.12 | -0.52% |
|  | (36 to 82) | (1.46 to 3.27) | (72 to 196) | (1.27 to 3.3) | (-0.91 to -0.13) |
| Zimbabwe | 77 | 3.1 | 173 | 4.2 | 1.26% |
|  | (26 to 135) | (1.06 to 5.57) | (63 to 283) | (1.57 to 6.98) | (0.82 to 1.69) |

| Table S4: Age-standardized disability-adjusted life years (DALY) for decubitus ulcers in 1990 and 2021 and their temporal trends from 1990 to 2021 at the national and territorial levels. | | | | | |
| --- | --- | --- | --- | --- | --- |
| Location | Counts 1990  (95% UI) | Age-standardized DALY rate (95% UI) | Counts 2021 (95% UI) | Age-standardized DALY rate (95% UI) | EAPC (95% CI) |
| Afghanistan | 1450 | 22 | 2717 | 26.14 | 0.76% |
|  | (929 to 2182) | (13.74 to 34.45) | (1628 to 3787) | (16.39 to 36.78) | (0.65 to 0.86) |
| Albania | 49 | 2.34 | 100 | 2.52 | 0.39% |
|  | (35 to 64) | (1.71 to 3.09) | (69 to 135) | (1.77 to 3.41) | (0.32 to 0.46) |
| Algeria | 1211 | 10.63 | 4261 | 14.73 | 1.56% |
|  | (852 to 1713) | (7.7 to 14.91) | (2981 to 5622) | (10.66 to 19.17) | (1.34 to 1.77) |
| American Samoa | 19 | 77.49 | 44 | 93.5 | 1.28% |
|  | (9 to 32) | (36.51 to 126.11) | (22 to 77) | (49.84 to 161.93) | (0.73 to 1.84) |
| Andorra | 1 | 1.7 | 2 | 1.17 | -1.20% |
|  | (1 to 1) | (1.2 to 2.36) | (1 to 3) | (0.82 to 1.56) | (-1.39 to -1.02) |
| Angola | 1099 | 25.35 | 2620 | 21.41 | -2.16% |
|  | (399 to 1901) | (10.14 to 40.81) | (894 to 4851) | (8.51 to 38.26) | (-3.31 to -1) |
| Antigua and Barbuda | 40 | 70.86 | 104 | 108.79 | 1.40% |
|  | (36 to 45) | (63.45 to 79.46) | (95 to 112) | (99.7 to 116.96) | (0.97 to 1.84) |
| Argentina | 4271 | 14.03 | 18525 | 32.06 | 3.70% |
|  | (3794 to 4704) | (12.5 to 15.42) | (16554 to 20155) | (28.72 to 34.98) | (2.85 to 4.55) |
| Armenia | 18 | 0.66 | 69 | 1.59 | 3.65% |
|  | (11 to 24) | (0.42 to 0.85) | (52 to 89) | (1.2 to 2.05) | (2.6 to 4.72) |
| Australia | 731 | 3.98 | 904 | 1.86 | -3.60% |
|  | (654 to 803) | (3.56 to 4.38) | (760 to 1036) | (1.58 to 2.13) | (-4.22 to -2.98) |
| Austria | 358 | 3 | 332 | 1.64 | -2.40% |
|  | (319 to 399) | (2.65 to 3.36) | (281 to 381) | (1.39 to 1.92) | (-2.58 to -2.21) |
| Azerbaijan | 35 | 0.63 | 64 | 0.61 | 0.62% |
|  | (14 to 63) | (0.27 to 1.08) | (23 to 127) | (0.24 to 1.15) | (-0.1 to 1.35) |
| Bahamas | 203 | 125.03 | 474 | 126.51 | -0.02% |
|  | (188 to 221) | (115.04 to 136.39) | (388 to 577) | (104.35 to 152.93) | (-0.17 to 0.14) |
| Bahrain | 73 | 75.94 | 272 | 65.89 | -1.42% |
|  | (32 to 136) | (36.81 to 134.25) | (110 to 480) | (30.47 to 106.99) | (-2.12 to -0.72) |
| Bangladesh | 2468 | 6.5 | 7264 | 8.59 | 0.55% |
|  | (1382 to 3942) | (3.17 to 11.71) | (3965 to 11255) | (4.27 to 14.38) | (0.24 to 0.87) |
| Barbados | 807 | 269.76 | 979 | 196.36 | -1.08% |
|  | (726 to 879) | (243.8 to 292.65) | (798 to 1171) | (160.49 to 235.17) | (-1.28 to -0.88) |
| Belarus | 362 | 2.99 | 530 | 3.72 | 0.37% |
|  | (279 to 448) | (2.3 to 3.68) | (441 to 623) | (3.08 to 4.38) | (0.21 to 0.53) |
| Belgium | 4875 | 30.93 | 3000 | 10.54 | -3.96% |
|  | (4337 to 5299) | (27.58 to 33.53) | (2448 to 3404) | (8.87 to 11.83) | (-4.24 to -3.67) |
| Belize | 21 | 20.48 | 52 | 18.49 | -0.81% |
|  | (16 to 25) | (15.64 to 24.49) | (46 to 59) | (16.14 to 20.73) | (-1.27 to -0.34) |
| Benin | 8 | 0.32 | 48 | 0.54 | 2.49% |
|  | (5 to 12) | (0.18 to 0.5) | (16 to 100) | (0.19 to 1.02) | (2.08 to 2.91) |
| Bermuda | 15 | 24.92 | 33 | 23.04 | -0.49% |
|  | (13 to 17) | (21.41 to 28.3) | (28 to 39) | (19.5 to 27.63) | (-0.92 to -0.06) |
| Bhutan | 36 | 9.79 | 53 | 8.18 | -0.66% |
|  | (17 to 58) | (5.01 to 15.6) | (28 to 87) | (4.15 to 13.7) | (-0.73 to -0.59) |
| Bolivia (Plurinational State of) | 390 | 11.35 | 1013 | 12.09 | 0.20% |
|  | (254 to 538) | (7.99 to 15.35) | (654 to 1460) | (7.79 to 17.06) | (0.14 to 0.26) |
| Bosnia and Herzegovina | 189 | 4.75 | 248 | 4.34 | -0.61% |
|  | (135 to 246) | (3.39 to 6.14) | (173 to 330) | (3.02 to 5.76) | (-0.76 to -0.45) |
| Botswana | 126 | 25.13 | 356 | 28.29 | 0.19% |
|  | (79 to 193) | (15.83 to 39.81) | (243 to 520) | (19.71 to 40.67) | (-0.13 to 0.51) |
| Brazil | 12891 | 14.59 | 41247 | 16.98 | 0.83% |
|  | (12105 to 13714) | (13.6 to 15.49) | (36847 to 45951) | (15.16 to 18.94) | (0.68 to 0.98) |
| Brunei Darussalam | 24 | 25.35 | 53 | 22.08 | -0.02% |
|  | (19 to 31) | (19.45 to 31.96) | (37 to 71) | (15.99 to 28.7) | (-0.35 to 0.31) |
| Bulgaria | 189 | 1.92 | 285 | 2.19 | 0.75% |
|  | (128 to 263) | (1.34 to 2.6) | (210 to 376) | (1.61 to 2.87) | (0.63 to 0.88) |
| Burkina Faso | 155 | 3.81 | 444 | 4.28 | 2.09% |
|  | (25 to 380) | (0.6 to 8.76) | (52 to 1036) | (0.43 to 9.82) | (1.04 to 3.15) |
| Burundi | 1353 | 48.68 | 2181 | 37.03 | -1.33% |
|  | (702 to 2336) | (26.09 to 89.85) | (1023 to 3998) | (18.13 to 67.98) | (-1.49 to -1.17) |
| Cabo Verde | 1 | 0.45 | 9 | 2.19 | 5.11% |
|  | (1 to 2) | (0.29 to 0.73) | (5 to 15) | (1.02 to 3.62) | (4.47 to 5.75) |
| Cambodia | 1653 | 35.68 | 3673 | 33.57 | -0.25% |
|  | (829 to 2509) | (19.12 to 56.89) | (2017 to 5336) | (20.46 to 46.96) | (-0.3 to -0.2) |
| Cameroon | 18 | 0.35 | 147 | 0.64 | 2.83% |
|  | (11 to 28) | (0.18 to 0.58) | (43 to 296) | (0.22 to 1.21) | (2.37 to 3.3) |
| Canada | 2503 | 7.88 | 3428 | 4.86 | -1.75% |
|  | (2100 to 3002) | (6.64 to 9.45) | (2630 to 4325) | (3.66 to 6.21) | (-1.87 to -1.63) |
| Central African Republic | 368 | 30.75 | 660 | 28.05 | -0.45% |
|  | (137 to 629) | (12.52 to 51.68) | (230 to 1176) | (11.08 to 47.77) | (-0.53 to -0.37) |
| Chad | 10 | 0.3 | 52 | 0.52 | 2.50% |
|  | (6 to 16) | (0.18 to 0.48) | (21 to 89) | (0.25 to 0.85) | (2.14 to 2.85) |
| Chile | 650 | 7.12 | 4638 | 17.75 | 3.64% |
|  | (591 to 712) | (6.49 to 7.76) | (4081 to 5130) | (15.65 to 19.6) | (2.6 to 4.69) |
| China | 11965 | 1.75 | 61934 | 3.65 | 2.76% |
|  | (7821 to 21704) | (1.15 to 3.5) | (36313 to 77402) | (2.16 to 4.55) | (1.6 to 3.93) |
| Colombia | 7788 | 40.32 | 6060 | 10.85 | -3.26% |
|  | (7242 to 8303) | (37.6 to 43.14) | (4968 to 7280) | (8.87 to 13.07) | (-3.92 to -2.6) |
| Comoros | 95 | 42.16 | 198 | 38.57 | -0.70% |
|  | (45 to 173) | (20.77 to 74.9) | (96 to 341) | (19.05 to 66.47) | (-0.88 to -0.52) |
| Congo | 340 | 31.71 | 712 | 26.53 | -0.87% |
|  | (125 to 609) | (12.17 to 53.15) | (232 to 1250) | (9.12 to 46.3) | (-1.02 to -0.71) |
| Cook Islands | 1 | 7.79 | 2 | 8.44 | 0.89% |
|  | (1 to 1) | (4.74 to 12.04) | (1 to 3) | (4.07 to 14.2) | (0.61 to 1.16) |
| Costa Rica | 370 | 20.18 | 853 | 15.38 | -1.17% |
|  | (328 to 409) | (17.87 to 22.22) | (740 to 967) | (13.37 to 17.4) | (-1.38 to -0.95) |
| Croatia | 117 | 2.17 | 257 | 2.98 | 1.78% |
|  | (87 to 152) | (1.62 to 2.8) | (206 to 313) | (2.37 to 3.61) | (1.49 to 2.08) |
| Cuba | 1296 | 13.13 | 1387 | 6.91 | -2.49% |
|  | (1183 to 1424) | (11.96 to 14.41) | (1190 to 1585) | (5.94 to 7.9) | (-2.79 to -2.19) |
| Cyprus | 308 | 55.31 | 502 | 29.92 | -2.36% |
|  | (212 to 402) | (37.57 to 73.28) | (385 to 649) | (23.61 to 37.72) | (-2.62 to -2.09) |
| Czechia | 580 | 4.5 | 1842 | 8.83 | 4.09% |
|  | (490 to 681) | (3.81 to 5.27) | (1555 to 2143) | (7.47 to 10.25) | (3.35 to 4.84) |
| Democratic People's Republic of Korea | 951 | 7.12 | 1807 | 6.42 | -0.36% |
|  | (631 to 1394) | (4.85 to 10.01) | (1318 to 2521) | (4.76 to 8.76) | (-0.52 to -0.2) |
| Democratic Republic of the Congo | 4062 | 25.37 | 9592 | 25.66 | -0.04% |
|  | (1086 to 6929) | (7.04 to 41.76) | (3490 to 16245) | (9.43 to 42.89) | (-0.11 to 0.03) |
| Denmark | 214 | 2.46 | 140 | 1.01 | -4.13% |
|  | (193 to 231) | (2.24 to 2.66) | (117 to 161) | (0.85 to 1.16) | (-4.67 to -3.6) |
| Djibouti | 60 | 35.45 | 264 | 38.76 | 0.15% |
|  | (27 to 117) | (16.49 to 73.22) | (135 to 430) | (22.64 to 60.19) | (0.01 to 0.28) |
| Dominica | 69 | 118.28 | 94 | 123.55 | -0.33% |
|  | (53 to 92) | (90.65 to 156.82) | (74 to 120) | (97 to 157.42) | (-0.56 to -0.11) |
| Dominican Republic | 137 | 3.46 | 299 | 2.95 | -0.54% |
|  | (80 to 214) | (2.01 to 5.52) | (198 to 461) | (1.95 to 4.54) | (-0.78 to -0.29) |
| Ecuador | 154 | 2.71 | 1764 | 11.61 | 4.07% |
|  | (133 to 179) | (2.39 to 3.11) | (1408 to 2230) | (9.42 to 14.59) | (2.08 to 6.1) |
| Egypt | 4405 | 19.56 | 8952 | 17.43 | -0.25% |
|  | (3010 to 6992) | (12.74 to 35.21) | (6951 to 10812) | (13.86 to 20.89) | (-0.37 to -0.12) |
| El Salvador | 556 | 16.48 | 951 | 14.42 | -0.97% |
|  | (384 to 717) | (11.22 to 21.26) | (652 to 1271) | (9.73 to 19.49) | (-1.36 to -0.58) |
| Equatorial Guinea | 55 | 27.2 | 129 | 23.56 | -0.49% |
|  | (21 to 97) | (10.88 to 43.32) | (38 to 253) | (7.34 to 46.96) | (-0.66 to -0.31) |
| Eritrea | 742 | 51.85 | 1617 | 52.3 | 0.04% |
|  | (391 to 1300) | (29.14 to 94.72) | (982 to 2660) | (31.64 to 83.83) | (-0.04 to 0.12) |
| Estonia | 40 | 2.12 | 102 | 4.24 | 2.44% |
|  | (31 to 50) | (1.65 to 2.67) | (88 to 119) | (3.63 to 4.9) | (1.85 to 3.03) |
| Eswatini | 95 | 36.24 | 266 | 51.59 | 1.16% |
|  | (56 to 148) | (20.96 to 59.97) | (168 to 414) | (32.41 to 79.54) | (0.5 to 1.82) |
| Ethiopia | 11870 | 49 | 15246 | 28.4 | -2.25% |
|  | (6308 to 19389) | (28.32 to 81.95) | (8636 to 23079) | (16.97 to 41.59) | (-2.44 to -2.06) |
| Fiji | 49 | 13.84 | 101 | 16.7 | -0.14% |
|  | (26 to 86) | (7.92 to 22.97) | (49 to 158) | (9.5 to 24.09) | (-0.78 to 0.5) |
| Finland | 93 | 1.34 | 94 | 0.77 | -2.20% |
|  | (77 to 110) | (1.1 to 1.58) | (73 to 119) | (0.58 to 0.97) | (-2.75 to -1.66) |
| France | 22333 | 25.19 | 10639 | 5.63 | -5.76% |
|  | (19909 to 24011) | (22.65 to 26.91) | (8894 to 11931) | (4.83 to 6.26) | (-6.31 to -5.2) |
| Gabon | 161 | 28.55 | 257 | 26.06 | -0.51% |
|  | (58 to 258) | (10.76 to 45.65) | (77 to 492) | (7.82 to 48.07) | (-0.68 to -0.34) |
| Gambia | 2 | 0.31 | 10 | 0.6 | 2.86% |
|  | (1 to 2) | (0.17 to 0.49) | (3 to 20) | (0.21 to 1.1) | (2.45 to 3.27) |
| Georgia | 16 | 0.28 | 129 | 2.22 | 9.16% |
|  | (11 to 22) | (0.19 to 0.38) | (108 to 154) | (1.87 to 2.64) | (7.5 to 10.84) |
| Germany | 4477 | 3.41 | 8430 | 3.66 | 1.20% |
|  | (4030 to 4923) | (3.07 to 3.74) | (7101 to 9496) | (3.15 to 4.1) | (0.59 to 1.82) |
| Ghana | 184 | 3.03 | 709 | 2.77 | -0.56% |
|  | (33 to 390) | (0.49 to 6.05) | (80 to 1825) | (0.37 to 10.19) | (-0.85 to -0.26) |
| Greece | 155 | 1.09 | 348 | 1.08 | -0.89% |
|  | (142 to 167) | (0.99 to 1.17) | (298 to 391) | (0.94 to 1.2) | (-1.25 to -0.52) |
| Greenland | 2 | 6.13 | 3 | 4.74 | -0.71% |
|  | (1 to 2) | (4.27 to 8.2) | (2 to 4) | (3.38 to 6.32) | (-0.93 to -0.49) |
| Grenada | 90 | 116.61 | 116 | 122.48 | 0.04% |
|  | (78 to 100) | (100.63 to 130.43) | (102 to 130) | (107.11 to 135.46) | (-0.18 to 0.26) |
| Guam | 4 | 6.3 | 10 | 4.62 | 0.21% |
|  | (2 to 7) | (3.74 to 12.02) | (4 to 14) | (1.74 to 7.06) | (-0.65 to 1.07) |
| Guatemala | 1173 | 32.71 | 1300 | 11.95 | -4.02% |
|  | (1054 to 1317) | (29.26 to 35.96) | (1098 to 1503) | (10.21 to 13.79) | (-4.52 to -3.53) |
| Guinea | 11 | 0.31 | 43 | 0.49 | 2.19% |
|  | (7 to 19) | (0.17 to 0.53) | (18 to 76) | (0.22 to 0.79) | (1.82 to 2.57) |
| Guinea-Bissau | 2 | 0.38 | 9 | 0.59 | 2.50% |
|  | (1 to 3) | (0.2 to 0.68) | (4 to 15) | (0.28 to 0.94) | (2.02 to 2.98) |
| Guyana | 35 | 8.47 | 95 | 16.62 | 1.12% |
|  | (31 to 38) | (7.59 to 9.41) | (76 to 120) | (13.33 to 20.45) | (-0.03 to 2.29) |
| Haiti | 3116 | 89.68 | 6365 | 85.93 | 0.10% |
|  | (1965 to 4846) | (58.28 to 138.16) | (3925 to 10070) | (52.9 to 134.72) | (0.02 to 0.18) |
| Honduras | 376 | 16.38 | 1152 | 18.81 | 0.36% |
|  | (256 to 525) | (11.2 to 23.68) | (849 to 1489) | (14.08 to 23.83) | (0.21 to 0.51) |
| Hungary | 339 | 2.58 | 862 | 4.54 | 3.17% |
|  | (266 to 434) | (2.03 to 3.28) | (720 to 1005) | (3.77 to 5.28) | (2.66 to 3.7) |
| Iceland | 2 | 0.73 | 2 | 0.29 | -3.74% |
|  | (2 to 2) | (0.65 to 0.81) | (2 to 2) | (0.25 to 0.34) | (-4.08 to -3.4) |
| India | 80577 | 13.88 | 146565 | 11.95 | -1.48% |
|  | (48966 to 114443) | (8.06 to 19.71) | (90927 to 191410) | (7.19 to 15.59) | (-2.8 to -0.13) |
| Indonesia | 7434 | 6.96 | 24498 | 11.97 | 0.95% |
|  | (3854 to 13639) | (3.85 to 12.7) | (11178 to 35082) | (5.95 to 16.28) | (0.61 to 1.3) |
| Iran (Islamic Republic of) | 3016 | 12.51 | 8095 | 11.41 | -0.48% |
|  | (1989 to 3936) | (8.62 to 16.98) | (5972 to 9326) | (8.65 to 13.12) | (-0.68 to -0.27) |
| Iraq | 311 | 3.63 | 699 | 3.36 | -1.27% |
|  | (183 to 427) | (2.09 to 5.06) | (443 to 939) | (2.19 to 4.58) | (-1.71 to -0.84) |
| Ireland | 465 | 11.63 | 241 | 2.83 | -5.48% |
|  | (434 to 495) | (10.79 to 12.39) | (196 to 279) | (2.32 to 3.28) | (-5.96 to -4.99) |
| Israel | 2010 | 44.3 | 5983 | 43.52 | 0.11% |
|  | (1830 to 2178) | (40.43 to 48.04) | (5005 to 6647) | (36.86 to 48.26) | (-0.51 to 0.74) |
| Italy | 3544 | 4.23 | 8447 | 4.64 | -0.03% |
|  | (3226 to 3873) | (3.83 to 4.66) | (7153 to 9387) | (4 to 5.19) | (-0.36 to 0.31) |
| Jamaica | 1354 | 73.32 | 2606 | 79.99 | -0.07% |
|  | (1138 to 1514) | (61.81 to 81.63) | (2071 to 3197) | (63.57 to 98.29) | (-0.38 to 0.24) |
| Japan | 5952 | 4.13 | 13636 | 3.53 | -0.55% |
|  | (4988 to 7004) | (3.44 to 4.86) | (11483 to 15939) | (2.83 to 4.26) | (-0.97 to -0.13) |
| Jordan | 213 | 16.95 | 899 | 15.18 | -0.37% |
|  | (169 to 269) | (13.62 to 21.8) | (688 to 1117) | (12.05 to 18.82) | (-0.53 to -0.22) |
| Kazakhstan | 68 | 0.56 | 130 | 0.8 | 0.82% |
|  | (54 to 86) | (0.44 to 0.69) | (104 to 161) | (0.65 to 0.98) | (0.36 to 1.28) |
| Kenya | 3333 | 34.17 | 10152 | 41 | 0.84% |
|  | (1556 to 8272) | (15.41 to 89.25) | (5812 to 17000) | (24.98 to 70.01) | (0.74 to 0.93) |
| Kiribati | 41 | 104.18 | 79 | 108.65 | 0.08% |
|  | (16 to 89) | (43.22 to 227.6) | (36 to 153) | (52.7 to 206.23) | (-0.03 to 0.18) |
| Kuwait | 17 | 2.45 | 102 | 4.06 | 2.81% |
|  | (14 to 20) | (2.1 to 2.88) | (83 to 125) | (3.21 to 5.01) | (1.29 to 4.36) |
| Kyrgyzstan | 6 | 0.21 | 15 | 0.32 | 1.37% |
|  | (4 to 8) | (0.14 to 0.29) | (12 to 20) | (0.24 to 0.41) | (1 to 1.75) |
| Lao People's Democratic Republic | 803 | 39.01 | 1551 | 34.18 | -0.41% |
|  | (382 to 1322) | (19.78 to 69.33) | (742 to 2515) | (18.45 to 52.13) | (-0.44 to -0.39) |
| Latvia | 150 | 4.62 | 518 | 15.11 | 4.28% |
|  | (128 to 176) | (3.92 to 5.41) | (449 to 592) | (13.09 to 17.41) | (3.67 to 4.9) |
| Lebanon | 561 | 29.73 | 1286 | 19.58 | -1.50% |
|  | (389 to 751) | (20.86 to 39.85) | (939 to 1658) | (14.38 to 24.96) | (-1.75 to -1.26) |
| Lesotho | 168 | 21.26 | 438 | 44.99 | 2.77% |
|  | (91 to 275) | (11.65 to 35.21) | (279 to 631) | (29.13 to 63.62) | (2.18 to 3.36) |
| Liberia | 5 | 0.32 | 26 | 0.59 | 2.95% |
|  | (3 to 7) | (0.18 to 0.5) | (7 to 56) | (0.2 to 1.19) | (2.43 to 3.46) |
| Libya | 256 | 12.75 | 762 | 15.58 | 1.32% |
|  | (162 to 469) | (7.7 to 24.61) | (488 to 1070) | (9.93 to 21.88) | (1.04 to 1.61) |
| Lithuania | 77 | 1.81 | 227 | 4.57 | 3.54% |
|  | (60 to 97) | (1.4 to 2.29) | (197 to 265) | (3.92 to 5.35) | (3 to 4.09) |
| Luxembourg | 31 | 5.87 | 36 | 2.9 | -3.01% |
|  | (29 to 33) | (5.44 to 6.22) | (31 to 41) | (2.51 to 3.28) | (-3.38 to -2.64) |
| Madagascar | 2255 | 37.9 | 4764 | 34.53 | -0.32% |
|  | (1121 to 4953) | (17.34 to 91.4) | (2366 to 9282) | (17.31 to 71.84) | (-0.39 to -0.25) |
| Malawi | 2047 | 44.92 | 3903 | 43.69 | -0.45% |
|  | (1170 to 3165) | (26.61 to 72.22) | (2270 to 5860) | (26.81 to 63.92) | (-0.76 to -0.14) |
| Malaysia | 4873 | 51.19 | 25344 | 96.95 | 2.77% |
|  | (3736 to 6127) | (39.07 to 65.54) | (17725 to 32552) | (67.07 to 123.65) | (2.17 to 3.38) |
| Maldives | 34 | 40.65 | 57 | 17.95 | -3.94% |
|  | (21 to 47) | (23.64 to 59.94) | (34 to 81) | (11.22 to 24.97) | (-4.44 to -3.44) |
| Mali | 15 | 0.33 | 73 | 0.52 | 2.37% |
|  | (9 to 23) | (0.17 to 0.54) | (28 to 139) | (0.21 to 0.93) | (1.92 to 2.82) |
| Malta | 186 | 46.58 | 504 | 46.08 | -1% |
|  | (171 to 197) | (42.6 to 49.58) | (419 to 565) | (39.04 to 51.56) | (-1.49 to -0.51) |
| Marshall Islands | 5 | 27.13 | 12 | 32.74 | 0.72% |
|  | (2 to 8) | (13.14 to 48.01) | (6 to 19) | (16.13 to 50.11) | (0.56 to 0.89) |
| Mauritania | 4 | 0.31 | 15 | 0.49 | 2.17% |
|  | (2 to 6) | (0.18 to 0.49) | (6 to 29) | (0.19 to 0.94) | (1.77 to 2.58) |
| Mauritius | 79 | 10.74 | 547 | 32.16 | 4.67% |
|  | (74 to 85) | (10.08 to 11.48) | (499 to 582) | (29.29 to 34.16) | (4.17 to 5.17) |
| Mexico | 13206 | 30.99 | 16520 | 13.84 | -2.70% |
|  | (12599 to 13850) | (29.73 to 32.47) | (13266 to 20650) | (11.21 to 17.23) | (-3.04 to -2.36) |
| Micronesia (Federated States of) | 15 | 29.98 | 25 | 34.25 | 0.51% |
|  | (7 to 26) | (14.3 to 51.44) | (10 to 41) | (15.68 to 54.43) | (0.45 to 0.57) |
| Monaco | 2 | 2.8 | 3 | 2.74 | -0.73% |
|  | (1 to 3) | (1.9 to 3.6) | (2 to 4) | (1.93 to 3.7) | (-1.1 to -0.35) |
| Mongolia | 10 | 0.8 | 27 | 1.21 | 0.16% |
|  | (2 to 51) | (0.18 to 4) | (8 to 69) | (0.37 to 3) | (-0.7 to 1.03) |
| Montenegro | 11 | 1.86 | 15 | 1.74 | -0.21% |
|  | (7 to 15) | (1.24 to 2.54) | (10 to 21) | (1.19 to 2.42) | (-0.25 to -0.16) |
| Morocco | 1629 | 10.99 | 5247 | 17.09 | 1.74% |
|  | (1111 to 2455) | (7.29 to 17.04) | (3688 to 6983) | (12.19 to 22.7) | (1.61 to 1.87) |
| Mozambique | 398 | 5.52 | 961 | 6.62 | 1.28% |
|  | (170 to 721) | (2.58 to 10.33) | (431 to 1650) | (3.31 to 11.24) | (0.27 to 2.29) |
| Myanmar | 7917 | 34.07 | 13178 | 29.75 | -0.64% |
|  | (3920 to 12296) | (17.68 to 55.59) | (7704 to 18332) | (17.98 to 40.65) | (-0.75 to -0.54) |
| Namibia | 148 | 26.17 | 418 | 34.45 | 0.49% |
|  | (93 to 223) | (17.15 to 39.1) | (282 to 592) | (23.63 to 49) | (0.07 to 0.91) |
| Nauru | 2 | 33.17 | 3 | 42.39 | 0.83% |
|  | (1 to 3) | (17.04 to 54.45) | (1 to 4) | (21.23 to 66.8) | (0.66 to 1) |
| Nepal | 120 | 0.89 | 530 | 1.71 | 3.02% |
|  | (94 to 152) | (0.71 to 1.12) | (368 to 739) | (1.24 to 2.33) | (2.44 to 3.6) |
| Netherlands | 4512 | 22.16 | 2963 | 7.43 | -4.51% |
|  | (3942 to 4857) | (19.34 to 23.82) | (2466 to 3364) | (6.22 to 8.42) | (-4.87 to -4.15) |
| New Zealand | 168 | 4.45 | 198 | 2.38 | -2.16% |
|  | (148 to 188) | (3.92 to 5) | (162 to 236) | (1.9 to 2.89) | (-2.86 to -1.46) |
| Nicaragua | 248 | 13.29 | 527 | 10.82 | -0.93% |
|  | (173 to 305) | (9.44 to 16.65) | (374 to 669) | (7.83 to 13.62) | (-1.1 to -0.76) |
| Niger | 11 | 0.3 | 63 | 0.46 | 2.03% |
|  | (7 to 17) | (0.17 to 0.51) | (26 to 124) | (0.19 to 0.88) | (1.67 to 2.39) |
| Nigeria | 160 | 0.3 | 678 | 0.46 | 2.06% |
|  | (109 to 227) | (0.2 to 0.44) | (322 to 1079) | (0.22 to 0.7) | (1.72 to 2.41) |
| Niue | 1 | 24.68 | 1 | 29.68 | 0.40% |
|  | (0 to 1) | (13.09 to 39.1) | (0 to 1) | (14.68 to 44.98) | (0.34 to 0.47) |
| North Macedonia | 31 | 1.81 | 47 | 1.74 | -0.13% |
|  | (21 to 43) | (1.24 to 2.48) | (32 to 65) | (1.19 to 2.36) | (-0.17 to -0.08) |
| Northern Mariana Islands | 7 | 33.97 | 6 | 16.99 | -3.35% |
|  | (4 to 11) | (20.84 to 52.39) | (3 to 10) | (8.24 to 27.14) | (-4.51 to -2.17) |
| Norway | 407 | 5.66 | 342 | 3.26 | -0.85% |
|  | (369 to 446) | (5.05 to 6.28) | (291 to 395) | (2.74 to 3.84) | (-1.39 to -0.31) |
| Oman | 258 | 35.97 | 740 | 40.16 | 0.70% |
|  | (169 to 376) | (23.67 to 53.81) | (415 to 1023) | (25.7 to 54.16) | (-0.12 to 1.54) |
| Pakistan | 4206 | 5.2 | 7596 | 4.44 | -0.71% |
|  | (2258 to 6566) | (2.48 to 8.8) | (3664 to 12686) | (1.78 to 7.89) | (-0.84 to -0.58) |
| Palau | 0 | 0.89 | 0 | 0.82 | -2.23% |
|  | (0 to 0) | (0.32 to 2.11) | (0 to 0) | (0.3 to 1.94) | (-3.05 to -1.41) |
| Palestine | 98 | 12.05 | 249 | 11.86 | -0.29% |
|  | (64 to 146) | (7.86 to 18.3) | (160 to 311) | (8.02 to 14.52) | (-0.68 to 0.09) |
| Panama | 350 | 22 | 1066 | 23.5 | 0.18% |
|  | (307 to 392) | (19.31 to 24.68) | (861 to 1252) | (18.95 to 27.64) | (-0.01 to 0.38) |
| Papua New Guinea | 414 | 21.17 | 1236 | 21.85 | 0.25% |
|  | (125 to 1124) | (6.54 to 58.05) | (342 to 2958) | (6.7 to 50.7) | (0.21 to 0.3) |
| Paraguay | 209 | 8.85 | 624 | 10.81 | 0.76% |
|  | (130 to 378) | (5.42 to 15.98) | (398 to 867) | (6.92 to 15.04) | (0.52 to 1) |
| Peru | 2143 | 16.17 | 1977 | 5.78 | -5.10% |
|  | (1439 to 2690) | (11.34 to 20.17) | (1236 to 3000) | (3.59 to 8.88) | (-5.85 to -4.34) |
| Philippines | 13018 | 42.58 | 29005 | 40.07 | -0.22% |
|  | (8843 to 17889) | (30.97 to 57.68) | (21263 to 35758) | (30.5 to 48.6) | (-0.29 to -0.15) |
| Poland | 1164 | 2.86 | 1817 | 2.59 | -0.09% |
|  | (912 to 1477) | (2.24 to 3.63) | (1293 to 2429) | (1.84 to 3.44) | (-0.38 to 0.21) |
| Portugal | 797 | 6.29 | 871 | 2.82 | -4.86% |
|  | (735 to 847) | (5.79 to 6.67) | (738 to 984) | (2.44 to 3.16) | (-6.05 to -3.66) |
| Puerto Rico | 1136 | 33.53 | 1096 | 13.83 | -3.48% |
|  | (1066 to 1200) | (31.36 to 35.45) | (914 to 1280) | (11.56 to 16.09) | (-3.87 to -3.09) |
| Qatar | 19 | 19.47 | 129 | 19.6 | -0.57% |
|  | (13 to 27) | (11.78 to 30.21) | (77 to 191) | (9.68 to 30.39) | (-1.22 to 0.09) |
| Republic of Korea | 3846 | 16.08 | 7326 | 8.53 | -2.22% |
|  | (2947 to 4545) | (12.3 to 19.25) | (5684 to 9008) | (6.72 to 10.41) | (-2.46 to -1.99) |
| Republic of Moldova | 50 | 1.22 | 75 | 1.4 | 0.52% |
|  | (34 to 67) | (0.83 to 1.64) | (55 to 98) | (1.03 to 1.84) | (0.42 to 0.61) |
| Romania | 585 | 2.45 | 1377 | 3.89 | 2.36% |
|  | (444 to 752) | (1.88 to 3.14) | (1157 to 1592) | (3.27 to 4.54) | (2.03 to 2.69) |
| Russian Federation | 2523 | 1.55 | 9220 | 4.34 | 3.30% |
|  | (1931 to 3167) | (1.19 to 1.95) | (8437 to 10217) | (3.97 to 4.8) | (2.74 to 3.86) |
| Rwanda | 1741 | 50.23 | 2343 | 34.16 | -2.04% |
|  | (964 to 2938) | (28.82 to 86.53) | (1017 to 4408) | (15.49 to 61.78) | (-2.34 to -1.74) |
| Saint Kitts and Nevis | 47 | 130.09 | 52 | 95.14 | -1.22% |
|  | (41 to 52) | (115.28 to 144.56) | (43 to 61) | (81.34 to 109.69) | (-1.46 to -0.98) |
| Saint Lucia | 62 | 76.74 | 176 | 78.15 | -0.86% |
|  | (57 to 67) | (71.47 to 83.87) | (148 to 207) | (65.82 to 91.66) | (-1.39 to -0.33) |
| Saint Vincent and the Grenadines | 23 | 31.36 | 138 | 106.23 | 2.92% |
|  | (21 to 24) | (29.36 to 33.39) | (122 to 156) | (93.62 to 120.25) | (1.6 to 4.26) |
| Samoa | 20 | 23.79 | 40 | 28.34 | 0.54% |
|  | (10 to 33) | (12.53 to 38.41) | (18 to 63) | (13.69 to 43.19) | (0.46 to 0.61) |
| San Marino | 1 | 1.43 | 1 | 0.77 | -1.77% |
|  | (0 to 1) | (0.99 to 1.85) | (0 to 1) | (0.5 to 1.08) | (-2.09 to -1.45) |
| Sao Tome and Principe | 0 | 0.16 | 0 | 0.19 | 0.52% |
|  | (0 to 0) | (0.11 to 0.22) | (0 to 0) | (0.13 to 0.24) | (0.46 to 0.57) |
| Saudi Arabia | 5207 | 82.85 | 16618 | 89.3 | 0.29% |
|  | (3391 to 8165) | (54.19 to 128.88) | (12972 to 21102) | (72.92 to 109.7) | (0.12 to 0.45) |
| Senegal | 13 | 0.33 | 60 | 0.54 | 2.40% |
|  | (8 to 20) | (0.18 to 0.52) | (23 to 107) | (0.22 to 0.91) | (1.97 to 2.83) |
| Serbia | 327 | 3.49 | 696 | 4.36 | 1.25% |
|  | (232 to 486) | (2.44 to 5.06) | (409 to 935) | (2.57 to 5.79) | (0.89 to 1.62) |
| Seychelles | 8 | 13.53 | 15 | 14.12 | 0.26% |
|  | (4 to 11) | (7.69 to 18.74) | (8 to 19) | (7.76 to 18.41) | (-0.11 to 0.62) |
| Sierra Leone | 8 | 0.3 | 28 | 0.46 | 1.94% |
|  | (5 to 11) | (0.18 to 0.46) | (13 to 52) | (0.22 to 0.8) | (1.58 to 2.29) |
| Singapore | 486 | 24.96 | 919 | 11.48 | -1.99% |
|  | (455 to 518) | (23.24 to 26.56) | (790 to 1061) | (9.94 to 13.19) | (-2.3 to -1.67) |
| Slovakia | 283 | 4.95 | 583 | 6.69 | 1.64% |
|  | (221 to 373) | (3.84 to 6.49) | (397 to 751) | (4.49 to 8.58) | (1.42 to 1.87) |
| Slovenia | 83 | 3.53 | 277 | 6.19 | 3.08% |
|  | (69 to 98) | (2.91 to 4.19) | (240 to 319) | (5.38 to 7.13) | (2.62 to 3.54) |
| Solomon Islands | 36 | 25.14 | 123 | 31.03 | 0.73% |
|  | (11 to 75) | (8.38 to 51.06) | (37 to 230) | (10.41 to 57.2) | (0.68 to 0.78) |
| Somalia | 1649 | 52.03 | 3906 | 48.84 | -0.21% |
|  | (692 to 3367) | (21.92 to 118.66) | (1933 to 7371) | (25.73 to 97.73) | (-0.3 to -0.11) |
| South Africa | 5381 | 26.4 | 10016 | 25.06 | -0.69% |
|  | (3052 to 7312) | (15.16 to 36.84) | (6977 to 12561) | (17.95 to 31.41) | (-0.94 to -0.43) |
| South Sudan | 1250 | 43.03 | 2375 | 50.09 | 0.32% |
|  | (529 to 2588) | (18.35 to 91.49) | (1046 to 4502) | (22.78 to 96.43) | (0.13 to 0.52) |
| Spain | 5047 | 9.54 | 7172 | 5.3 | -2.17% |
|  | (4578 to 5368) | (8.63 to 10.16) | (5784 to 8182) | (4.39 to 5.99) | (-2.6 to -1.74) |
| Sri Lanka | 553 | 5.46 | 1578 | 6.54 | 1.08% |
|  | (346 to 831) | (3.51 to 8.07) | (852 to 2407) | (3.65 to 9.79) | (0.62 to 1.55) |
| Sudan | 1205 | 11.72 | 3514 | 17.63 | 1.70% |
|  | (744 to 1952) | (6.89 to 20.13) | (1828 to 6394) | (9.73 to 32.36) | (1.55 to 1.84) |
| Suriname | 303 | 113.9 | 657 | 108.46 | -0.13% |
|  | (224 to 368) | (86.6 to 138.62) | (467 to 881) | (78.05 to 144.97) | (-0.53 to 0.26) |
| Sweden | 1034 | 6.27 | 399 | 1.63 | -4.86% |
|  | (945 to 1113) | (5.74 to 6.75) | (336 to 458) | (1.37 to 1.89) | (-5.51 to -4.21) |
| Switzerland | 273 | 2.48 | 301 | 1.34 | -2.62% |
|  | (244 to 301) | (2.23 to 2.73) | (247 to 352) | (1.13 to 1.56) | (-3.08 to -2.15) |
| Syrian Arab Republic | 61 | 0.96 | 97 | 0.81 | -0.65% |
|  | (46 to 77) | (0.73 to 1.21) | (74 to 124) | (0.64 to 1.01) | (-0.75 to -0.55) |
| Taiwan (Province of China) | 6191 | 45.27 | 5012 | 11.64 | -5.36% |
|  | (5822 to 6514) | (41.93 to 47.86) | (4349 to 5655) | (10.15 to 13.09) | (-5.76 to -4.96) |
| Tajikistan | 7 | 0.24 | 12 | 0.21 | -0.58% |
|  | (5 to 10) | (0.16 to 0.33) | (8 to 17) | (0.14 to 0.29) | (-0.65 to -0.51) |
| Thailand | 10233 | 28.38 | 31721 | 30.89 | 1.02% |
|  | (5080 to 18794) | (14.7 to 50.98) | (14616 to 46117) | (14.13 to 44.92) | (0.35 to 1.7) |
| Timor-Leste | 80 | 26.09 | 232 | 27.75 | 0.38% |
|  | (34 to 145) | (12.57 to 46.6) | (101 to 396) | (13.4 to 45.97) | (0.21 to 0.55) |
| Togo | 5 | 0.31 | 30 | 0.48 | 2.14% |
|  | (3 to 8) | (0.18 to 0.49) | (14 to 50) | (0.24 to 0.75) | (1.76 to 2.52) |
| Tokelau | 0 | 24.75 | 0 | 26.25 | 0.01% |
|  | (0 to 1) | (12.5 to 41.71) | (0 to 1) | (13.75 to 40.63) | (-0.06 to 0.09) |
| Tonga | 10 | 18 | 17 | 21.34 | 0.68% |
|  | (4 to 18) | (7.97 to 34.06) | (8 to 30) | (9.76 to 36.7) | (0.54 to 0.83) |
| Trinidad and Tobago | 298 | 40.83 | 369 | 20.28 | -2.63% |
|  | (276 to 321) | (37.6 to 44.44) | (297 to 454) | (16.36 to 24.87) | (-2.84 to -2.42) |
| Tunisia | 453 | 9.5 | 2017 | 16.82 | 2.31% |
|  | (319 to 648) | (6.63 to 13.98) | (991 to 3829) | (8.46 to 31.5) | (2.13 to 2.49) |
| Turkmenistan | 5 | 0.26 | 10 | 0.26 | 0.05% |
|  | (4 to 7) | (0.19 to 0.34) | (8 to 14) | (0.19 to 0.34) | (-0.03 to 0.12) |
| Tuvalu | 2 | 28.22 | 3 | 29.42 | 0.19% |
|  | (1 to 3) | (14.06 to 45.77) | (1 to 5) | (13.94 to 44.36) | (0.12 to 0.25) |
| Uganda | 2600 | 34.61 | 6539 | 36.01 | -0.30% |
|  | (1079 to 6582) | (14.17 to 91.23) | (2979 to 12245) | (17.97 to 65.78) | (-0.5 to -0.09) |
| Ukraine | 954 | 1.48 | 1072 | 1.67 | -0.03% |
|  | (720 to 1233) | (1.11 to 1.92) | (823 to 1377) | (1.29 to 2.12) | (-0.3 to 0.24) |
| United Arab Emirates | 40 | 10.43 | 148 | 7.15 | -0.69% |
|  | (22 to 57) | (5.43 to 16.1) | (89 to 218) | (3.55 to 11.43) | (-1.52 to 0.15) |
| United Kingdom | 8133 | 8.84 | 3646 | 2.72 | -4.87% |
|  | (7566 to 8542) | (8.23 to 9.33) | (3124 to 4210) | (2.3 to 3.18) | (-5.35 to -4.4) |
| United Republic of Tanzania | 5478 | 43.99 | 11051 | 37.76 | -0.65% |
|  | (2544 to 12141) | (20.64 to 97.83) | (5692 to 23119) | (20.61 to 76.94) | (-0.71 to -0.58) |
| United States of America | 41907 | 12.85 | 50615 | 8.7 | -1.10% |
|  | (37110 to 47619) | (11.32 to 14.6) | (41961 to 59890) | (7.22 to 10.3) | (-1.19 to -1) |
| United States Virgin Islands | 37 | 49.52 | 63 | 41.5 | -0.51% |
|  | (27 to 46) | (37.92 to 61.92) | (45 to 88) | (29.43 to 58.76) | (-0.74 to -0.28) |
| Uruguay | 1013 | 26.14 | 2164 | 34.98 | 1.08% |
|  | (905 to 1108) | (23.32 to 28.66) | (1916 to 2347) | (31.52 to 37.94) | (0.38 to 1.78) |
| Uzbekistan | 25 | 0.2 | 47 | 0.2 | -0.03% |
|  | (17 to 36) | (0.14 to 0.28) | (31 to 65) | (0.13 to 0.28) | (-0.07 to 0.01) |
| Vanuatu | 17 | 25.05 | 54 | 30.06 | 0.51% |
|  | (6 to 38) | (9.03 to 57.12) | (14 to 116) | (9.21 to 61.83) | (0.45 to 0.57) |
| Venezuela (Bolivarian Republic of) | 613 | 5.53 | 1261 | 4.39 | -1.02% |
|  | (490 to 759) | (4.55 to 6.75) | (958 to 1572) | (3.35 to 5.46) | (-1.17 to -0.87) |
| Viet Nam | 2535 | 6.9 | 6352 | 7.5 | -0.12% |
|  | (575 to 6301) | (1.46 to 17.71) | (1739 to 10372) | (2.07 to 12.56) | (-1.05 to 0.83) |
| Yemen | 565 | 10.74 | 1690 | 12.1 | 0.41% |
|  | (337 to 998) | (6 to 22.18) | (1038 to 2633) | (7.44 to 19.15) | (0.31 to 0.52) |
| Zambia | 1874 | 51.64 | 3843 | 45.13 | -1.48% |
|  | (1136 to 2770) | (32.26 to 72.65) | (2171 to 6171) | (26.12 to 71.18) | (-2.13 to -0.82) |
| Zimbabwe | 1541 | 47.05 | 3968 | 69.25 | 2.37% |
|  | (511 to 2629) | (15.93 to 81.94) | (1338 to 6305) | (26.02 to 111.55) | (1.74 to 3.01) |

| Table S5: Age-standardized years lived with disability (YLD) for decubitus ulcers in 1990 and 2021 and their temporal trends from 1990 to 2021 at global, SDI quintile and GBD region levels. | | | | | |
| --- | --- | --- | --- | --- | --- |
| Location | Counts 1990  (95% UI) | Age-standardized YLD rate (95% UI) | Counts 2021 (95% UI) | Age-standardized YLD rate (95% UI) | EAPC (95% CI) |
| Global | 48905 | 1.3 | 101927 | 1.24 | -0.04% |
|  | (33686 to 65403) | (0.9 to 1.75) | (71278 to 135394) | (0.87 to 1.66) | (-0.12 to 0.04) |
| SDI quintile |  |  |  |  |  |
| High SDI | 24574 | 2.33 | 45609 | 2.23 | -0.22% |
|  | (16998 to 32996) | (1.61 to 3.12) | (32012 to 60791) | (1.55 to 2.96) | (-0.26 to -0.18) |
| High-middle SDI | 8740 | 0.91 | 16386 | 0.92 | 0.19% |
|  | (5928 to 11699) | (0.62 to 1.22) | (11308 to 21754) | (0.64 to 1.21) | (0.06 to 0.31) |
| Middle SDI | 10564 | 0.93 | 26933 | 1.1 | 0.78% |
|  | (7199 to 14163) | (0.64 to 1.25) | (18775 to 35980) | (0.77 to 1.48) | (0.65 to 0.91) |
| Low-middle SDI | 4121 | 0.53 | 10727 | 0.68 | 1.11% |
|  | (2706 to 5630) | (0.36 to 0.72) | (7117 to 14470) | (0.46 to 0.91) | (0.94 to 1.29) |
| Low SDI | 846 | 0.26 | 2168 | 0.28 | 0.45% |
|  | (545 to 1165) | (0.17 to 0.36) | (1395 to 2985) | (0.18 to 0.38) | (0.3 to 0.6) |
| GBD region |  |  |  |  |  |
| Andean Latin America | 284 | 1.25 | 681 | 1.12 | -0.47% |
|  | (193 to 376) | (0.86 to 1.65) | (467 to 918) | (0.76 to 1.51) | (-0.52 to -0.42) |
| Australasia | 181 | 0.81 | 353 | 0.75 | -0.33% |
|  | (124 to 245) | (0.55 to 1.09) | (241 to 484) | (0.5 to 1.01) | (-0.43 to -0.22) |
| Caribbean | 486 | 1.84 | 867 | 1.63 | -0.45% |
|  | (339 to 652) | (1.3 to 2.48) | (599 to 1158) | (1.12 to 2.17) | (-0.47 to -0.43) |
| Central Asia | 96 | 0.21 | 150 | 0.2 | -0.03% |
|  | (64 to 134) | (0.14 to 0.29) | (99 to 208) | (0.14 to 0.28) | (-0.07 to 0) |
| Central Europe | 2505 | 1.91 | 4394 | 2.07 | 0.37% |
|  | (1747 to 3394) | (1.34 to 2.59) | (3083 to 5913) | (1.45 to 2.77) | (0.29 to 0.44) |
| Central Latin America | 3435 | 3.65 | 8421 | 3.42 | -0.23% |
|  | (2402 to 4565) | (2.54 to 4.88) | (5901 to 11211) | (2.4 to 4.57) | (-0.26 to -0.2) |
| Central Sub-Saharan Africa | 57 | 0.19 | 137 | 0.17 | -0.38% |
|  | (37 to 80) | (0.12 to 0.25) | (88 to 189) | (0.11 to 0.23) | (-0.41 to -0.36) |
| East Asia | 7669 | 0.8 | 17364 | 0.94 | 0.80% |
|  | (5121 to 10409) | (0.55 to 1.08) | (11944 to 23231) | (0.65 to 1.25) | (0.62 to 0.99) |
| Eastern Europe | 2813 | 1.13 | 3033 | 1.01 | -0.37% |
|  | (1912 to 3812) | (0.77 to 1.52) | (2109 to 4132) | (0.7 to 1.35) | (-0.46 to -0.27) |
| Eastern Sub-Saharan Africa | 112 | 0.09 | 243 | 0.08 | -0.52% |
|  | (69 to 157) | (0.06 to 0.13) | (150 to 339) | (0.05 to 0.12) | (-0.66 to -0.39) |
| High-income Asia Pacific | 3904 | 2.12 | 8186 | 2.13 | 0.14% |
|  | (2675 to 5213) | (1.46 to 2.84) | (5757 to 10874) | (1.47 to 2.84) | (0.05 to 0.22) |
| High-income North America | 16691 | 4.72 | 29820 | 4.59 | -0.18% |
|  | (11678 to 22343) | (3.29 to 6.35) | (20881 to 39490) | (3.21 to 6.07) | (-0.27 to -0.09) |
| North Africa and Middle East | 1145 | 0.52 | 2628 | 0.49 | -0.02% |
|  | (745 to 1568) | (0.34 to 0.71) | (1720 to 3612) | (0.33 to 0.67) | (-0.18 to 0.14) |
| Oceania | 5 | 0.18 | 14 | 0.19 | 0.10% |
|  | (3 to 7) | (0.12 to 0.25) | (9 to 19) | (0.13 to 0.26) | (0.09 to 0.11) |
| South Asia | 3667 | 0.42 | 9811 | 0.54 | 1.24% |
|  | (2379 to 5112) | (0.27 to 0.58) | (6352 to 13630) | (0.35 to 0.74) | (0.99 to 1.49) |
| Southeast Asia | 560 | 0.21 | 1494 | 0.25 | 0.66% |
|  | (367 to 773) | (0.14 to 0.29) | (997 to 2018) | (0.17 to 0.34) | (0.58 to 0.74) |
| Southern Latin America | 280 | 0.64 | 701 | 0.8 | 1.02% |
|  | (193 to 385) | (0.44 to 0.87) | (484 to 957) | (0.55 to 1.09) | (0.79 to 1.25) |
| Southern Sub-Saharan Africa | 284 | 0.75 | 458 | 0.68 | -0.37% |
|  | (187 to 386) | (0.51 to 1.01) | (303 to 623) | (0.46 to 0.92) | (-0.5 to -0.24) |
| Tropical Latin America | 1707 | 1.7 | 7576 | 3.06 | 2.57% |
|  | (1154 to 2287) | (1.16 to 2.28) | (5259 to 10183) | (2.12 to 4.12) | (2.27 to 2.88) |
| Western Europe | 2827 | 0.52 | 5120 | 0.57 | 0.19% |
|  | (1920 to 3818) | (0.36 to 0.7) | (3536 to 6908) | (0.39 to 0.76) | (-0.14 to 0.51) |
| Western Sub-Saharan Africa | 197 | 0.15 | 475 | 0.15 | 0.11% |
|  | (126 to 275) | (0.1 to 0.21) | (303 to 660) | (0.1 to 0.21) | (0.04 to 0.17) |

| Table S6: Age-standardized years of life lost (YLL) for decubitus ulcers in 1990 and 2021 and their temporal trends from 1990 to 2021 at the national and territorial levels. | | | | | |
| --- | --- | --- | --- | --- | --- |
| Location | Counts 1990  (95% UI) | Age-standardized YLL rate (95% UI) | Counts 2021 (95% UI) | Age-standardized YLL rate (95% UI) | EAPC (95% CI) |
| Global | 359982 | 9.44 | 701821 | 8.45 | -0.64% |
|  | (279773 to 445955) | (7.54 to 11.43) | (515071 to 809964) | (6.23 to 9.74) | (-0.77 to -0.51) |
| SDI quintile |  |  |  |  |  |
| High SDI | 93844 | 8.64 | 93752 | 4.43 | -2.11% |
|  | (85634 to 99625) | (7.85 to 9.18) | (81758 to 101960) | (3.96 to 4.88) | (-2.31 to -1.92) |
| High-middle SDI | 30739 | 3.66 | 117323 | 6.33 | 1.85% |
|  | (27674 to 36840) | (3.28 to 4.34) | (91993 to 132003) | (4.93 to 7.11) | (1.58 to 2.12) |
| Middle SDI | 90186 | 9.22 | 210532 | 8.69 | -0.48% |
|  | (69934 to 113598) | (7.36 to 11.47) | (148646 to 245430) | (6.2 to 10.15) | (-0.56 to -0.39) |
| Low-middle SDI | 92611 | 13.46 | 185429 | 13.19 | -0.74% |
|  | (55934 to 123928) | (8.11 to 18.11) | (119050 to 233361) | (8.53 to 16.58) | (-1.08 to -0.4) |
| Low SDI | 52076 | 19.3 | 93790 | 16.17 | -0.78% |
|  | (29801 to 79775) | (11.62 to 30.73) | (56331 to 128110) | (10.27 to 21.72) | (-0.92 to -0.64) |
| GBD region |  |  |  |  |  |
| Andean Latin America | 2404 | 10.76 | 4074 | 7.07 | -2.38% |
|  | (1607 to 3015) | (7.55 to 13.26) | (3194 to 5589) | (5.53 to 9.72) | (-2.98 to -1.77) |
| Australasia | 718 | 3.25 | 748 | 1.2 | -2.95% |
|  | (641 to 782) | (2.89 to 3.54) | (618 to 854) | (1 to 1.36) | (-3.65 to -2.25) |
| Caribbean | 8921 | 34.82 | 14820 | 28.1 | -0.73% |
|  | (7528 to 10898) | (30.13 to 41.45) | (11999 to 18366) | (22.67 to 34.84) | (-0.84 to -0.63) |
| Central Asia | 95 | 0.19 | 355 | 0.46 | 2.57% |
|  | (60 to 144) | (0.12 to 0.28) | (282 to 455) | (0.37 to 0.58) | (1.94 to 3.22) |
| Central Europe | 1505 | 1.07 | 4137 | 1.94 | 1.61% |
|  | (1333 to 1745) | (0.95 to 1.24) | (3559 to 4673) | (1.67 to 2.19) | (0.73 to 2.49) |
| Central Latin America | 21245 | 24.37 | 21268 | 8.73 | -2.89% |
|  | (20286 to 22091) | (23.22 to 25.33) | (17476 to 26470) | (7.2 to 10.85) | (-3.08 to -2.7) |
| Central Sub-Saharan Africa | 6028 | 25.89 | 13834 | 24.73 | -0.39% |
|  | (1847 to 9562) | (8.45 to 39.83) | (4941 to 21944) | (10.09 to 38.78) | (-0.5 to -0.28) |
| East Asia | 11438 | 1.99 | 51389 | 2.94 | 1.16% |
|  | (9015 to 21467) | (1.53 to 3.65) | (26500 to 65496) | (1.57 to 3.72) | (0.49 to 1.84) |
| Eastern Europe | 1343 | 0.51 | 8709 | 2.78 | 4.68% |
|  | (1164 to 1519) | (0.44 to 0.57) | (8047 to 9400) | (2.58 to 2.99) | (4.22 to 5.13) |
| Eastern Sub-Saharan Africa | 36661 | 40.98 | 69163 | 34.61 | -0.59% |
|  | (20812 to 65816) | (23.8 to 77.43) | (42068 to 106359) | (22.63 to 53.16) | (-0.67 to -0.5) |
| High-income Asia Pacific | 6405 | 3.67 | 13749 | 2.33 | -3.03% |
|  | (5409 to 7115) | (3.11 to 4.08) | (11002 to 15829) | (1.88 to 2.69) | (-3.51 to -2.55) |
| High-income North America | 27722 | 7.68 | 24226 | 3.68 | -2.27% |
|  | (25449 to 29089) | (7.07 to 8.05) | (21092 to 27454) | (3.24 to 4.16) | (-2.46 to -2.07) |
| North Africa and Middle East | 21210 | 12.84 | 59544 | 14.08 | 0.26% |
|  | (15259 to 30088) | (9.27 to 18.9) | (47271 to 71057) | (11.09 to 16.71) | (0.23 to 0.3) |
| Oceania | 678 | 21.9 | 1824 | 22.62 | 0.01% |
|  | (265 to 1549) | (9.42 to 49.29) | (608 to 3770) | (8.42 to 45.03) | (-0.05 to 0.08) |
| South Asia | 83740 | 11.94 | 152197 | 10.37 | -1.78% |
|  | (50727 to 119930) | (6.86 to 17.43) | (91381 to 206354) | (6.08 to 13.93) | (-2.5 to -1.06) |
| Southeast Asia | 48733 | 18.61 | 136448 | 23.6 | 0.25% |
|  | (32073 to 71470) | (12.67 to 27.7) | (83600 to 166958) | (14.68 to 28.56) | (0.08 to 0.43) |
| Southern Latin America | 5655 | 13.06 | 24628 | 27.25 | 2.92% |
|  | (5059 to 6187) | (11.73 to 14.28) | (22014 to 26723) | (24.4 to 29.53) | (2.41 to 3.43) |
| Southern Sub-Saharan Africa | 7177 | 28.19 | 15003 | 29.87 | -0.13% |
|  | (3707 to 10094) | (15.03 to 40.18) | (9282 to 18397) | (19.46 to 36.1) | (-0.3 to 0.04) |
| Tropical Latin America | 11393 | 12.72 | 34295 | 13.78 | 0.66% |
|  | (10673 to 11959) | (11.78 to 13.41) | (30525 to 38308) | (12.25 to 15.38) | (0.53 to 0.79) |
| Western Europe | 56478 | 9.64 | 49325 | 4.21 | -2.19% |
|  | (51603 to 59509) | (8.79 to 10.16) | (41301 to 53800) | (3.61 to 4.56) | (-2.55 to -1.83) |
| Western Sub-Saharan Africa | 435 | 0.5 | 2085 | 0.73 | 0.95% |
|  | (88 to 829) | (0.1 to 0.93) | (395 to 3516) | (0.14 to 1.44) | (0.56 to 1.33) |

| Table S7: Age-standardized years lived with disability (YLD) for decubitus ulcers in 1990 and 2021 and their temporal trends from 1990 to 2021 at the national and territorial levels. | | | | | |
| --- | --- | --- | --- | --- | --- |
| Location | Counts 1990  (95% UI) | Age-standardized YLD rate (95% UI) | Counts 2021 (95% UI) | Age-standardized YLD rate (95% UI) | EAPC (95% CI) |
| Afghanistan | 32 | 0.46 | 79 | 0.44 | -0.13% |
|  | (21 to 43) | (0.31 to 0.63) | (51 to 110) | (0.29 to 0.6) | (-0.19 to -0.07) |
| Albania | 37 | 1.8 | 67 | 1.71 | -0.18% |
|  | (25 to 51) | (1.21 to 2.5) | (45 to 93) | (1.16 to 2.36) | (-0.24 to -0.13) |
| Algeria | 78 | 0.49 | 182 | 0.47 | -0.13% |
|  | (51 to 107) | (0.33 to 0.67) | (119 to 251) | (0.31 to 0.64) | (-0.17 to -0.09) |
| American Samoa | 0 | 0.31 | 0 | 0.34 | 0.33% |
|  | (0 to 0) | (0.21 to 0.43) | (0 to 0) | (0.23 to 0.47) | (0.15 to 0.51) |
| Andorra | 0 | 0.23 | 0 | 0.21 | -0.35% |
|  | (0 to 0) | (0.16 to 0.32) | (0 to 0) | (0.14 to 0.29) | (-0.41 to -0.29) |
| Angola | 10 | 0.17 | 30 | 0.16 | -0.21% |
|  | (7 to 14) | (0.11 to 0.23) | (19 to 43) | (0.1 to 0.22) | (-0.25 to -0.16) |
| Antigua and Barbuda | 2 | 2.7 | 3 | 3.18 | 0.51% |
|  | (1 to 2) | (1.86 to 3.74) | (2 to 4) | (2.2 to 4.29) | (0.46 to 0.57) |
| Argentina | 166 | 0.55 | 393 | 0.68 | 1.13% |
|  | (114 to 233) | (0.38 to 0.77) | (267 to 550) | (0.47 to 0.95) | (0.87 to 1.38) |
| Armenia | 4 | 0.16 | 7 | 0.17 | 0.19% |
|  | (3 to 6) | (0.11 to 0.23) | (5 to 10) | (0.11 to 0.23) | (0.12 to 0.26) |
| Australia | 137 | 0.73 | 256 | 0.64 | -0.48% |
|  | (93 to 187) | (0.5 to 1) | (175 to 352) | (0.43 to 0.87) | (-0.51 to -0.45) |
| Austria | 101 | 0.94 | 105 | 0.67 | -1.45% |
|  | (68 to 138) | (0.63 to 1.27) | (72 to 142) | (0.45 to 0.9) | (-1.94 to -0.96) |
| Azerbaijan | 10 | 0.2 | 18 | 0.2 | -0.01% |
|  | (7 to 14) | (0.14 to 0.28) | (12 to 25) | (0.13 to 0.28) | (-0.05 to 0.02) |
| Bahamas | 5 | 3.19 | 12 | 3.37 | 0.13% |
|  | (3 to 7) | (2.19 to 4.41) | (9 to 17) | (2.32 to 4.59) | (0.06 to 0.2) |
| Bahrain | 2 | 0.51 | 7 | 0.54 | 0.35% |
|  | (1 to 3) | (0.33 to 0.69) | (5 to 10) | (0.36 to 0.75) | (0.3 to 0.4) |
| Bangladesh | 296 | 0.37 | 578 | 0.36 | -0.11% |
|  | (191 to 412) | (0.24 to 0.51) | (375 to 808) | (0.24 to 0.5) | (-0.14 to -0.08) |
| Barbados | 13 | 4.27 | 22 | 4.4 | 0% |
|  | (9 to 17) | (2.98 to 5.68) | (14 to 30) | (2.96 to 6.01) | (-0.08 to 0.08) |
| Belarus | 138 | 1.16 | 155 | 1.11 | -0.12% |
|  | (92 to 189) | (0.77 to 1.59) | (106 to 218) | (0.77 to 1.54) | (-0.17 to -0.08) |
| Belgium | 115 | 0.76 | 128 | 0.56 | -0.77% |
|  | (77 to 157) | (0.52 to 1.04) | (87 to 177) | (0.38 to 0.76) | (-1.47 to -0.06) |
| Belize | 2 | 1.72 | 5 | 1.61 | -0.34% |
|  | (1 to 3) | (1.19 to 2.33) | (3 to 7) | (1.09 to 2.19) | (-0.41 to -0.27) |
| Benin | 5 | 0.15 | 13 | 0.15 | 0.05% |
|  | (3 to 6) | (0.1 to 0.2) | (8 to 18) | (0.1 to 0.2) | (0 to 0.11) |
| Bermuda | 1 | 1.97 | 2 | 1.83 | -0.30% |
|  | (1 to 2) | (1.39 to 2.71) | (2 to 3) | (1.23 to 2.51) | (-0.33 to -0.27) |
| Bhutan | 2 | 0.37 | 3 | 0.36 | -0.06% |
|  | (1 to 2) | (0.24 to 0.52) | (2 to 4) | (0.24 to 0.51) | (-0.1 to -0.02) |
| Bolivia (Plurinational State of) | 39 | 1.09 | 96 | 1.05 | -0.13% |
|  | (26 to 53) | (0.74 to 1.51) | (65 to 129) | (0.7 to 1.41) | (-0.17 to -0.09) |
| Bosnia and Herzegovina | 63 | 1.74 | 99 | 1.76 | 0.11% |
|  | (43 to 85) | (1.2 to 2.36) | (70 to 134) | (1.24 to 2.36) | (0.04 to 0.18) |
| Botswana | 6 | 0.74 | 14 | 0.72 | 0.34% |
|  | (4 to 9) | (0.49 to 1) | (9 to 19) | (0.49 to 0.98) | (-0.28 to 0.97) |
| Brazil | 1660 | 1.7 | 7461 | 3.09 | 2.61% |
|  | (1122 to 2223) | (1.17 to 2.28) | (5178 to 10028) | (2.15 to 4.16) | (2.31 to 2.92) |
| Brunei Darussalam | 4 | 2.82 | 9 | 2.81 | -0.03% |
|  | (3 to 5) | (1.97 to 3.83) | (6 to 12) | (1.94 to 3.88) | (-0.05 to -0.01) |
| Bulgaria | 178 | 1.81 | 224 | 1.73 | -0.16% |
|  | (116 to 250) | (1.23 to 2.5) | (152 to 315) | (1.19 to 2.38) | (-0.2 to -0.12) |
| Burkina Faso | 9 | 0.15 | 21 | 0.14 | -0.12% |
|  | (6 to 13) | (0.09 to 0.2) | (13 to 29) | (0.09 to 0.19) | (-0.17 to -0.06) |
| Burundi | 3 | 0.09 | 7 | 0.08 | -0.25% |
|  | (2 to 5) | (0.06 to 0.12) | (4 to 10) | (0.05 to 0.11) | (-0.31 to -0.19) |
| Cabo Verde | 0 | 0.16 | 1 | 0.19 | 0.37% |
|  | (0 to 1) | (0.11 to 0.23) | (1 to 1) | (0.12 to 0.25) | (0.32 to 0.41) |
| Cambodia | 9 | 0.21 | 27 | 0.25 | 0.53% |
|  | (6 to 13) | (0.14 to 0.29) | (18 to 37) | (0.17 to 0.34) | (0.51 to 0.56) |
| Cameroon | 10 | 0.15 | 31 | 0.15 | 0.15% |
|  | (6 to 14) | (0.1 to 0.21) | (20 to 44) | (0.1 to 0.21) | (0.11 to 0.19) |
| Canada | 1277 | 4.06 | 2446 | 3.59 | -0.44% |
|  | (873 to 1753) | (2.78 to 5.54) | (1674 to 3352) | (2.48 to 4.98) | (-0.5 to -0.38) |
| Central African Republic | 3 | 0.17 | 5 | 0.15 | -0.42% |
|  | (2 to 4) | (0.11 to 0.23) | (3 to 7) | (0.1 to 0.21) | (-0.48 to -0.35) |
| Chad | 6 | 0.15 | 16 | 0.16 | 0.30% |
|  | (4 to 8) | (0.1 to 0.2) | (10 to 22) | (0.1 to 0.22) | (0.25 to 0.35) |
| Chile | 82 | 0.8 | 256 | 1.04 | 1.01% |
|  | (56 to 113) | (0.54 to 1.11) | (176 to 355) | (0.71 to 1.43) | (0.77 to 1.25) |
| China | 7543 | 0.82 | 17056 | 0.96 | 0.84% |
|  | (5034 to 10239) | (0.55 to 1.1) | (11732 to 22811) | (0.66 to 1.28) | (0.65 to 1.03) |
| Colombia | 782 | 4.05 | 1797 | 3.22 | -0.73% |
|  | (532 to 1045) | (2.79 to 5.44) | (1253 to 2430) | (2.24 to 4.34) | (-0.79 to -0.68) |
| Comoros | 0 | 0.09 | 1 | 0.09 | -0.24% |
|  | (0 to 0) | (0.06 to 0.13) | (0 to 1) | (0.05 to 0.12) | (-0.29 to -0.19) |
| Congo | 3 | 0.18 | 6 | 0.16 | -0.36% |
|  | (2 to 4) | (0.12 to 0.25) | (4 to 8) | (0.11 to 0.23) | (-0.39 to -0.33) |
| Cook Islands | 0 | 0.16 | 0 | 0.17 | 0.25% |
|  | (0 to 0) | (0.11 to 0.23) | (0 to 0) | (0.12 to 0.23) | (0.21 to 0.29) |
| Costa Rica | 73 | 3.6 | 180 | 3.28 | -0.34% |
|  | (50 to 99) | (2.48 to 4.91) | (123 to 243) | (2.25 to 4.46) | (-0.38 to -0.3) |
| Croatia | 92 | 1.72 | 143 | 1.7 | 0.01% |
|  | (63 to 127) | (1.19 to 2.33) | (97 to 195) | (1.18 to 2.27) | (-0.04 to 0.05) |
| Cuba | 156 | 1.52 | 241 | 1.36 | -0.37% |
|  | (109 to 214) | (1.07 to 2.09) | (162 to 331) | (0.91 to 1.87) | (-0.44 to -0.31) |
| Cyprus | 2 | 0.31 | 6 | 0.27 | -0.32% |
|  | (1 to 3) | (0.21 to 0.42) | (4 to 8) | (0.18 to 0.37) | (-0.56 to -0.08) |
| Czechia | 270 | 2.16 | 481 | 2.34 | 0.56% |
|  | (187 to 363) | (1.51 to 2.9) | (326 to 656) | (1.58 to 3.17) | (0.38 to 0.73) |
| Democratic People's Republic of Korea | 54 | 0.37 | 104 | 0.36 | -0.09% |
|  | (36 to 74) | (0.25 to 0.51) | (71 to 143) | (0.25 to 0.5) | (-0.17 to -0.02) |
| Democratic Republic of the Congo | 40 | 0.19 | 92 | 0.17 | -0.43% |
|  | (26 to 56) | (0.13 to 0.26) | (59 to 127) | (0.11 to 0.23) | (-0.44 to -0.41) |
| Denmark | 10 | 0.13 | 13 | 0.11 | -0.63% |
|  | (7 to 14) | (0.08 to 0.18) | (9 to 18) | (0.08 to 0.16) | (-1.67 to 0.42) |
| Djibouti | 0 | 0.09 | 1 | 0.09 | -0.19% |
|  | (0 to 0) | (0.06 to 0.13) | (1 to 1) | (0.06 to 0.12) | (-0.24 to -0.14) |
| Dominica | 2 | 3.05 | 3 | 3.37 | 0.31% |
|  | (1 to 2) | (2.03 to 4.12) | (2 to 4) | (2.34 to 4.56) | (0.28 to 0.35) |
| Dominican Republic | 61 | 1.37 | 131 | 1.27 | -0.24% |
|  | (41 to 81) | (0.94 to 1.83) | (88 to 178) | (0.85 to 1.73) | (-0.32 to -0.16) |
| Ecuador | 64 | 1.03 | 216 | 1.33 | 1.15% |
|  | (43 to 88) | (0.7 to 1.42) | (148 to 294) | (0.91 to 1.81) | (0.98 to 1.32) |
| Egypt | 177 | 0.48 | 366 | 0.45 | -0.17% |
|  | (114 to 244) | (0.32 to 0.66) | (238 to 504) | (0.3 to 0.62) | (-0.23 to -0.11) |
| El Salvador | 108 | 3.05 | 200 | 2.99 | -0.01% |
|  | (74 to 146) | (2.1 to 4.14) | (137 to 274) | (2.05 to 4.07) | (-0.05 to 0.04) |
| Equatorial Guinea | 0 | 0.18 | 2 | 0.18 | 0.19% |
|  | (0 to 1) | (0.12 to 0.24) | (1 to 2) | (0.12 to 0.25) | (0.14 to 0.25) |
| Eritrea | 2 | 0.09 | 4 | 0.08 | -0.19% |
|  | (1 to 3) | (0.06 to 0.12) | (2 to 5) | (0.05 to 0.12) | (-0.25 to -0.12) |
| Estonia | 22 | 1.18 | 27 | 1.15 | -0.09% |
|  | (15 to 30) | (0.81 to 1.62) | (18 to 38) | (0.78 to 1.57) | (-0.13 to -0.05) |
| Eswatini | 4 | 0.71 | 6 | 0.7 | -0.04% |
|  | (2 to 5) | (0.47 to 0.95) | (4 to 8) | (0.47 to 0.95) | (-0.09 to 0) |
| Ethiopia | 30 | 0.09 | 63 | 0.08 | -0.42% |
|  | (19 to 43) | (0.06 to 0.13) | (39 to 88) | (0.05 to 0.11) | (-0.52 to -0.32) |
| Fiji | 1 | 0.2 | 1 | 0.22 | 0.22% |
|  | (0 to 1) | (0.13 to 0.28) | (1 to 2) | (0.15 to 0.31) | (0.19 to 0.24) |
| Finland | 39 | 0.59 | 61 | 0.57 | -0.31% |
|  | (26 to 53) | (0.4 to 0.8) | (41 to 84) | (0.38 to 0.76) | (-1.27 to 0.65) |
| France | 385 | 0.44 | 431 | 0.28 | -1.70% |
|  | (254 to 541) | (0.29 to 0.61) | (290 to 602) | (0.19 to 0.39) | (-1.82 to -1.58) |
| Gabon | 1 | 0.19 | 2 | 0.18 | -0.31% |
|  | (1 to 2) | (0.13 to 0.26) | (1 to 3) | (0.12 to 0.24) | (-0.34 to -0.29) |
| Gambia | 1 | 0.15 | 2 | 0.15 | 0.13% |
|  | (1 to 1) | (0.1 to 0.21) | (2 to 3) | (0.1 to 0.21) | (0.09 to 0.18) |
| Georgia | 15 | 0.26 | 16 | 0.27 | 0.25% |
|  | (10 to 20) | (0.17 to 0.36) | (11 to 22) | (0.18 to 0.37) | (0.18 to 0.31) |
| Germany | 232 | 0.18 | 393 | 0.19 | 1.11% |
|  | (157 to 325) | (0.12 to 0.26) | (264 to 553) | (0.13 to 0.26) | (0.43 to 1.79) |
| Ghana | 15 | 0.15 | 35 | 0.14 | -0.28% |
|  | (9 to 21) | (0.1 to 0.2) | (22 to 50) | (0.09 to 0.19) | (-0.33 to -0.23) |
| Greece | 13 | 0.09 | 28 | 0.11 | 1.22% |
|  | (9 to 18) | (0.06 to 0.13) | (18 to 38) | (0.07 to 0.15) | (0.95 to 1.48) |
| Greenland | 1 | 3.99 | 2 | 3.72 | -0.19% |
|  | (1 to 2) | (2.74 to 5.5) | (2 to 3) | (2.64 to 5.04) | (-0.25 to -0.12) |
| Grenada | 2 | 2.76 | 3 | 3.16 | 0.45% |
|  | (1 to 3) | (1.87 to 3.9) | (2 to 4) | (2.16 to 4.26) | (0.36 to 0.53) |
| Guam | 0 | 0.19 | 0 | 0.2 | 0.17% |
|  | (0 to 0) | (0.13 to 0.26) | (0 to 1) | (0.14 to 0.27) | (0.11 to 0.24) |
| Guatemala | 150 | 3.98 | 348 | 2.96 | -1.25% |
|  | (102 to 202) | (2.76 to 5.41) | (235 to 471) | (2.02 to 4.02) | (-1.38 to -1.13) |
| Guinea | 6 | 0.15 | 13 | 0.15 | 0.16% |
|  | (4 to 9) | (0.1 to 0.21) | (8 to 18) | (0.1 to 0.21) | (0.13 to 0.2) |
| Guinea-Bissau | 1 | 0.15 | 2 | 0.15 | 0.10% |
|  | (1 to 1) | (0.09 to 0.2) | (1 to 3) | (0.1 to 0.2) | (0.06 to 0.15) |
| Guyana | 7 | 1.49 | 9 | 1.42 | -0.24% |
|  | (4 to 9) | (1.03 to 2.03) | (6 to 12) | (0.97 to 1.92) | (-0.31 to -0.16) |
| Haiti | 69 | 2.25 | 144 | 2.09 | -0.23% |
|  | (45 to 94) | (1.48 to 3.08) | (98 to 197) | (1.42 to 2.9) | (-0.27 to -0.19) |
| Honduras | 83 | 3.47 | 225 | 3.45 | -0.03% |
|  | (56 to 113) | (2.42 to 4.78) | (149 to 304) | (2.34 to 4.66) | (-0.07 to 0.01) |
| Hungary | 232 | 1.8 | 333 | 1.77 | -0.03% |
|  | (159 to 325) | (1.24 to 2.49) | (228 to 455) | (1.2 to 2.42) | (-0.09 to 0.04) |
| Iceland | 0 | 0.08 | 0 | 0.07 | 0.21% |
|  | (0 to 0) | (0.06 to 0.12) | (0 to 1) | (0.05 to 0.11) | (-0.25 to 0.66) |
| India | 3010 | 0.43 | 8419 | 0.59 | 1.50% |
|  | (1957 to 4200) | (0.28 to 0.6) | (5450 to 11646) | (0.38 to 0.81) | (1.21 to 1.79) |
| Indonesia | 166 | 0.14 | 304 | 0.13 | -0.42% |
|  | (105 to 234) | (0.09 to 0.2) | (195 to 423) | (0.08 to 0.18) | (-0.53 to -0.31) |
| Iran (Islamic Republic of) | 156 | 0.44 | 351 | 0.41 | -0.09% |
|  | (101 to 214) | (0.29 to 0.59) | (229 to 485) | (0.28 to 0.56) | (-0.42 to 0.23) |
| Iraq | 56 | 0.48 | 145 | 0.45 | -0.20% |
|  | (36 to 77) | (0.31 to 0.65) | (95 to 198) | (0.3 to 0.61) | (-0.27 to -0.14) |
| Ireland | 14 | 0.35 | 15 | 0.2 | -2.22% |
|  | (10 to 20) | (0.24 to 0.48) | (10 to 21) | (0.14 to 0.28) | (-2.39 to -2.04) |
| Israel | 27 | 0.55 | 90 | 0.69 | 0.65% |
|  | (18 to 38) | (0.37 to 0.78) | (60 to 125) | (0.46 to 0.95) | (0.53 to 0.78) |
| Italy | 726 | 0.94 | 1712 | 1.19 | 1.01% |
|  | (493 to 988) | (0.64 to 1.27) | (1203 to 2292) | (0.83 to 1.59) | (0.51 to 1.52) |
| Jamaica | 49 | 2.58 | 89 | 2.7 | 0.09% |
|  | (34 to 67) | (1.78 to 3.5) | (62 to 120) | (1.87 to 3.67) | (0.02 to 0.16) |
| Japan | 3084 | 2.1 | 6328 | 2.17 | 0.25% |
|  | (2111 to 4123) | (1.45 to 2.82) | (4487 to 8487) | (1.5 to 2.89) | (0.15 to 0.36) |
| Jordan | 5 | 0.32 | 27 | 0.37 | 0.55% |
|  | (3 to 7) | (0.22 to 0.44) | (18 to 37) | (0.24 to 0.5) | (0.46 to 0.65) |
| Kazakhstan | 24 | 0.2 | 31 | 0.21 | 0.09% |
|  | (16 to 34) | (0.14 to 0.29) | (21 to 42) | (0.14 to 0.29) | (0.06 to 0.12) |
| Kenya | 14 | 0.09 | 31 | 0.08 | -1.84% |
|  | (9 to 20) | (0.06 to 0.13) | (19 to 44) | (0.05 to 0.12) | (-2.43 to -1.24) |
| Kiribati | 0 | 0.3 | 0 | 0.35 | 0.51% |
|  | (0 to 0) | (0.2 to 0.41) | (0 to 0) | (0.24 to 0.47) | (0.43 to 0.59) |
| Kuwait | 7 | 0.52 | 22 | 0.47 | -0.29% |
|  | (4 to 9) | (0.34 to 0.7) | (14 to 31) | (0.31 to 0.65) | (-0.38 to -0.21) |
| Kyrgyzstan | 6 | 0.2 | 9 | 0.2 | -0.08% |
|  | (4 to 8) | (0.14 to 0.28) | (6 to 12) | (0.13 to 0.27) | (-0.12 to -0.04) |
| Lao People's Democratic Republic | 4 | 0.22 | 10 | 0.24 | 0.16% |
|  | (3 to 6) | (0.15 to 0.31) | (7 to 14) | (0.16 to 0.33) | (0.14 to 0.19) |
| Latvia | 45 | 1.39 | 49 | 1.38 | 0.24% |
|  | (30 to 63) | (0.93 to 1.92) | (33 to 69) | (0.94 to 1.91) | (-0.16 to 0.65) |
| Lebanon | 11 | 0.48 | 28 | 0.45 | -0.19% |
|  | (7 to 16) | (0.32 to 0.66) | (18 to 38) | (0.3 to 0.61) | (-0.23 to -0.16) |
| Lesotho | 8 | 0.68 | 10 | 0.68 | 0.01% |
|  | (5 to 10) | (0.46 to 0.92) | (6 to 13) | (0.45 to 0.91) | (-0.05 to 0.08) |
| Liberia | 2 | 0.15 | 5 | 0.15 | 0.06% |
|  | (2 to 3) | (0.1 to 0.21) | (3 to 8) | (0.1 to 0.21) | (0.01 to 0.12) |
| Libya | 14 | 0.5 | 30 | 0.46 | -0.28% |
|  | (9 to 19) | (0.33 to 0.69) | (20 to 41) | (0.3 to 0.62) | (-0.33 to -0.24) |
| Lithuania | 40 | 0.96 | 46 | 0.91 | -0.17% |
|  | (27 to 56) | (0.65 to 1.31) | (31 to 66) | (0.61 to 1.25) | (-0.42 to 0.07) |
| Luxembourg | 1 | 0.25 | 2 | 0.2 | -0.60% |
|  | (1 to 2) | (0.17 to 0.34) | (1 to 3) | (0.14 to 0.28) | (-1.18 to -0.02) |
| Madagascar | 7 | 0.09 | 16 | 0.08 | -0.24% |
|  | (4 to 10) | (0.06 to 0.13) | (10 to 23) | (0.05 to 0.12) | (-0.3 to -0.19) |
| Malawi | 6 | 0.09 | 11 | 0.08 | -0.22% |
|  | (3 to 8) | (0.06 to 0.12) | (7 to 15) | (0.05 to 0.12) | (-0.29 to -0.16) |
| Malaysia | 32 | 0.34 | 147 | 0.55 | 2.09% |
|  | (21 to 44) | (0.23 to 0.47) | (101 to 200) | (0.38 to 0.76) | (1.92 to 2.28) |
| Maldives | 0 | 0.3 | 1 | 0.31 | 0.09% |
|  | (0 to 0) | (0.2 to 0.41) | (1 to 1) | (0.21 to 0.42) | (-0.02 to 0.2) |
| Mali | 8 | 0.15 | 22 | 0.15 | 0.16% |
|  | (5 to 12) | (0.1 to 0.21) | (14 to 30) | (0.1 to 0.21) | (0.11 to 0.21) |
| Malta | 2 | 0.59 | 6 | 0.6 | 0.02% |
|  | (2 to 3) | (0.39 to 0.81) | (4 to 9) | (0.4 to 0.84) | (-0.23 to 0.26) |
| Marshall Islands | 0 | 0.2 | 0 | 0.21 | 0.12% |
|  | (0 to 0) | (0.14 to 0.28) | (0 to 0) | (0.14 to 0.29) | (0.09 to 0.16) |
| Mauritania | 2 | 0.15 | 5 | 0.15 | 0.01% |
|  | (1 to 3) | (0.1 to 0.21) | (3 to 6) | (0.1 to 0.2) | (-0.04 to 0.05) |
| Mauritius | 1 | 0.19 | 5 | 0.28 | 1.48% |
|  | (1 to 2) | (0.13 to 0.26) | (3 to 6) | (0.19 to 0.39) | (1.41 to 1.55) |
| Mexico | 1741 | 3.7 | 4492 | 3.72 | -0.05% |
|  | (1204 to 2337) | (2.55 to 4.95) | (3128 to 5963) | (2.58 to 4.94) | (-0.11 to 0.01) |
| Micronesia (Federated States of) | 0 | 0.2 | 0 | 0.22 | 0.33% |
|  | (0 to 0) | (0.13 to 0.27) | (0 to 0) | (0.15 to 0.3) | (0.3 to 0.36) |
| Monaco | 0 | 0.23 | 0 | 0.21 | -0.28% |
|  | (0 to 0) | (0.16 to 0.32) | (0 to 0) | (0.14 to 0.29) | (-0.34 to -0.22) |
| Mongolia | 2 | 0.21 | 4 | 0.2 | -0.04% |
|  | (1 to 3) | (0.14 to 0.29) | (3 to 6) | (0.14 to 0.28) | (-0.07 to -0.01) |
| Montenegro | 11 | 1.85 | 15 | 1.73 | -0.22% |
|  | (7 to 15) | (1.23 to 2.54) | (10 to 21) | (1.18 to 2.4) | (-0.26 to -0.17) |
| Morocco | 86 | 0.48 | 161 | 0.45 | -0.14% |
|  | (57 to 118) | (0.32 to 0.65) | (105 to 222) | (0.3 to 0.62) | (-0.18 to -0.09) |
| Mozambique | 8 | 0.09 | 16 | 0.08 | -0.16% |
|  | (5 to 11) | (0.06 to 0.12) | (10 to 23) | (0.05 to 0.12) | (-0.23 to -0.09) |
| Myanmar | 50 | 0.23 | 111 | 0.26 | 0.33% |
|  | (33 to 68) | (0.16 to 0.32) | (75 to 152) | (0.17 to 0.35) | (0.32 to 0.35) |
| Namibia | 7 | 0.76 | 13 | 0.72 | -0.15% |
|  | (5 to 10) | (0.51 to 1.02) | (9 to 18) | (0.49 to 0.97) | (-0.18 to -0.12) |
| Nauru | 0 | 0.2 | 0 | 0.22 | 0.31% |
|  | (0 to 0) | (0.13 to 0.27) | (0 to 0) | (0.15 to 0.3) | (0.29 to 0.33) |
| Nepal | 51 | 0.35 | 114 | 0.39 | 0.92% |
|  | (33 to 72) | (0.23 to 0.49) | (74 to 156) | (0.26 to 0.53) | (0.58 to 1.26) |
| Netherlands | 87 | 0.43 | 97 | 0.28 | -1.68% |
|  | (58 to 122) | (0.29 to 0.59) | (65 to 136) | (0.19 to 0.39) | (-1.78 to -1.58) |
| New Zealand | 44 | 1.19 | 97 | 1.32 | 0.13% |
|  | (30 to 61) | (0.81 to 1.62) | (64 to 132) | (0.89 to 1.77) | (-0.18 to 0.44) |
| Nicaragua | 64 | 3.07 | 160 | 3.06 | -0.01% |
|  | (43 to 86) | (2.1 to 4.2) | (108 to 217) | (2.09 to 4.14) | (-0.06 to 0.04) |
| Niger | 7 | 0.15 | 21 | 0.15 | 0.14% |
|  | (5 to 10) | (0.1 to 0.21) | (14 to 30) | (0.1 to 0.21) | (0.08 to 0.2) |
| Nigeria | 97 | 0.15 | 225 | 0.15 | 0.14% |
|  | (62 to 136) | (0.1 to 0.21) | (142 to 311) | (0.1 to 0.21) | (0.06 to 0.22) |
| Niue | 0 | 0.21 | 0 | 0.2 | -0.01% |
|  | (0 to 0) | (0.14 to 0.28) | (0 to 0) | (0.14 to 0.28) | (-0.03 to 0.01) |
| North Macedonia | 31 | 1.81 | 47 | 1.73 | -0.13% |
|  | (21 to 43) | (1.23 to 2.48) | (32 to 65) | (1.19 to 2.35) | (-0.18 to -0.08) |
| Northern Mariana Islands | 0 | 0.25 | 0 | 0.23 | -0.42% |
|  | (0 to 0) | (0.17 to 0.35) | (0 to 0) | (0.16 to 0.32) | (-0.52 to -0.31) |
| Norway | 81 | 1.45 | 110 | 1.42 | 1.04% |
|  | (55 to 111) | (1 to 1.96) | (75 to 149) | (0.97 to 1.92) | (0.23 to 1.86) |
| Oman | 7 | 0.53 | 21 | 0.54 | 0.14% |
|  | (4 to 10) | (0.35 to 0.73) | (13 to 29) | (0.36 to 0.75) | (0.09 to 0.18) |
| Pakistan | 308 | 0.37 | 696 | 0.34 | -0.27% |
|  | (201 to 428) | (0.24 to 0.52) | (456 to 973) | (0.22 to 0.48) | (-0.34 to -0.19) |
| Palau | 0 | 0.16 | 0 | 0.14 | -0.41% |
|  | (0 to 0) | (0.1 to 0.21) | (0 to 0) | (0.09 to 0.19) | (-0.5 to -0.31) |
| Palestine | 6 | 0.45 | 16 | 0.43 | -0.12% |
|  | (4 to 8) | (0.3 to 0.62) | (11 to 23) | (0.29 to 0.59) | (-0.17 to -0.06) |
| Panama | 61 | 3.69 | 173 | 3.82 | 0.16% |
|  | (42 to 82) | (2.53 to 4.96) | (121 to 236) | (2.65 to 5.21) | (0.14 to 0.19) |
| Papua New Guinea | 3 | 0.17 | 9 | 0.18 | 0.11% |
|  | (2 to 4) | (0.11 to 0.24) | (6 to 13) | (0.12 to 0.24) | (0.07 to 0.14) |
| Paraguay | 47 | 1.76 | 116 | 1.87 | 0.25% |
|  | (32 to 63) | (1.19 to 2.41) | (79 to 156) | (1.29 to 2.54) | (0.22 to 0.28) |
| Peru | 180 | 1.37 | 368 | 1.04 | -1.22% |
|  | (123 to 240) | (0.95 to 1.85) | (251 to 498) | (0.71 to 1.41) | (-1.32 to -1.12) |
| Philippines | 127 | 0.42 | 369 | 0.47 | 0.51% |
|  | (85 to 171) | (0.29 to 0.57) | (248 to 502) | (0.32 to 0.64) | (0.35 to 0.67) |
| Poland | 847 | 2.13 | 1816 | 2.58 | 0.77% |
|  | (596 to 1160) | (1.5 to 2.9) | (1292 to 2428) | (1.84 to 3.44) | (0.71 to 0.83) |
| Portugal | 18 | 0.14 | 23 | 0.09 | -2.41% |
|  | (12 to 25) | (0.09 to 0.2) | (16 to 33) | (0.06 to 0.13) | (-2.94 to -1.88) |
| Puerto Rico | 73 | 2.08 | 112 | 1.55 | -1.12% |
|  | (50 to 99) | (1.45 to 2.82) | (75 to 152) | (1.05 to 2.11) | (-1.17 to -1.07) |
| Qatar | 5 | 1.39 | 41 | 1.34 | -0.38% |
|  | (3 to 7) | (0.94 to 1.89) | (27 to 57) | (0.9 to 1.82) | (-0.66 to -0.1) |
| Republic of Korea | 753 | 2.35 | 1685 | 2.21 | -0.28% |
|  | (501 to 1014) | (1.61 to 3.15) | (1150 to 2333) | (1.49 to 3.03) | (-0.38 to -0.18) |
| Republic of Moldova | 48 | 1.17 | 60 | 1.14 | -0.09% |
|  | (32 to 65) | (0.78 to 1.59) | (41 to 83) | (0.76 to 1.55) | (-0.12 to -0.05) |
| Romania | 437 | 1.87 | 621 | 1.73 | -0.12% |
|  | (302 to 596) | (1.3 to 2.52) | (434 to 848) | (1.2 to 2.36) | (-0.28 to 0.04) |
| Russian Federation | 1824 | 1.14 | 2039 | 1 | -0.43% |
|  | (1235 to 2464) | (0.78 to 1.52) | (1394 to 2767) | (0.69 to 1.33) | (-0.51 to -0.34) |
| Rwanda | 4 | 0.09 | 8 | 0.08 | -0.21% |
|  | (3 to 6) | (0.06 to 0.12) | (5 to 11) | (0.05 to 0.12) | (-0.26 to -0.17) |
| Saint Kitts and Nevis | 1 | 2.99 | 2 | 3.04 | -0.02% |
|  | (1 to 2) | (2.04 to 4.13) | (1 to 2) | (2.11 to 4.13) | (-0.11 to 0.06) |
| Saint Lucia | 2 | 2.61 | 6 | 2.58 | -0.19% |
|  | (2 to 3) | (1.78 to 3.6) | (4 to 8) | (1.81 to 3.48) | (-0.27 to -0.1) |
| Saint Vincent and the Grenadines | 2 | 2.17 | 4 | 2.8 | 0.68% |
|  | (1 to 2) | (1.46 to 2.97) | (3 to 5) | (1.93 to 3.78) | (0.6 to 0.77) |
| Samoa | 0 | 0.21 | 0 | 0.21 | 0.14% |
|  | (0 to 0) | (0.14 to 0.29) | (0 to 0) | (0.14 to 0.29) | (0.11 to 0.16) |
| San Marino | 0 | 0.23 | 0 | 0.21 | -0.24% |
|  | (0 to 0) | (0.15 to 0.31) | (0 to 0) | (0.14 to 0.28) | (-0.34 to -0.15) |
| Sao Tome and Principe | 0 | 0.15 | 0 | 0.16 | 0.26% |
|  | (0 to 0) | (0.09 to 0.2) | (0 to 0) | (0.1 to 0.21) | (0.22 to 0.3) |
| Saudi Arabia | 72 | 0.97 | 231 | 1.09 | 0.35% |
|  | (48 to 98) | (0.65 to 1.3) | (155 to 313) | (0.75 to 1.47) | (0.23 to 0.47) |
| Senegal | 7 | 0.15 | 17 | 0.16 | 0.21% |
|  | (5 to 10) | (0.1 to 0.21) | (11 to 24) | (0.1 to 0.22) | (0.16 to 0.25) |
| Serbia | 110 | 1.21 | 211 | 1.33 | 0.34% |
|  | (75 to 150) | (0.83 to 1.66) | (144 to 299) | (0.91 to 1.88) | (0.3 to 0.38) |
| Seychelles | 0 | 0.21 | 0 | 0.25 | 0.70% |
|  | (0 to 0) | (0.14 to 0.28) | (0 to 0) | (0.17 to 0.33) | (0.64 to 0.76) |
| Sierra Leone | 4 | 0.15 | 9 | 0.15 | 0.09% |
|  | (3 to 6) | (0.1 to 0.2) | (6 to 13) | (0.1 to 0.21) | (0.04 to 0.14) |
| Singapore | 63 | 2.66 | 164 | 2.2 | -0.42% |
|  | (42 to 86) | (1.83 to 3.66) | (114 to 223) | (1.52 to 2.99) | (-0.54 to -0.29) |
| Slovakia | 114 | 2.05 | 188 | 2.18 | 0.13% |
|  | (80 to 154) | (1.43 to 2.77) | (130 to 260) | (1.51 to 3.01) | (-0.04 to 0.3) |
| Slovenia | 42 | 1.82 | 85 | 1.85 | 0.08% |
|  | (28 to 57) | (1.25 to 2.48) | (59 to 119) | (1.26 to 2.53) | (-0.14 to 0.3) |
| Solomon Islands | 0 | 0.17 | 1 | 0.19 | 0.32% |
|  | (0 to 0) | (0.12 to 0.24) | (0 to 1) | (0.13 to 0.26) | (0.31 to 0.33) |
| Somalia | 4 | 0.09 | 11 | 0.08 | -0.25% |
|  | (3 to 6) | (0.06 to 0.12) | (6 to 15) | (0.05 to 0.11) | (-0.31 to -0.19) |
| South Africa | 211 | 0.76 | 344 | 0.68 | -0.46% |
|  | (140 to 288) | (0.52 to 1.03) | (229 to 468) | (0.46 to 0.92) | (-0.59 to -0.33) |
| South Sudan | 4 | 0.09 | 5 | 0.08 | -0.31% |
|  | (2 to 5) | (0.06 to 0.13) | (3 to 8) | (0.05 to 0.12) | (-0.37 to -0.25) |
| Spain | 108 | 0.2 | 258 | 0.25 | 0.80% |
|  | (70 to 152) | (0.13 to 0.28) | (174 to 355) | (0.17 to 0.34) | (0.6 to 1) |
| Sri Lanka | 17 | 0.15 | 35 | 0.14 | -0.10% |
|  | (11 to 24) | (0.1 to 0.2) | (23 to 49) | (0.1 to 0.19) | (-0.18 to -0.02) |
| Sudan | 59 | 0.47 | 134 | 0.44 | -0.20% |
|  | (39 to 81) | (0.31 to 0.64) | (87 to 185) | (0.29 to 0.61) | (-0.26 to -0.13) |
| Suriname | 7 | 2.83 | 18 | 3.08 | 0.27% |
|  | (5 to 10) | (1.94 to 3.89) | (13 to 25) | (2.11 to 4.16) | (0.23 to 0.3) |
| Sweden | 68 | 0.49 | 88 | 0.5 | 0.01% |
|  | (46 to 92) | (0.33 to 0.67) | (60 to 121) | (0.33 to 0.68) | (-0.58 to 0.6) |
| Switzerland | 41 | 0.41 | 63 | 0.37 | -0.38% |
|  | (28 to 57) | (0.27 to 0.56) | (43 to 86) | (0.25 to 0.51) | (-1.15 to 0.4) |
| Syrian Arab Republic | 37 | 0.48 | 59 | 0.44 | -0.25% |
|  | (24 to 51) | (0.32 to 0.66) | (39 to 81) | (0.29 to 0.59) | (-0.35 to -0.15) |
| Taiwan (Province of China) | 72 | 0.55 | 204 | 0.49 | -0.72% |
|  | (48 to 98) | (0.38 to 0.75) | (138 to 280) | (0.33 to 0.68) | (-0.99 to -0.45) |
| Tajikistan | 6 | 0.2 | 11 | 0.19 | -0.14% |
|  | (4 to 8) | (0.14 to 0.28) | (7 to 15) | (0.13 to 0.27) | (-0.18 to -0.1) |
| Thailand | 80 | 0.23 | 336 | 0.33 | 1.40% |
|  | (54 to 110) | (0.16 to 0.31) | (225 to 462) | (0.22 to 0.45) | (1.26 to 1.53) |
| Timor-Leste | 1 | 0.2 | 2 | 0.22 | 0.40% |
|  | (0 to 1) | (0.13 to 0.28) | (1 to 2) | (0.15 to 0.3) | (0.37 to 0.43) |
| Togo | 3 | 0.15 | 9 | 0.15 | 0.04% |
|  | (2 to 5) | (0.1 to 0.21) | (5 to 12) | (0.09 to 0.2) | (-0.01 to 0.09) |
| Tokelau | 0 | 0.19 | 0 | 0.2 | 0.15% |
|  | (0 to 0) | (0.13 to 0.26) | (0 to 0) | (0.13 to 0.27) | (0.14 to 0.16) |
| Tonga | 0 | 0.19 | 0 | 0.2 | 0.26% |
|  | (0 to 0) | (0.12 to 0.26) | (0 to 0) | (0.14 to 0.28) | (0.22 to 0.31) |
| Trinidad and Tobago | 16 | 1.86 | 28 | 1.62 | -0.51% |
|  | (11 to 21) | (1.27 to 2.55) | (20 to 39) | (1.12 to 2.21) | (-0.55 to -0.47) |
| Tunisia | 29 | 0.48 | 57 | 0.45 | -0.19% |
|  | (19 to 40) | (0.32 to 0.65) | (38 to 78) | (0.3 to 0.61) | (-0.24 to -0.14) |
| Turkmenistan | 4 | 0.2 | 8 | 0.2 | -0.04% |
|  | (3 to 6) | (0.14 to 0.28) | (5 to 11) | (0.13 to 0.28) | (-0.07 to -0.01) |
| Tuvalu | 0 | 0.2 | 0 | 0.2 | 0.05% |
|  | (0 to 0) | (0.13 to 0.28) | (0 to 0) | (0.14 to 0.28) | (0.04 to 0.07) |
| Uganda | 10 | 0.09 | 23 | 0.08 | -0.18% |
|  | (6 to 13) | (0.06 to 0.12) | (14 to 33) | (0.05 to 0.12) | (-0.24 to -0.13) |
| Ukraine | 696 | 1.11 | 657 | 1 | -0.35% |
|  | (471 to 949) | (0.76 to 1.51) | (450 to 918) | (0.69 to 1.36) | (-0.44 to -0.25) |
| United Arab Emirates | 8 | 0.56 | 55 | 0.53 | -0.13% |
|  | (5 to 11) | (0.37 to 0.76) | (36 to 78) | (0.35 to 0.73) | (-0.21 to -0.06) |
| United Kingdom | 751 | 0.92 | 1485 | 1.26 | 0.12% |
|  | (513 to 1004) | (0.63 to 1.24) | (1019 to 2007) | (0.86 to 1.7) | (-0.16 to 0.41) |
| United Republic of Tanzania | 15 | 0.09 | 34 | 0.09 | -0.19% |
|  | (9 to 21) | (0.06 to 0.13) | (21 to 48) | (0.05 to 0.12) | (-0.24 to -0.14) |
| United States of America | 15412 | 4.78 | 27372 | 4.73 | -0.13% |
|  | (10790 to 20523) | (3.33 to 6.41) | (19165 to 36180) | (3.3 to 6.23) | (-0.24 to -0.03) |
| United States Virgin Islands | 2 | 2.31 | 5 | 2.66 | 0.45% |
|  | (1 to 2) | (1.56 to 3.14) | (3 to 6) | (1.78 to 3.63) | (0.39 to 0.51) |
| Uruguay | 32 | 0.83 | 51 | 0.85 | 0.05% |
|  | (22 to 44) | (0.57 to 1.13) | (35 to 71) | (0.58 to 1.16) | (-0.05 to 0.14) |
| Uzbekistan | 25 | 0.2 | 47 | 0.2 | -0.04% |
|  | (17 to 35) | (0.14 to 0.28) | (31 to 65) | (0.13 to 0.28) | (-0.08 to 0) |
| Vanuatu | 0 | 0.17 | 0 | 0.19 | 0.39% |
|  | (0 to 0) | (0.11 to 0.23) | (0 to 0) | (0.13 to 0.26) | (0.36 to 0.41) |
| Venezuela (Bolivarian Republic of) | 373 | 3.08 | 847 | 2.93 | -0.07% |
|  | (254 to 511) | (2.14 to 4.26) | (566 to 1142) | (1.96 to 3.94) | (-0.15 to 0.01) |
| Viet Nam | 71 | 0.17 | 144 | 0.15 | -0.26% |
|  | (47 to 99) | (0.11 to 0.23) | (95 to 199) | (0.1 to 0.21) | (-0.31 to -0.22) |
| Yemen | 35 | 0.47 | 98 | 0.43 | -0.28% |
|  | (23 to 48) | (0.31 to 0.64) | (64 to 135) | (0.29 to 0.59) | (-0.34 to -0.21) |
| Zambia | 4 | 0.09 | 11 | 0.08 | -0.19% |
|  | (3 to 6) | (0.06 to 0.12) | (7 to 15) | (0.05 to 0.12) | (-0.24 to -0.14) |
| Zimbabwe | 48 | 0.7 | 72 | 0.66 | -0.21% |
|  | (31 to 65) | (0.48 to 0.95) | (47 to 98) | (0.44 to 0.9) | (-0.27 to -0.16) |

| Table S8: Age-standardized years of life lost (YLL) for decubitus ulcers in 1990 and 2021 and their temporal trends from 1990 to 2021 at the national and territorial levels. | | | | | |
| --- | --- | --- | --- | --- | --- |
| Location | Counts 1990  (95% UI) | Age-standardized YLL rate (95% UI) | Counts 2021 (95% UI) | Age-standardized YLL rate (95% UI) | EAPC (95% CI) |
| Afghanistan | 1418 | 21.54 | 2638 | 25.7 | 0.44% |
|  | (896 to 2149) | (13.27 to 34.02) | (1562 to 3708) | (15.96 to 36.28) | (0.32 to 0.55) |
| Albania | 11 | 0.53 | 33 | 0.81 | 0.11% |
|  | (5 to 19) | (0.25 to 0.88) | (12 to 57) | (0.31 to 1.39) | (-0.51 to 0.73) |
| Algeria | 1133 | 10.14 | 4079 | 14.26 | 0.78% |
|  | (772 to 1638) | (7.24 to 14.42) | (2806 to 5417) | (10.18 to 18.71) | (0.48 to 1.08) |
| American Samoa | 19 | 77.18 | 44 | 93.15 | 0.76% |
|  | (8 to 32) | (36.17 to 125.76) | (22 to 77) | (49.51 to 161.61) | (0.42 to 1.11) |
| Andorra | 1 | 1.47 | 2 | 0.96 | -1.31% |
|  | (0 to 1) | (0.97 to 2.11) | (1 to 2) | (0.62 to 1.35) | (-1.44 to -1.18) |
| Angola | 1089 | 25.18 | 2590 | 21.25 | -2.02% |
|  | (389 to 1891) | (9.99 to 40.62) | (870 to 4823) | (8.36 to 38.08) | (-2.7 to -1.34) |
| Antigua and Barbuda | 39 | 68.15 | 101 | 105.61 | 2.17% |
|  | (34 to 43) | (60.46 to 76.74) | (92 to 109) | (96.11 to 113.65) | (1.84 to 2.51) |
| Argentina | 4105 | 13.49 | 18131 | 31.38 | 3.33% |
|  | (3636 to 4538) | (11.95 to 14.88) | (16211 to 19740) | (28.14 to 34.19) | (2.81 to 3.85) |
| Armenia | 14 | 0.49 | 62 | 1.42 | 3.24% |
|  | (7 to 19) | (0.27 to 0.68) | (46 to 81) | (1.05 to 1.87) | (2.46 to 4.03) |
| Australia | 594 | 3.25 | 648 | 1.22 | -2.73% |
|  | (528 to 652) | (2.89 to 3.56) | (535 to 741) | (1.03 to 1.39) | (-3.48 to -1.97) |
| Austria | 257 | 2.06 | 226 | 0.98 | -2.77% |
|  | (232 to 277) | (1.85 to 2.23) | (180 to 266) | (0.79 to 1.15) | (-3.11 to -2.43) |
| Azerbaijan | 25 | 0.43 | 47 | 0.41 | 0.03% |
|  | (4 to 54) | (0.07 to 0.89) | (5 to 112) | (0.05 to 0.98) | (-0.7 to 0.77) |
| Bahamas | 198 | 121.84 | 462 | 123.13 | 0.61% |
|  | (182 to 216) | (112.11 to 133.12) | (377 to 564) | (101.19 to 149.4) | (0.32 to 0.89) |
| Bahrain | 71 | 75.43 | 265 | 65.34 | -1.89% |
|  | (30 to 134) | (36.18 to 133.78) | (103 to 472) | (29.94 to 106.36) | (-2.32 to -1.46) |
| Bangladesh | 2172 | 6.13 | 6686 | 8.24 | 1.11% |
|  | (1091 to 3682) | (2.78 to 11.38) | (3542 to 10719) | (3.91 to 13.92) | (0.86 to 1.35) |
| Barbados | 794 | 265.48 | 958 | 191.96 | -0.52% |
|  | (714 to 868) | (239.92 to 288.66) | (776 to 1148) | (155.94 to 230.12) | (-0.74 to -0.3) |
| Belarus | 224 | 1.83 | 375 | 2.6 | 2.20% |
|  | (163 to 286) | (1.34 to 2.33) | (303 to 456) | (2.09 to 3.17) | (1.69 to 2.71) |
| Belgium | 4760 | 30.17 | 2872 | 9.98 | -2.52% |
|  | (4216 to 5166) | (26.7 to 32.69) | (2321 to 3269) | (8.27 to 11.26) | (-3.03 to -2) |
| Belize | 19 | 18.75 | 47 | 16.88 | 0.24% |
|  | (14 to 23) | (14 to 22.66) | (42 to 53) | (14.79 to 19.05) | (-0.18 to 0.66) |
| Benin | 4 | 0.17 | 35 | 0.39 | 2.31% |
|  | (1 to 7) | (0.03 to 0.34) | (3 to 84) | (0.04 to 0.87) | (1.64 to 2.97) |
| Bermuda | 13 | 22.95 | 31 | 21.21 | 0.07% |
|  | (11 to 15) | (19.62 to 26.3) | (25 to 37) | (17.77 to 25.76) | (-0.24 to 0.39) |
| Bhutan | 34 | 9.42 | 50 | 7.82 | -0.49% |
|  | (16 to 55) | (4.63 to 15.31) | (25 to 85) | (3.78 to 13.4) | (-0.56 to -0.43) |
| Bolivia (Plurinational State of) | 351 | 10.26 | 917 | 11.04 | 0.16% |
|  | (220 to 500) | (6.86 to 14.17) | (554 to 1371) | (6.94 to 16.04) | (0.12 to 0.21) |
| Bosnia and Herzegovina | 126 | 3.01 | 149 | 2.59 | -0.98% |
|  | (78 to 181) | (1.9 to 4.33) | (81 to 219) | (1.39 to 3.79) | (-1.15 to -0.82) |
| Botswana | 120 | 24.39 | 343 | 27.57 | 1% |
|  | (71 to 186) | (15.08 to 38.86) | (228 to 505) | (19.02 to 40.05) | (0.71 to 1.3) |
| Brazil | 11232 | 12.88 | 33787 | 13.89 | 0.67% |
|  | (10520 to 11760) | (11.98 to 13.53) | (30094 to 37684) | (12.37 to 15.49) | (0.54 to 0.8) |
| Brunei Darussalam | 20 | 22.53 | 45 | 19.27 | -0.51% |
|  | (15 to 27) | (16.66 to 29.27) | (29 to 62) | (13.5 to 26.12) | (-0.77 to -0.25) |
| Bulgaria | 11 | 0.11 | 61 | 0.46 | 5.22% |
|  | (9 to 13) | (0.09 to 0.13) | (47 to 78) | (0.35 to 0.6) | (4.13 to 6.32) |
| Burkina Faso | 146 | 3.66 | 423 | 4.14 | 0.11% |
|  | (16 to 370) | (0.43 to 8.59) | (30 to 1016) | (0.29 to 9.71) | (-0.73 to 0.97) |
| Burundi | 1349 | 48.59 | 2174 | 36.94 | -0.86% |
|  | (699 to 2333) | (25.99 to 89.74) | (1016 to 3990) | (18.04 to 67.91) | (-1.02 to -0.7) |
| Cabo Verde | 1 | 0.29 | 8 | 2 | 5.97% |
|  | (0 to 1) | (0.14 to 0.56) | (4 to 15) | (0.83 to 3.44) | (5.46 to 6.49) |
| Cambodia | 1643 | 35.47 | 3646 | 33.33 | -0.24% |
|  | (818 to 2497) | (18.92 to 56.71) | (1989 to 5305) | (20.2 to 46.79) | (-0.27 to -0.21) |
| Cameroon | 8 | 0.2 | 116 | 0.49 | 2.86% |
|  | (1 to 17) | (0.03 to 0.43) | (12 to 264) | (0.06 to 1.04) | (2.27 to 3.46) |
| Canada | 1226 | 3.82 | 983 | 1.27 | -3.78% |
|  | (1105 to 1330) | (3.43 to 4.14) | (835 to 1103) | (1.1 to 1.42) | (-3.99 to -3.56) |
| Central African Republic | 365 | 30.58 | 655 | 27.9 | -0.13% |
|  | (134 to 626) | (12.35 to 51.52) | (224 to 1171) | (10.91 to 47.62) | (-0.23 to -0.02) |
| Chad | 4 | 0.15 | 36 | 0.36 | 2.67% |
|  | (1 to 9) | (0.04 to 0.31) | (6 to 75) | (0.08 to 0.69) | (2.12 to 3.21) |
| Chile | 568 | 6.31 | 4382 | 16.71 | 3.44% |
|  | (512 to 620) | (5.66 to 6.88) | (3824 to 4887) | (14.64 to 18.61) | (2.77 to 4.11) |
| China | 4422 | 0.93 | 44878 | 2.69 | 2.29% |
|  | (2205 to 13725) | (0.49 to 2.65) | (20365 to 58804) | (1.28 to 3.51) | (1.15 to 3.43) |
| Colombia | 7006 | 36.27 | 4263 | 7.63 | -3.76% |
|  | (6530 to 7459) | (33.67 to 38.64) | (3355 to 5342) | (5.98 to 9.6) | (-4.34 to -3.18) |
| Comoros | 95 | 42.07 | 198 | 38.48 | -0.39% |
|  | (45 to 172) | (20.69 to 74.84) | (95 to 341) | (18.98 to 66.38) | (-0.52 to -0.25) |
| Congo | 338 | 31.53 | 706 | 26.37 | -0.39% |
|  | (122 to 606) | (12.03 to 52.98) | (225 to 1243) | (8.95 to 46.13) | (-0.55 to -0.22) |
| Cook Islands | 1 | 7.63 | 2 | 8.26 | 0.12% |
|  | (1 to 1) | (4.57 to 11.91) | (1 to 3) | (3.86 to 14.01) | (-0.14 to 0.39) |
| Costa Rica | 297 | 16.58 | 673 | 12.1 | -0.90% |
|  | (265 to 326) | (14.73 to 18.24) | (574 to 761) | (10.38 to 13.66) | (-1.11 to -0.7) |
| Croatia | 25 | 0.45 | 114 | 1.28 | 3.86% |
|  | (21 to 29) | (0.38 to 0.53) | (94 to 137) | (1.07 to 1.54) | (2.93 to 4.81) |
| Cuba | 1140 | 11.61 | 1146 | 5.54 | -2.35% |
|  | (1041 to 1250) | (10.57 to 12.74) | (958 to 1320) | (4.64 to 6.35) | (-2.63 to -2.07) |
| Cyprus | 306 | 55.01 | 497 | 29.65 | -1.94% |
|  | (210 to 400) | (37.25 to 72.94) | (379 to 642) | (23.38 to 37.45) | (-2.14 to -1.73) |
| Czechia | 311 | 2.33 | 1361 | 6.49 | 3.47% |
|  | (276 to 346) | (2.1 to 2.56) | (1132 to 1622) | (5.51 to 7.65) | (2.29 to 4.66) |
| Democratic People's Republic of Korea | 898 | 6.75 | 1703 | 6.06 | 0.01% |
|  | (577 to 1355) | (4.5 to 9.68) | (1220 to 2403) | (4.4 to 8.32) | (-0.13 to 0.15) |
| Democratic Republic of the Congo | 4022 | 25.18 | 9501 | 25.49 | -0.08% |
|  | (1042 to 6893) | (6.87 to 41.57) | (3398 to 16153) | (9.25 to 42.71) | (-0.12 to -0.03) |
| Denmark | 203 | 2.34 | 127 | 0.9 | -2.50% |
|  | (181 to 220) | (2.1 to 2.53) | (104 to 147) | (0.74 to 1.04) | (-3.14 to -1.84) |
| Djibouti | 60 | 35.36 | 263 | 38.67 | 0.52% |
|  | (27 to 117) | (16.39 to 73.12) | (134 to 429) | (22.55 to 60.12) | (0.39 to 0.65) |
| Dominica | 68 | 115.24 | 92 | 120.18 | -0.11% |
|  | (51 to 90) | (87.72 to 153.79) | (72 to 117) | (93.73 to 153.42) | (-0.26 to 0.04) |
| Dominican Republic | 77 | 2.09 | 168 | 1.68 | -1.20% |
|  | (26 to 145) | (0.76 to 4.08) | (77 to 322) | (0.78 to 3.23) | (-1.51 to -0.88) |
| Ecuador | 90 | 1.69 | 1548 | 10.29 | 3.17% |
|  | (84 to 96) | (1.57 to 1.79) | (1208 to 2026) | (8.11 to 13.28) | (1.38 to 4.98) |
| Egypt | 4228 | 19.08 | 8586 | 16.98 | 0.18% |
|  | (2845 to 6816) | (12.32 to 34.68) | (6644 to 10462) | (13.37 to 20.45) | (0.01 to 0.35) |
| El Salvador | 448 | 13.43 | 751 | 11.43 | -1.84% |
|  | (268 to 597) | (8.22 to 18) | (471 to 1087) | (7.04 to 16.55) | (-2.19 to -1.49) |
| Equatorial Guinea | 55 | 27.02 | 128 | 23.38 | -0.40% |
|  | (21 to 96) | (10.7 to 43.17) | (36 to 252) | (7.2 to 46.79) | (-0.51 to -0.28) |
| Eritrea | 740 | 51.76 | 1613 | 52.22 | 0.15% |
|  | (389 to 1299) | (29.05 to 94.62) | (977 to 2655) | (31.56 to 83.76) | (0.09 to 0.21) |
| Estonia | 18 | 0.94 | 75 | 3.09 | 2.78% |
|  | (13 to 23) | (0.7 to 1.21) | (64 to 86) | (2.61 to 3.57) | (2.07 to 3.5) |
| Eswatini | 92 | 35.53 | 260 | 50.89 | 2.11% |
|  | (52 to 144) | (20.36 to 59.35) | (162 to 409) | (31.77 to 78.75) | (1.64 to 2.58) |
| Ethiopia | 11840 | 48.9 | 15183 | 28.32 | -1.93% |
|  | (6278 to 19365) | (28.21 to 81.87) | (8574 to 23005) | (16.88 to 41.5) | (-2.07 to -1.78) |
| Fiji | 49 | 13.64 | 99 | 16.48 | 0.18% |
|  | (26 to 85) | (7.7 to 22.76) | (47 to 157) | (9.3 to 23.91) | (-0.21 to 0.57) |
| Finland | 54 | 0.75 | 33 | 0.2 | -3.85% |
|  | (46 to 61) | (0.64 to 0.84) | (26 to 38) | (0.16 to 0.23) | (-4.41 to -3.29) |
| France | 21948 | 24.75 | 10208 | 5.35 | -4.26% |
|  | (19495 to 23662) | (22.13 to 26.57) | (8420 to 11496) | (4.5 to 5.95) | (-4.83 to -3.69) |
| Gabon | 160 | 28.35 | 255 | 25.88 | 0.12% |
|  | (56 to 257) | (10.58 to 45.45) | (75 to 490) | (7.65 to 47.9) | (-0.09 to 0.32) |
| Gambia | 1 | 0.16 | 8 | 0.45 | 2.91% |
|  | (0 to 1) | (0.03 to 0.34) | (1 to 17) | (0.06 to 0.93) | (2.26 to 3.56) |
| Georgia | 1 | 0.02 | 114 | 1.95 | 14.05% |
|  | (1 to 1) | (0.02 to 0.02) | (92 to 137) | (1.59 to 2.33) | (11.39 to 16.78) |
| Germany | 4245 | 3.22 | 8037 | 3.47 | 0.09% |
|  | (3790 to 4676) | (2.89 to 3.54) | (6725 to 9081) | (2.96 to 3.91) | (-0.4 to 0.58) |
| Ghana | 170 | 2.89 | 674 | 2.64 | -0.65% |
|  | (17 to 379) | (0.32 to 5.91) | (45 to 1797) | (0.22 to 10.04) | (-0.83 to -0.47) |
| Greece | 142 | 0.99 | 320 | 0.97 | -1.77% |
|  | (128 to 154) | (0.89 to 1.07) | (268 to 364) | (0.82 to 1.08) | (-2.1 to -1.44) |
| Greenland | 1 | 2.13 | 1 | 1.02 | -4.28% |
|  | (0 to 1) | (0.6 to 3.51) | (0 to 1) | (0.23 to 1.91) | (-5.1 to -3.46) |
| Grenada | 88 | 113.86 | 113 | 119.32 | 0.65% |
|  | (76 to 98) | (98.41 to 127.21) | (99 to 126) | (103.81 to 132.54) | (0.42 to 0.88) |
| Guam | 3 | 6.11 | 9 | 4.42 | -1.38% |
|  | (2 to 7) | (3.53 to 11.86) | (3 to 14) | (1.56 to 6.87) | (-2.06 to -0.7) |
| Guatemala | 1024 | 28.73 | 952 | 8.99 | -2.88% |
|  | (903 to 1153) | (25.31 to 31.71) | (799 to 1126) | (7.61 to 10.54) | (-3.48 to -2.28) |
| Guinea | 5 | 0.16 | 30 | 0.33 | 1.96% |
|  | (1 to 12) | (0.03 to 0.38) | (5 to 61) | (0.06 to 0.65) | (1.34 to 2.58) |
| Guinea-Bissau | 1 | 0.23 | 7 | 0.44 | 1.81% |
|  | (0 to 2) | (0.06 to 0.53) | (2 to 13) | (0.13 to 0.8) | (1.1 to 2.53) |
| Guyana | 28 | 6.98 | 87 | 15.2 | 2.76% |
|  | (25 to 31) | (6.31 to 7.69) | (68 to 110) | (11.94 to 19.08) | (1.9 to 3.64) |
| Haiti | 3047 | 87.43 | 6220 | 83.84 | -0.07% |
|  | (1900 to 4778) | (55.44 to 135.98) | (3760 to 9924) | (50.75 to 132.37) | (-0.15 to 0) |
| Honduras | 292 | 12.91 | 928 | 15.36 | 0.68% |
|  | (178 to 433) | (7.92 to 19.82) | (648 to 1250) | (10.96 to 20.47) | (0.55 to 0.81) |
| Hungary | 107 | 0.78 | 528 | 2.77 | 3.69% |
|  | (93 to 123) | (0.68 to 0.91) | (449 to 616) | (2.35 to 3.28) | (2.34 to 5.06) |
| Iceland | 2 | 0.64 | 2 | 0.22 | -3.14% |
|  | (2 to 2) | (0.56 to 0.72) | (1 to 2) | (0.18 to 0.26) | (-3.73 to -2.55) |
| India | 77567 | 13.45 | 138146 | 11.36 | -2.07% |
|  | (46215 to 110917) | (7.61 to 19.21) | (81979 to 184353) | (6.56 to 15.06) | (-2.9 to -1.24) |
| Indonesia | 7269 | 6.81 | 24193 | 11.84 | 1.07% |
|  | (3729 to 13456) | (3.72 to 12.51) | (10891 to 34824) | (5.8 to 16.17) | (0.86 to 1.28) |
| Iran (Islamic Republic of) | 2860 | 12.08 | 7744 | 11 | -0.45% |
|  | (1826 to 3787) | (8.21 to 16.55) | (5629 to 9002) | (8.24 to 12.71) | (-0.59 to -0.32) |
| Iraq | 255 | 3.15 | 554 | 2.92 | -1.16% |
|  | (126 to 364) | (1.63 to 4.58) | (304 to 802) | (1.74 to 4.14) | (-1.49 to -0.83) |
| Ireland | 451 | 11.28 | 225 | 2.62 | -3.55% |
|  | (421 to 480) | (10.45 to 12.01) | (181 to 264) | (2.11 to 3.06) | (-4.22 to -2.88) |
| Israel | 1983 | 43.75 | 5893 | 42.83 | 0.50% |
|  | (1804 to 2155) | (39.93 to 47.47) | (4922 to 6556) | (36.19 to 47.51) | (0.12 to 0.88) |
| Italy | 2818 | 3.3 | 6735 | 3.44 | 0.53% |
|  | (2519 to 2985) | (2.93 to 3.5) | (5470 to 7466) | (2.87 to 3.78) | (0.1 to 0.97) |
| Jamaica | 1305 | 70.74 | 2517 | 77.28 | 0.38% |
|  | (1094 to 1460) | (59.38 to 78.93) | (1971 to 3110) | (60.57 to 95.88) | (0.11 to 0.65) |
| Japan | 2868 | 2.02 | 7308 | 1.36 | -3.91% |
|  | (2519 to 3050) | (1.73 to 2.17) | (5928 to 8191) | (1.17 to 1.5) | (-4.87 to -2.94) |
| Jordan | 208 | 16.63 | 871 | 14.81 | -0.28% |
|  | (163 to 264) | (13.29 to 21.5) | (662 to 1094) | (11.67 to 18.44) | (-0.4 to -0.16) |
| Kazakhstan | 44 | 0.36 | 99 | 0.59 | 1.75% |
|  | (32 to 57) | (0.25 to 0.46) | (74 to 127) | (0.46 to 0.75) | (1.33 to 2.18) |
| Kenya | 3318 | 34.08 | 10120 | 40.92 | 0.63% |
|  | (1547 to 8261) | (15.31 to 89.16) | (5787 to 16971) | (24.9 to 69.94) | (0.55 to 0.71) |
| Kiribati | 41 | 103.89 | 79 | 108.3 | 0.26% |
|  | (16 to 89) | (42.91 to 227.33) | (36 to 153) | (52.37 to 205.89) | (0.18 to 0.34) |
| Kuwait | 11 | 1.93 | 81 | 3.59 | 0.91% |
|  | (9 to 12) | (1.63 to 2.32) | (62 to 101) | (2.73 to 4.52) | (-0.49 to 2.32) |
| Kyrgyzstan | 0 | 0 | 6 | 0.12 | 7.64% |
|  | (0 to 0) | (0 to 0.01) | (5 to 8) | (0.08 to 0.16) | (5.6 to 9.72) |
| Lao People's Democratic Republic | 799 | 38.78 | 1540 | 33.94 | -0.40% |
|  | (377 to 1318) | (19.56 to 69.07) | (731 to 2505) | (18.18 to 51.96) | (-0.41 to -0.38) |
| Latvia | 106 | 3.22 | 468 | 13.72 | 3.92% |
|  | (87 to 126) | (2.66 to 3.85) | (402 to 534) | (11.83 to 15.96) | (3.3 to 4.54) |
| Lebanon | 550 | 29.26 | 1258 | 19.13 | -0.94% |
|  | (378 to 741) | (20.44 to 39.35) | (912 to 1629) | (13.88 to 24.51) | (-1.17 to -0.72) |
| Lesotho | 161 | 20.58 | 428 | 44.31 | 3.22% |
|  | (84 to 267) | (10.91 to 34.52) | (270 to 620) | (28.49 to 62.74) | (2.84 to 3.59) |
| Liberia | 2 | 0.17 | 20 | 0.44 | 2.76% |
|  | (0 to 4) | (0.03 to 0.34) | (1 to 50) | (0.05 to 1.05) | (2 to 3.52) |
| Libya | 242 | 12.24 | 732 | 15.13 | 0.99% |
|  | (149 to 455) | (7.15 to 24.12) | (461 to 1035) | (9.56 to 21.46) | (0.79 to 1.2) |
| Lithuania | 36 | 0.86 | 181 | 3.66 | 4.05% |
|  | (26 to 46) | (0.62 to 1.09) | (154 to 212) | (3.1 to 4.31) | (3.33 to 4.77) |
| Luxembourg | 30 | 5.62 | 34 | 2.69 | -2.03% |
|  | (28 to 32) | (5.2 to 5.97) | (28 to 38) | (2.3 to 3.07) | (-2.43 to -1.64) |
| Madagascar | 2248 | 37.81 | 4748 | 34.44 | -0.31% |
|  | (1114 to 4944) | (17.25 to 91.3) | (2347 to 9268) | (17.22 to 71.76) | (-0.39 to -0.23) |
| Malawi | 2042 | 44.83 | 3892 | 43.61 | 0% |
|  | (1165 to 3159) | (26.5 to 72.12) | (2258 to 5849) | (26.73 to 63.82) | (-0.22 to 0.22) |
| Malaysia | 4841 | 50.85 | 25197 | 96.39 | 1.45% |
|  | (3705 to 6096) | (38.72 to 65.26) | (17580 to 32431) | (66.5 to 123.17) | (0.95 to 1.96) |
| Maldives | 34 | 40.35 | 56 | 17.64 | -3.47% |
|  | (21 to 47) | (23.35 to 59.59) | (33 to 80) | (10.93 to 24.65) | (-3.8 to -3.14) |
| Mali | 6 | 0.18 | 51 | 0.36 | 2% |
|  | (1 to 13) | (0.03 to 0.39) | (7 to 116) | (0.06 to 0.77) | (1.3 to 2.71) |
| Malta | 183 | 45.99 | 497 | 45.47 | 0.08% |
|  | (169 to 195) | (42.03 to 48.94) | (412 to 560) | (38.27 to 51.01) | (-0.34 to 0.51) |
| Marshall Islands | 5 | 26.93 | 12 | 32.53 | 0.98% |
|  | (2 to 8) | (12.95 to 47.79) | (6 to 19) | (15.91 to 49.89) | (0.86 to 1.1) |
| Mauritania | 2 | 0.16 | 10 | 0.35 | 2.23% |
|  | (0 to 3) | (0.03 to 0.33) | (1 to 24) | (0.04 to 0.76) | (1.61 to 2.86) |
| Mauritius | 78 | 10.54 | 542 | 31.88 | 3.66% |
|  | (73 to 84) | (9.91 to 11.28) | (493 to 577) | (28.98 to 33.88) | (3.25 to 4.07) |
| Mexico | 11465 | 27.29 | 12028 | 10.12 | -2.91% |
|  | (11167 to 11770) | (26.31 to 28.03) | (9348 to 15952) | (7.94 to 13.34) | (-3.16 to -2.65) |
| Micronesia (Federated States of) | 15 | 29.78 | 24 | 34.03 | 0.53% |
|  | (6 to 26) | (14.14 to 51.22) | (10 to 41) | (15.5 to 54.23) | (0.49 to 0.56) |
| Monaco | 2 | 2.57 | 3 | 2.53 | -0.72% |
|  | (1 to 3) | (1.69 to 3.39) | (2 to 4) | (1.74 to 3.5) | (-0.96 to -0.48) |
| Mongolia | 8 | 0.59 | 23 | 1.01 | -0.33% |
|  | (0 to 49) | (0.01 to 3.81) | (5 to 65) | (0.2 to 2.78) | (-1.06 to 0.41) |
| Montenegro | 0 | 0.01 | 0 | 0.01 | 0.44% |
|  | (0 to 0) | (0 to 0.01) | (0 to 0) | (0 to 0.02) | (0.07 to 0.83) |
| Morocco | 1542 | 10.52 | 5086 | 16.63 | 1.33% |
|  | (1025 to 2371) | (6.84 to 16.53) | (3508 to 6859) | (11.74 to 22.25) | (1.17 to 1.5) |
| Mozambique | 390 | 5.43 | 945 | 6.53 | 0.06% |
|  | (162 to 712) | (2.49 to 10.25) | (414 to 1632) | (3.22 to 11.15) | (-0.62 to 0.74) |
| Myanmar | 7867 | 33.84 | 13067 | 29.49 | -0.46% |
|  | (3867 to 12243) | (17.42 to 55.32) | (7603 to 18228) | (17.7 to 40.33) | (-0.54 to -0.38) |
| Namibia | 141 | 25.41 | 405 | 33.73 | 1.40% |
|  | (87 to 213) | (16.39 to 38.36) | (266 to 580) | (22.91 to 48.37) | (1.05 to 1.75) |
| Nauru | 2 | 32.97 | 3 | 42.18 | 0.95% |
|  | (1 to 3) | (16.83 to 54.32) | (1 to 4) | (21.02 to 66.58) | (0.85 to 1.06) |
| Nepal | 68 | 0.54 | 416 | 1.32 | 2.23% |
|  | (51 to 94) | (0.41 to 0.7) | (258 to 639) | (0.86 to 1.98) | (1.5 to 2.96) |
| Netherlands | 4425 | 21.73 | 2866 | 7.15 | -2.88% |
|  | (3845 to 4769) | (18.85 to 23.4) | (2364 to 3275) | (5.98 to 8.15) | (-3.41 to -2.35) |
| New Zealand | 124 | 3.26 | 100 | 1.06 | -3.99% |
|  | (108 to 138) | (2.84 to 3.65) | (80 to 117) | (0.85 to 1.23) | (-4.96 to -3.02) |
| Nicaragua | 184 | 10.22 | 367 | 7.75 | -1.45% |
|  | (110 to 236) | (6.34 to 13.34) | (214 to 502) | (4.8 to 10.57) | (-1.59 to -1.3) |
| Niger | 4 | 0.16 | 42 | 0.31 | 1.80% |
|  | (1 to 9) | (0.03 to 0.35) | (5 to 101) | (0.05 to 0.73) | (1.18 to 2.44) |
| Nigeria | 63 | 0.15 | 453 | 0.3 | 2.37% |
|  | (20 to 116) | (0.05 to 0.28) | (106 to 828) | (0.07 to 0.53) | (1.86 to 2.87) |
| Niue | 1 | 24.48 | 1 | 29.48 | 0.65% |
|  | (0 to 1) | (12.82 to 38.95) | (0 to 1) | (14.46 to 44.76) | (0.57 to 0.73) |
| North Macedonia | 0 | 0 | 0 | 0 | 3.50% |
|  | (0 to 0) | (0 to 0) | (0 to 0) | (0 to 0.01) | (3.03 to 3.97) |
| Northern Mariana Islands | 7 | 33.72 | 6 | 16.76 | -3.78% |
|  | (4 to 11) | (20.6 to 52.19) | (3 to 10) | (8.01 to 26.85) | (-4.49 to -3.08) |
| Norway | 326 | 4.21 | 232 | 1.84 | -1.13% |
|  | (289 to 345) | (3.74 to 4.45) | (194 to 258) | (1.55 to 2.03) | (-1.47 to -0.79) |
| Oman | 252 | 35.44 | 719 | 39.62 | 0.07% |
|  | (162 to 368) | (23.29 to 53.29) | (394 to 1002) | (25.11 to 53.66) | (-0.45 to 0.58) |
| Pakistan | 3899 | 4.83 | 6899 | 4.1 | -0.22% |
|  | (1924 to 6283) | (2.09 to 8.43) | (2963 to 12036) | (1.44 to 7.57) | (-0.39 to -0.05) |
| Palau | 0 | 0.74 | 0 | 0.68 | -1.91% |
|  | (0 to 0) | (0.17 to 1.95) | (0 to 0) | (0.15 to 1.82) | (-2.59 to -1.21) |
| Palestine | 92 | 11.6 | 233 | 11.43 | -0.62% |
|  | (58 to 139) | (7.36 to 17.86) | (144 to 292) | (7.6 to 14.04) | (-0.87 to -0.37) |
| Panama | 289 | 18.32 | 892 | 19.68 | 0.59% |
|  | (251 to 324) | (15.85 to 20.63) | (687 to 1066) | (15.11 to 23.46) | (0.42 to 0.77) |
| Papua New Guinea | 410 | 21 | 1227 | 21.67 | -0.01% |
|  | (121 to 1121) | (6.36 to 57.88) | (333 to 2947) | (6.51 to 50.5) | (-0.09 to 0.07) |
| Paraguay | 161 | 7.09 | 509 | 8.94 | 0.09% |
|  | (83 to 332) | (3.72 to 14.42) | (280 to 749) | (5 to 13.17) | (-0.19 to 0.37) |
| Peru | 1962 | 14.8 | 1609 | 4.74 | -4.48% |
|  | (1263 to 2499) | (9.94 to 18.7) | (866 to 2668) | (2.55 to 7.97) | (-5.1 to -3.85) |
| Philippines | 12891 | 42.16 | 28636 | 39.59 | -0.34% |
|  | (8697 to 17767) | (30.5 to 57.4) | (20928 to 35376) | (30.07 to 48.21) | (-0.42 to -0.27) |
| Poland | 316 | 0.73 | 1 | 0 | -16.52% |
|  | (298 to 335) | (0.69 to 0.78) | (1 to 1) | (0 to 0) | (-17.97 to -15.04) |
| Portugal | 779 | 6.14 | 848 | 2.73 | -2.11% |
|  | (716 to 828) | (5.64 to 6.51) | (712 to 963) | (2.33 to 3.06) | (-3.18 to -1.03) |
| Puerto Rico | 1064 | 31.45 | 984 | 12.28 | -2.55% |
|  | (994 to 1119) | (29.38 to 33.18) | (805 to 1166) | (10.07 to 14.55) | (-2.95 to -2.15) |
| Qatar | 13 | 18.07 | 87 | 18.25 | -1.40% |
|  | (8 to 21) | (10.13 to 28.87) | (34 to 149) | (8.32 to 29.07) | (-1.89 to -0.9) |
| Republic of Korea | 3093 | 13.73 | 5641 | 6.31 | -2.69% |
|  | (2204 to 3714) | (10.15 to 16.71) | (4013 to 7213) | (4.54 to 7.99) | (-2.86 to -2.52) |
| Republic of Moldova | 2 | 0.05 | 15 | 0.27 | 4.30% |
|  | (2 to 3) | (0.04 to 0.07) | (12 to 18) | (0.23 to 0.32) | (3.56 to 5.04) |
| Romania | 148 | 0.59 | 756 | 2.16 | 3.19% |
|  | (115 to 184) | (0.46 to 0.72) | (655 to 882) | (1.85 to 2.53) | (2.1 to 4.3) |
| Russian Federation | 699 | 0.41 | 7181 | 3.34 | 5.22% |
|  | (649 to 743) | (0.38 to 0.44) | (6601 to 7792) | (3.08 to 3.62) | (4.53 to 5.91) |
| Rwanda | 1737 | 50.14 | 2335 | 34.07 | -1.17% |
|  | (960 to 2934) | (28.73 to 86.45) | (1009 to 4401) | (15.41 to 61.7) | (-1.47 to -0.86) |
| Saint Kitts and Nevis | 46 | 127.1 | 50 | 92.11 | -0.56% |
|  | (40 to 51) | (112.11 to 141.27) | (42 to 59) | (78.22 to 105.74) | (-0.86 to -0.26) |
| Saint Lucia | 59 | 74.13 | 170 | 75.57 | 0.35% |
|  | (55 to 65) | (68.8 to 81.1) | (142 to 201) | (63.21 to 88.85) | (-0.13 to 0.82) |
| Saint Vincent and the Grenadines | 21 | 29.19 | 134 | 103.43 | 4.43% |
|  | (20 to 23) | (27.26 to 31.08) | (118 to 153) | (90.82 to 117.75) | (3.52 to 5.34) |
| Samoa | 19 | 23.58 | 40 | 28.12 | 0.61% |
|  | (10 to 33) | (12.33 to 38.2) | (17 to 62) | (13.46 to 42.96) | (0.53 to 0.68) |
| San Marino | 0 | 1.21 | 1 | 0.56 | -2.33% |
|  | (0 to 1) | (0.76 to 1.63) | (0 to 1) | (0.31 to 0.86) | (-2.58 to -2.08) |
| Sao Tome and Principe | 0 | 0.02 | 0 | 0.03 | 1.98% |
|  | (0 to 0) | (0 to 0.03) | (0 to 0) | (0.01 to 0.05) | (1.8 to 2.17) |
| Saudi Arabia | 5135 | 81.89 | 16387 | 88.21 | 0.50% |
|  | (3312 to 8096) | (53.15 to 127.71) | (12757 to 20868) | (71.88 to 108.68) | (0.39 to 0.62) |
| Senegal | 6 | 0.18 | 43 | 0.38 | 2.28% |
|  | (1 to 11) | (0.03 to 0.35) | (5 to 91) | (0.05 to 0.76) | (1.63 to 2.94) |
| Serbia | 216 | 2.27 | 485 | 3.02 | 0.66% |
|  | (126 to 385) | (1.31 to 3.92) | (208 to 735) | (1.31 to 4.54) | (0.2 to 1.11) |
| Seychelles | 8 | 13.33 | 14 | 13.87 | 0.12% |
|  | (4 to 11) | (7.46 to 18.55) | (7 to 19) | (7.5 to 18.15) | (-0.11 to 0.34) |
| Sierra Leone | 3 | 0.16 | 19 | 0.31 | 1.55% |
|  | (1 to 6) | (0.04 to 0.31) | (4 to 43) | (0.07 to 0.65) | (0.9 to 2.2) |
| Singapore | 423 | 22.3 | 755 | 9.28 | -4.03% |
|  | (395 to 449) | (20.56 to 23.72) | (630 to 866) | (7.74 to 10.64) | (-4.55 to -3.5) |
| Slovakia | 169 | 2.91 | 395 | 4.51 | 0.96% |
|  | (119 to 248) | (2.04 to 4.3) | (223 to 546) | (2.53 to 6.28) | (0.42 to 1.5) |
| Slovenia | 41 | 1.7 | 192 | 4.34 | 3.06% |
|  | (37 to 45) | (1.54 to 1.87) | (163 to 218) | (3.69 to 4.93) | (2.23 to 3.91) |
| Solomon Islands | 36 | 24.97 | 122 | 30.84 | 0.67% |
|  | (11 to 75) | (8.23 to 50.89) | (36 to 229) | (10.23 to 57.01) | (0.64 to 0.71) |
| Somalia | 1645 | 51.94 | 3896 | 48.76 | -0.04% |
|  | (686 to 3362) | (21.83 to 118.59) | (1924 to 7357) | (25.66 to 97.67) | (-0.11 to 0.03) |
| South Africa | 5170 | 25.64 | 9672 | 24.38 | -0.74% |
|  | (2836 to 7104) | (14.54 to 36.12) | (6673 to 12204) | (17.34 to 30.81) | (-0.89 to -0.58) |
| South Sudan | 1247 | 42.94 | 2369 | 50.01 | 0.24% |
|  | (525 to 2585) | (18.25 to 91.41) | (1038 to 4497) | (22.7 to 96.33) | (0.11 to 0.36) |
| Spain | 4939 | 9.34 | 6914 | 5.05 | -0.37% |
|  | (4483 to 5255) | (8.45 to 9.93) | (5486 to 7878) | (4.1 to 5.71) | (-1.01 to 0.28) |
| Sri Lanka | 536 | 5.32 | 1543 | 6.4 | -0.14% |
|  | (332 to 811) | (3.36 to 7.93) | (816 to 2372) | (3.51 to 9.67) | (-0.58 to 0.3) |
| Sudan | 1145 | 11.25 | 3380 | 17.18 | 1.31% |
|  | (686 to 1881) | (6.46 to 19.67) | (1710 to 6259) | (9.33 to 31.87) | (1.15 to 1.46) |
| Suriname | 296 | 111.07 | 638 | 105.38 | -0.52% |
|  | (217 to 360) | (83.88 to 135.45) | (451 to 860) | (75.23 to 141.57) | (-0.77 to -0.26) |
| Sweden | 966 | 5.77 | 311 | 1.13 | -2.23% |
|  | (871 to 1042) | (5.21 to 6.22) | (255 to 355) | (0.94 to 1.28) | (-3.68 to -0.76) |
| Switzerland | 232 | 2.07 | 238 | 0.97 | -1.95% |
|  | (206 to 256) | (1.86 to 2.26) | (184 to 282) | (0.76 to 1.14) | (-2.43 to -1.46) |
| Syrian Arab Republic | 24 | 0.48 | 37 | 0.37 | -0.43% |
|  | (15 to 33) | (0.31 to 0.68) | (24 to 52) | (0.25 to 0.49) | (-0.7 to -0.15) |
| Taiwan (Province of China) | 6119 | 44.72 | 4808 | 11.14 | -2.60% |
|  | (5742 to 6441) | (41.45 to 47.31) | (4138 to 5440) | (9.62 to 12.6) | (-3.48 to -1.71) |
| Tajikistan | 1 | 0.03 | 1 | 0.01 | -3.12% |
|  | (0 to 3) | (0.01 to 0.08) | (0 to 3) | (0 to 0.04) | (-3.68 to -2.56) |
| Thailand | 10153 | 28.15 | 31385 | 30.57 | -0.48% |
|  | (5011 to 18726) | (14.5 to 50.74) | (14288 to 45776) | (13.82 to 44.61) | (-1.05 to 0.09) |
| Timor-Leste | 80 | 25.89 | 230 | 27.53 | -0.01% |
|  | (34 to 144) | (12.33 to 46.44) | (99 to 395) | (13.12 to 45.77) | (-0.16 to 0.14) |
| Togo | 2 | 0.16 | 21 | 0.33 | 2.38% |
|  | (0 to 4) | (0.02 to 0.33) | (5 to 40) | (0.08 to 0.59) | (1.85 to 2.92) |
| Tokelau | 0 | 24.56 | 0 | 26.05 | 0.03% |
|  | (0 to 1) | (12.33 to 41.5) | (0 to 1) | (13.53 to 40.43) | (-0.02 to 0.07) |
| Tonga | 10 | 17.81 | 17 | 21.14 | 0.65% |
|  | (4 to 18) | (7.78 to 33.88) | (7 to 30) | (9.57 to 36.5) | (0.55 to 0.75) |
| Trinidad and Tobago | 283 | 38.97 | 341 | 18.65 | -2.24% |
|  | (261 to 305) | (35.71 to 42.51) | (268 to 423) | (14.65 to 23.09) | (-2.48 to -2) |
| Tunisia | 424 | 9.02 | 1960 | 16.37 | 1.67% |
|  | (288 to 621) | (6.12 to 13.52) | (938 to 3777) | (8.07 to 31.07) | (1.41 to 1.94) |
| Turkmenistan | 1 | 0.05 | 3 | 0.06 | -0.41% |
|  | (1 to 2) | (0.04 to 0.08) | (2 to 4) | (0.04 to 0.09) | (-0.75 to -0.07) |
| Tuvalu | 2 | 28.02 | 3 | 29.22 | 0.16% |
|  | (1 to 3) | (13.84 to 45.56) | (1 to 4) | (13.67 to 44.19) | (0.12 to 0.2) |
| Uganda | 2590 | 34.52 | 6516 | 35.93 | 0.07% |
|  | (1070 to 6573) | (14.05 to 91.14) | (2956 to 12230) | (17.87 to 65.68) | (-0.09 to 0.24) |
| Ukraine | 258 | 0.36 | 415 | 0.67 | 4.25% |
|  | (195 to 330) | (0.27 to 0.46) | (297 to 550) | (0.48 to 0.89) | (3.12 to 5.4) |
| United Arab Emirates | 32 | 9.87 | 93 | 6.62 | -0.70% |
|  | (14 to 50) | (4.87 to 15.6) | (38 to 167) | (3.04 to 10.89) | (-1.21 to -0.19) |
| United Kingdom | 7381 | 7.92 | 2161 | 1.46 | -4.90% |
|  | (6849 to 7678) | (7.35 to 8.24) | (1876 to 2317) | (1.29 to 1.56) | (-5.58 to -4.22) |
| United Republic of Tanzania | 5463 | 43.9 | 11016 | 37.67 | -0.45% |
|  | (2528 to 12125) | (20.54 to 97.76) | (5664 to 23089) | (20.53 to 76.88) | (-0.52 to -0.38) |
| United States of America | 26495 | 8.07 | 23243 | 3.98 | -2.16% |
|  | (24280 to 27803) | (7.43 to 8.46) | (20189 to 26445) | (3.48 to 4.51) | (-2.36 to -1.96) |
| United States Virgin Islands | 35 | 47.21 | 58 | 38.84 | -0.52% |
|  | (25 to 45) | (35.07 to 59.6) | (40 to 84) | (26.84 to 56.02) | (-0.66 to -0.38) |
| Uruguay | 981 | 25.32 | 2113 | 34.13 | 1.41% |
|  | (869 to 1079) | (22.33 to 27.86) | (1877 to 2298) | (30.8 to 37.13) | (0.99 to 1.83) |
| Uzbekistan | 0 | 0 | 0 | 0 | 1.20% |
|  | (0 to 0) | (0 to 0) | (0 to 0) | (0 to 0) | (0.89 to 1.51) |
| Vanuatu | 17 | 24.88 | 54 | 29.87 | 0.48% |
|  | (6 to 38) | (8.84 to 56.97) | (14 to 115) | (9.02 to 61.65) | (0.42 to 0.53) |
| Venezuela (Bolivarian Republic of) | 240 | 2.44 | 414 | 1.46 | -1.56% |
|  | (215 to 265) | (2.19 to 2.66) | (313 to 531) | (1.11 to 1.87) | (-1.95 to -1.17) |
| Viet Nam | 2463 | 6.73 | 6208 | 7.35 | -0.56% |
|  | (513 to 6230) | (1.3 to 17.51) | (1576 to 10245) | (1.89 to 12.4) | (-1.13 to 0.01) |
| Yemen | 530 | 10.27 | 1593 | 11.67 | 0.30% |
|  | (302 to 957) | (5.51 to 21.64) | (936 to 2519) | (7.03 to 18.73) | (0.23 to 0.38) |
| Zambia | 1870 | 51.55 | 3833 | 45.05 | -0.75% |
|  | (1131 to 2766) | (32.18 to 72.6) | (2161 to 6162) | (26.03 to 71.11) | (-1.19 to -0.32) |
| Zimbabwe | 1493 | 46.34 | 3896 | 68.59 | 1.29% |
|  | (466 to 2590) | (15.21 to 81.2) | (1267 to 6221) | (25.41 to 110.87) | (0.8 to 1.78) |

| Table S9: Estimated average percentage changes (EAPC) in age-standardized rate of prevalence, death and disability adjusted life-year (DALY) for decubitus ulcer from 1990 to 2021 and 2019 to 2021 at the global, SDI quintile and GBD region levels. | | | | | | | | | | | | |
| --- | --- | --- | --- | --- | --- | --- | --- | --- | --- | --- | --- | --- |
| Location | Age-standardized prevalence rate (95% UI) | | |  | Age-standardized death rate (95% UI) | | |  | Age-standardized DALY rate (95% UI) | | | |
|  | 1990-2021 | 1990-2019 | 2019-2021 |  | 1990-2021 | 1990-2019 | 2019-2021 |  | 1990-2021 | 1990-2019 | 2019-2021 |  |
| Global | -0.02% | 0.00% | -0.49% |  | -0.58% | -0.63% | -0.26% |  | -0.58% | -0.72% | 0.32% |  |
|  | (-0.1 to 0.06) | (-0.09 to 0.09) | (-0.93 to -0.06) |  | (-0.71 to -0.46) | (-0.76 to -0.5) | (-0.78 to 0.27) |  | (-0.77 to -0.39) | (-0.89 to -0.54) | (-0.17 to 0.82) |  |
| SDI quintile |  |  |  |  |  |  |  |  |  |  |  |  |
| High SDI | -0.18% | -0.20% | -0.53% |  | -2.47% | -2.49% | -2.02% |  | -1.89% | -2.01% | -1.28% |  |
|  | (-0.23 to -0.13) | (-0.25 to -0.15) | (-1.31 to 0.26) |  | (-2.73 to -2.2) | (-2.78 to -2.2) | (-4.15 to 0.17) |  | (-2.05 to -1.73) | (-2.16 to -1.86) | (-3.49 to 0.97) |  |
| High-middle SDI | 0.28% | 0.37% | 0.05% |  | 2.09% | 2.25% | -1.03% |  | 1.59% | 1.74% | -0.21% |  |
|  | (0.15 to 0.42) | (0.23 to 0.5) | (0.03 to 0.07) |  | (1.75 to 2.44) | (1.89 to 2.6) | (-2 to -0.06) |  | (1.19 to 1.99) | (1.3 to 2.17) | (-1.34 to 0.95) |  |
| Middle SDI | 0.85% | 0.91% | -0.02% |  | -0.18% | -0.21% | 0.12% |  | -0.23% | -0.31% | 0.94% |  |
|  | (0.71 to 0.99) | (0.76 to 1.06) | (-0.11 to 0.06) |  | (-0.24 to -0.12) | (-0.27 to -0.15) | (0.09 to 0.16) |  | (-0.33 to -0.12) | (-0.41 to -0.22) | (0.82 to 1.05) |  |
| Low-middle SDI | 1.16% | 1.26% | -0.05% |  | -0.28% | -0.46% | 0.48% |  | -0.46% | -0.78% | 0.59% |  |
|  | (0.98 to 1.33) | (1.08 to 1.43) | (-0.12 to 0.02) |  | (-0.58 to 0.02) | (-0.75 to -0.17) | (-1.2 to 2.2) |  | (-1 to 0.09) | (-1.33 to -0.23) | (-0.74 to 1.94) |  |
| Low SDI | 0.44% | 0.51% | -0.11% |  | -0.50% | -0.58% | 0.19% |  | -0.88% | -1.03% | 0.38% |  |
|  | (0.29 to 0.59) | (0.35 to 0.67) | (-0.22 to -0.01) |  | (-0.64 to -0.36) | (-0.72 to -0.44) | (-0.4 to 0.78) |  | (-1.11 to -0.66) | (-1.26 to -0.81) | (0.05 to 0.71) |  |
| GBD region |  |  |  |  |  |  |  |  |  |  |  |  |
| Andean Latin America | -0.57% | -0.56% | 0.07% |  | -1.94% | -2.23% | -2.68% |  | -2.44% | -2.97% | -1.58% |  |
|  | (-0.62 to -0.52) | (-0.62 to -0.5) | (0.04 to 0.1) |  | (-2.56 to -1.32) | (-2.86 to -1.59) | (-9.35 to 4.48) |  | (-3.3 to -1.57) | (-3.83 to -2.11) | (-9.95 to 7.57) |  |
| Australasia | -0.42% | -0.38% | -0.03% |  | -2.56% | -2.65% | -1.86% |  | -3.35% | -3.65% | -1.11% |  |
|  | (-0.53 to -0.32) | (-0.49 to -0.26) | (-0.27 to 0.21) |  | (-3.28 to -1.84) | (-3.44 to -1.86) | (-4.43 to 0.78) |  | (-3.95 to -2.74) | (-4.28 to -3.01) | (-2.86 to 0.67) |  |
| Caribbean | -0.48% | -0.49% | 0.02% |  | -1.28% | -1.31% | -0.94% |  | -0.83% | -0.91% | -0.54% |  |
|  | (-0.5 to -0.46) | (-0.51 to -0.46) | (-0.34 to 0.38) |  | (-1.38 to -1.18) | (-1.42 to -1.21) | (-1.54 to -0.33) |  | (-0.97 to -0.7) | (-1.05 to -0.76) | (-1.02 to -0.05) |  |
| Central Asia | 0% | 0.02% | 0.14% |  | 3.18% | 2.98% | -3.93% |  | 2.07% | 1.97% | -3.50% |  |
|  | (-0.04 to 0.04) | (-0.02 to 0.07) | (-0.06 to 0.34) |  | (2.43 to 3.94) | (2.17 to 3.8) | (-17.51 to 11.88) |  | (1.46 to 2.69) | (1.29 to 2.65) | (-15.78 to 10.57) |  |
| Central Europe | 0.40% | 0.46% | 0.42% |  | 1.97% | 1.81% | -2.74% |  | 1.76% | 1.82% | -1.25% |  |
|  | (0.33 to 0.48) | (0.38 to 0.53) | (-0.1 to 0.94) |  | (1.06 to 2.9) | (0.81 to 2.83) | (-8.52 to 3.41) |  | (1.41 to 2.11) | (1.43 to 2.21) | (-4.67 to 2.3) |  |
| Central Latin America | -0.25% | -0.26% | 0.05% |  | -2.53% | -2.50% | -7.21% |  | -2.59% | -2.63% | -5.34% |  |
|  | (-0.29 to -0.21) | (-0.3 to -0.22) | (-0.01 to 0.1) |  | (-2.74 to -2.33) | (-2.72 to -2.27) | (-10.89 to -3.37) |  | (-2.75 to -2.43) | (-2.81 to -2.45) | (-8.54 to -2.03) |  |
| Central Sub-Saharan Africa | -0.38% | -0.38% | 0.04% |  | -0.23% | -0.29% | 1.11% |  | -0.46% | -0.58% | 1.12% |  |
|  | (-0.41 to -0.36) | (-0.41 to -0.36) | (-0.24 to 0.31) |  | (-0.34 to -0.13) | (-0.39 to -0.19) | (0.6 to 1.63) |  | (-0.65 to -0.28) | (-0.76 to -0.39) | (0.69 to 1.55) |  |
| East Asia | 1.03% | 1.09% | 0.11% |  | 1.28% | 1.53% | 2.19% |  | 1.03% | 1.36% | 2.08% |  |
|  | (0.82 to 1.24) | (0.85 to 1.32) | (-0.13 to 0.34) |  | (0.59 to 1.99) | (0.78 to 2.28) | (1.91 to 2.47) |  | (0.17 to 1.91) | (0.41 to 2.32) | (1.93 to 2.23) |  |
| Eastern Europe | -0.37% | -0.32% | 0.06% |  | 4.79% | 4.50% | -0.69% |  | 2.57% | 2.38% | -0.58% |  |
|  | (-0.45 to -0.28) | (-0.42 to -0.23) | (-0.07 to 0.18) |  | (4.18 to 5.39) | (3.88 to 5.11) | (-1.16 to -0.23) |  | (2.14 to 3) | (1.92 to 2.84) | (-0.82 to -0.34) |  |
| Eastern Sub-Saharan Africa | -0.52% | -0.49% | 0.11% |  | -0.38% | -0.40% | 0.24% |  | -0.81% | -0.88% | 0.42% |  |
|  | (-0.66 to -0.39) | (-0.64 to -0.34) | (-0.12 to 0.34) |  | (-0.45 to -0.3) | (-0.48 to -0.32) | (-0.28 to 0.75) |  | (-0.91 to -0.71) | (-0.97 to -0.78) | (0.19 to 0.65) |  |
| High-income Asia Pacific | 0.15% | 0.20% | 0% |  | -3.14% | -3.51% | 0.71% |  | -0.93% | -1.13% | 0.52% |  |
|  | (0.06 to 0.25) | (0.1 to 0.3) | (-0.06 to 0.05) |  | (-3.67 to -2.61) | (-4 to -3.01) | (-1.14 to 2.59) |  | (-1.22 to -0.64) | (-1.41 to -0.85) | (-0.34 to 1.38) |  |
| High-income North America | -0.08% | -0.13% | -0.78% |  | -2.35% | -2.34% | -4.34% |  | -1.17% | -1.20% | -2.69% |  |
|  | (-0.18 to 0.01) | (-0.23 to -0.02) | (-1.89 to 0.34) |  | (-2.57 to -2.12) | (-2.58 to -2.09) | (-9.61 to 1.24) |  | (-1.26 to -1.08) | (-1.3 to -1.11) | (-6.49 to 1.26) |  |
| North Africa and Middle East | -0.02% | 0.08% | -0.06% |  | 0.44% | 0.42% | 0.49% |  | 0.30% | 0.27% | 0.56% |  |
|  | (-0.18 to 0.14) | (-0.08 to 0.25) | (-0.12 to 0.01) |  | (0.39 to 0.5) | (0.37 to 0.48) | (0.49 to 0.5) |  | (0.24 to 0.35) | (0.22 to 0.33) | (0.53 to 0.58) |  |
| Oceania | 0.09% | 0.09% | 0.15% |  | 0.01% | -0.01% | -0.30% |  | 0.16% | 0.14% | -0.63% |  |
|  | (0.09 to 0.1) | (0.09 to 0.1) | (-0.23 to 0.53) |  | (-0.04 to 0.06) | (-0.06 to 0.04) | (-0.35 to -0.25) |  | (0.09 to 0.24) | (0.06 to 0.23) | (-1.01 to -0.25) |  |
| South Asia | 1.24% | 1.33% | 0.21% |  | -1.20% | -1.60% | 1.70% |  | -1.33% | -1.98% | 1.48% |  |
|  | (0.99 to 1.49) | (1.05 to 1.6) | (0.05 to 0.38) |  | (-1.89 to -0.5) | (-2.28 to -0.91) | (1.07 to 2.33) |  | (-2.48 to -0.16) | (-3.16 to -0.79) | (1.15 to 1.81) |  |
| Southeast Asia | 0.71% | 0.71% | 0.35% |  | 0.71% | 0.60% | 4.04% |  | 0.75% | 0.63% | 4.19% |  |
|  | (0.63 to 0.79) | (0.61 to 0.8) | (-0.1 to 0.81) |  | (0.51 to 0.91) | (0.4 to 0.8) | (0.64 to 7.56) |  | (0.56 to 0.94) | (0.44 to 0.81) | (0.91 to 7.59) |  |
| Southern Latin America | 1.09% | 1.26% | -0.03% |  | 3.10% | 3.31% | -6.05% |  | 3.18% | 3.58% | -5.64% |  |
|  | (0.84 to 1.33) | (1.03 to 1.49) | (-0.07 to 0) |  | (2.58 to 3.62) | (2.76 to 3.86) | (-6.69 to -5.41) |  | (2.35 to 4.01) | (2.7 to 4.46) | (-6.74 to -4.54) |  |
| Southern Sub-Saharan Africa | -0.39% | -0.33% | -0.04% |  | 0.06% | 0.06% | 0.51% |  | 0.02% | 0.00% | 1.63% |  |
|  | (-0.53 to -0.25) | (-0.48 to -0.19) | (-0.34 to 0.27) |  | (-0.13 to 0.24) | (-0.14 to 0.26) | (-1.97 to 3.04) |  | (-0.24 to 0.29) | (-0.3 to 0.31) | (-0.19 to 3.49) |  |
| Tropical Latin America | 2.65% | 2.83% | -0.01% |  | 0.92% | 0.95% | -5.67% |  | 0.84% | 0.87% | -4.08% |  |
|  | (2.34 to 2.95) | (2.53 to 3.14) | (-0.08 to 0.05) |  | (0.79 to 1.06) | (0.81 to 1.09) | (-14.93 to 4.59) |  | (0.69 to 0.98) | (0.71 to 1.02) | (-12.27 to 4.88) |  |
| Western Europe | 0.19% | 0.39% | -0.09% |  | -2.02% | -1.96% | -3.18% |  | -2.99% | -3.07% | -2.86% |  |
|  | (-0.15 to 0.54) | (0.03 to 0.75) | (-0.12 to -0.05) |  | (-2.39 to -1.64) | (-2.38 to -1.55) | (-5.69 to -0.61) |  | (-3.23 to -2.74) | (-3.34 to -2.79) | (-5.9 to 0.28) |  |
| Western Sub-Saharan Africa | 0.11% | 0.15% | -0.01% |  | -0.27% | -0.39% | -0.83% |  | 1.62% | 1.64% | -0.43% |  |
|  | (0.04 to 0.17) | (0.08 to 0.21) | (-0.05 to 0.03) |  | (-0.56 to 0.02) | (-0.69 to -0.08) | (-0.97 to -0.7) |  | (1.29 to 1.94) | (1.27 to 2.01) | (-0.49 to -0.36) |  |

| Table S10: Estimated average percentage changes in age-standardized rates of years lived with disability (YLD) and years of life lost (YLL) for decubitus ulcers from 1990 to 2021 and 2019 to 2021 at the global, SDI quintile and GBD region levels. | | | | | | | |
| --- | --- | --- | --- | --- | --- | --- | --- |
| Location | Age-standardized YLD rate (95% UI) | | |  | Age-standardized YLL rate (95% UI) | | |
|  | 1990-2021 | 1990-2019 | 2019-2021 |  | 1990-2021 | 1990-2019 | 2019-2021 |
| Global | -0.04% | -0.02% | -0.48% |  | -0.64% | -0.73% | 0.44% |
|  | (-0.12 to 0.04) | (-0.1 to 0.07) | (-0.84 to -0.12) |  | (-0.77 to -0.51) | (-0.86 to -0.6) | (-0.07 to 0.96) |
| SDI quintile |  |  |  |  |  |  |  |
| High SDI | -0.22% | -0.23% | -0.52% |  | -2.11% | -2.16% | -1.66% |
|  | (-0.26 to -0.18) | (-0.28 to -0.18) | (-1.19 to 0.15) |  | (-2.31 to -1.92) | (-2.37 to -1.95) | (-4.61 to 1.37) |
| High-middle SDI | 0.19% | 0.26% | 0% |  | 1.85% | 1.94% | -0.24% |
|  | (0.06 to 0.31) | (0.14 to 0.39) | (0 to 0.01) |  | (1.58 to 2.12) | (1.65 to 2.23) | (-1.54 to 1.09) |
| Middle SDI | 0.78% | 0.84% | -0.02% |  | -0.48% | -0.55% | 1.06% |
|  | (0.65 to 0.91) | (0.69 to 0.98) | (-0.07 to 0.03) |  | (-0.56 to -0.39) | (-0.62 to -0.48) | (0.93 to 1.2) |
| Low-middle SDI | 1.11% | 1.21% | -0.07% |  | -0.74% | -0.95% | 0.63% |
|  | (0.94 to 1.29) | (1.03 to 1.39) | (-0.11 to -0.03) |  | (-1.08 to -0.4) | (-1.28 to -0.62) | (-0.77 to 2.05) |
| Low SDI | 0.45% | 0.52% | -0.08% |  | -0.78% | -0.85% | 0.39% |
|  | (0.3 to 0.6) | (0.36 to 0.68) | (-0.14 to -0.03) |  | (-0.92 to -0.64) | (-0.99 to -0.71) | (0.05 to 0.73) |
| GBD region |  |  |  |  |  |  |  |
| Andean Latin America | -0.47% | -0.46% | 0.25% |  | -2.38% | -2.69% | -1.85% |
|  | (-0.52 to -0.42) | (-0.52 to -0.4) | (0.12 to 0.38) |  | (-2.98 to -1.77) | (-3.3 to -2.08) | (-11.36 to 8.67) |
| Australasia | -0.33% | -0.28% | -0.14% |  | -2.95% | -3.06% | -1.71% |
|  | (-0.43 to -0.22) | (-0.39 to -0.17) | (-0.24 to -0.05) |  | (-3.65 to -2.25) | (-3.83 to -2.29) | (-4.45 to 1.12) |
| Caribbean | -0.45% | -0.45% | -0.06% |  | -0.73% | -0.76% | -0.56% |
|  | (-0.47 to -0.43) | (-0.47 to -0.43) | (-0.49 to 0.38) |  | (-0.84 to -0.63) | (-0.88 to -0.65) | (-1.05 to -0.08) |
| Central Asia | -0.03% | -0.02% | 0.17% |  | 2.57% | 2.40% | -5% |
|  | (-0.07 to 0) | (-0.05 to 0.02) | (-0.13 to 0.47) |  | (1.94 to 3.22) | (1.72 to 3.08) | (-21.45 to 14.9) |
| Central Europe | 0.37% | 0.42% | 0.31% |  | 1.61% | 1.45% | -2.84% |
|  | (0.29 to 0.44) | (0.34 to 0.49) | (-0.17 to 0.79) |  | (0.73 to 2.49) | (0.49 to 2.41) | (-9.91 to 4.79) |
| Central Latin America | -0.23% | -0.23% | 0.02% |  | -2.89% | -2.88% | -7.22% |
|  | (-0.26 to -0.2) | (-0.27 to -0.2) | (-0.02 to 0.07) |  | (-3.08 to -2.7) | (-3.09 to -2.68) | (-11.4 to -2.84) |
| Central Sub-Saharan Africa | -0.38% | -0.38% | 0.03% |  | -0.39% | -0.45% | 1.13% |
|  | (-0.41 to -0.36) | (-0.41 to -0.36) | (-0.24 to 0.31) |  | (-0.5 to -0.28) | (-0.56 to -0.34) | (0.69 to 1.56) |
| East Asia | 0.80% | 0.85% | 0.07% |  | 1.16% | 1.38% | 2.75% |
|  | (0.62 to 0.99) | (0.65 to 1.06) | (-0.07 to 0.22) |  | (0.49 to 1.84) | (0.66 to 2.1) | (2.51 to 2.99) |
| Eastern Europe | -0.37% | -0.32% | 0.02% |  | 4.68% | 4.49% | -0.80% |
|  | (-0.46 to -0.27) | (-0.42 to -0.23) | (-0.07 to 0.12) |  | (4.22 to 5.13) | (4.02 to 4.97) | (-1.16 to -0.43) |
| Eastern Sub-Saharan Africa | -0.52% | -0.49% | 0.11% |  | -0.59% | -0.61% | 0.42% |
|  | (-0.66 to -0.39) | (-0.64 to -0.34) | (-0.12 to 0.35) |  | (-0.67 to -0.5) | (-0.7 to -0.51) | (0.19 to 0.65) |
| High-income Asia Pacific | 0.14% | 0.18% | -0.02% |  | -3.03% | -3.37% | 1.02% |
|  | (0.05 to 0.22) | (0.09 to 0.27) | (-0.1 to 0.06) |  | (-3.51 to -2.55) | (-3.81 to -2.94) | (-0.7 to 2.78) |
| High-income North America | -0.18% | -0.22% | -0.77% |  | -2.27% | -2.29% | -4.93% |
|  | (-0.27 to -0.09) | (-0.32 to -0.12) | (-1.75 to 0.21) |  | (-2.46 to -2.07) | (-2.5 to -2.08) | (-11.81 to 2.49) |
| North Africa and Middle East | -0.02% | 0.08% | -0.07% |  | 0.26% | 0.24% | 0.58% |
|  | (-0.18 to 0.14) | (-0.08 to 0.24) | (-0.14 to 0) |  | (0.23 to 0.3) | (0.21 to 0.28) | (0.55 to 0.6) |
| Oceania | 0.10% | 0.10% | 0.10% |  | 0.01% | -0.01% | -0.64% |
|  | (0.09 to 0.11) | (0.09 to 0.11) | (-0.24 to 0.45) |  | (-0.05 to 0.08) | (-0.08 to 0.06) | (-1.01 to -0.26) |
| South Asia | 1.24% | 1.33% | 0.21% |  | -1.78% | -2.20% | 1.55% |
|  | (0.99 to 1.49) | (1.05 to 1.6) | (0.05 to 0.38) |  | (-2.5 to -1.06) | (-2.9 to -1.49) | (1.2 to 1.89) |
| Southeast Asia | 0.66% | 0.66% | 0.28% |  | 0.25% | 0.14% | 4.24% |
|  | (0.58 to 0.74) | (0.57 to 0.75) | (-0.13 to 0.68) |  | (0.08 to 0.43) | (-0.03 to 0.31) | (0.91 to 7.68) |
| Southern Latin America | 1.02% | 1.18% | 0.11% |  | 2.92% | 3.11% | -5.80% |
|  | (0.79 to 1.25) | (0.97 to 1.4) | (-0.24 to 0.45) |  | (2.41 to 3.43) | (2.58 to 3.65) | (-6.93 to -4.65) |
| Southern Sub-Saharan Africa | -0.37% | -0.31% | -0.12% |  | -0.13% | -0.16% | 1.68% |
|  | (-0.5 to -0.24) | (-0.45 to -0.17) | (-0.55 to 0.31) |  | (-0.3 to 0.04) | (-0.35 to 0.03) | (-0.18 to 3.57) |
| Tropical Latin America | 2.57% | 2.76% | 0% |  | 0.66% | 0.68% | -4.91% |
|  | (2.27 to 2.88) | (2.46 to 3.06) | (-0.08 to 0.07) |  | (0.53 to 0.79) | (0.54 to 0.81) | (-14.62 to 5.89) |
| Western Europe | 0.19% | 0.37% | -0.10% |  | -2.19% | -2.15% | -3.22% |
|  | (-0.14 to 0.51) | (0.04 to 0.71) | (-0.14 to -0.07) |  | (-2.55 to -1.83) | (-2.54 to -1.76) | (-6.63 to 0.33) |
| Western Sub-Saharan Africa | 0.11% | 0.15% | -0.02% |  | 0.95% | 0.86% | -0.51% |
|  | (0.04 to 0.17) | (0.08 to 0.21) | (-0.05 to 0.01) |  | (0.56 to 1.33) | (0.45 to 1.28) | (-0.58 to -0.44) |
